# National-, institutional-, and individual-level determinants of dental research excellence: an analysis of Stanford–Elsevier lists of the top 2% scholars worldwide (2017–2023)

**DOI:** 10.3389/froh.2025.1675102

**Published:** 2025-10-03

**Authors:** Abanoub Riad

**Affiliations:** 1 Masaryk Centre for Global Health (MCGH), Department of Public Health, Faculty of Medicine, Masaryk University, Brno, Czechia; 2 Department of Public Health, Faculty of Medicine, Masaryk University, Brno, Czechia

**Keywords:** academia, career ladder, dental education, dental research, gender equity, global burden of disease, health policy, macroeconomic factors

## Abstract

**Background:**

Research excellence, distinct from productivity, is a key criterion in science policy and institutional evaluation. This study examined global distribution and determinants of dental research excellence using the Stanford–Elsevier Lists (SEL) of the top 2% most-cited scientists.

**Methods:**

A bibliometric analysis was conducted using SEL datasets from 2017 to 2023. The analysis followed an ecological model consisting of three layers of independent variables: national-level indicators (macroeconomic metrics, oral disease burden, and development indices), institutional rankings, and individual-level variables (gender and academic age) were analysed. Descriptive statistics, multivariable regressions, and mixed-effects models were applied.

**Results:**

The analysis demonstrated a markedly uneven global distribution of excellent dental scholars (EDS), with 96.1% and 88.9% of career-long and single-year EDS, respectively, based in high-income countries. English-speaking countries dominated, reflecting historical and linguistic biases. Institutional elitism was apparent, with 20 universities accounting for nearly one-fifth of all EDS worldwide. Gender disparities persisted, with women comprising only 14.8% (*career-long*) and 18.1% (*single-year*). Academic age consistently predicted scholarly metrics more strongly than gender. EDS numbers correlated positively with macroeconomic indicators, particularly R&D investment, while oral disease burden was negatively correlated.

**Conclusions:**

Dental research excellence is disproportionately concentrated in high-income, English-speaking countries and elite institutions. Historic gender disparities remain, though narrowing trends are noticeable. The observed misalignment between oral disease burden and research excellence highlights the need for inclusive, needs-based research investment.

## 1 Introduction

Research excellence is increasingly recognised as a central concept in academia, shaping funding allocation, policy development, and institutional practices worldwide (1, 2). Originating in Europe, where it was adopted as a key criterion by the European Research Council (ERC), the concept has since been disseminated globally and applied across various disciplines, including the medical and health sciences such as dentistry (3, 4). While some scholars have criticised research excellence for its methodological limitations and conceptual ambiguity, it remains integral to contemporary science policy (5). Its appeal lies in offering a coherent, if contested, policy tool for evaluating scientific contributions; thus supporting competitive funding allocation, institutional benchmarking, and strategic agenda setting (3, 5)..

In the field of dentistry and oral health, meta-research and bibliometric studies have traditionally focused on research productivity, measured merely by publication and citation counts (6–8). An analysis of research productivity among members of the International Association for Dental Research (IADR) identified gender and academic age, i.e., time spent in a research career, as the strongest individual-level predictors (9). After adjusting for gender, academic age remained the most robust predictor of productivity (9). The IADR Distinguished Scientist Awards, widely regarded as indicators of research prestige, have historically exhibited significant gender disparity, with women comprising only 13% of awardees between 1955 and 2018 and remaining consistently underrepresented in relation to their share of the IADR membership (10). Moreover, prior empirical evidence has shown that macroeconomic factors, such as gross domestic product (GDP) per capita and the proportion of GDP allocated to research and education have a direct impact on national dental research productivity (6). Nevertheless, no study to date has examined research excellence among dental scholars using objective metrics of scholarly output, such as *h*-index, authorship position, or self-citations proportion.

The “science-wide author databases of standardized citation indicators” is a large-scale bibliometric initiative led by Professor *John P.A. Ioannidis* at *Stanford University*, developed in collaboration with *Elsevier*; therefore, referred to as the Stanford–Elsevier Lists (SEL) (11). It provides a publicly accessible lists ranking the top 2% of scientists globally across 22 scientific fields and 176 subfields, using a composite citation indicator that accounts for self-citations, author position, and co-authorship-adjusted metrics (11–13). The SEL include two principal components: the *career-long* impact list, which reflects cumulative scholarly influence over a scientist's entire publication history, and the *single-year* impact list, which captures citation performance within a specific year, allowing distinction between sustained and recent scientific impact (11, 12). Recent updates to the SEL included the integration of retraction data, allowing for more nuanced assessments of research credibility and impact (14–16). As a standardised, field-normalised, and methodologically transparent resource, the SEL offers a robust foundation for evaluating research excellence across disciplines and countries, while promoting responsible and contextual interpretation of citation-based metrics (13, 17).

The objectives of this study were (a) to assess the distribution of excellent dental scholars (EDS) globally and explore its association with national-level determinants, i.e., macroeconomic indicators, human development metrics, and oral disease burden, (b) to assess institutional-level determinants of dental research excellence, e.g., general and field-specific rankings in recognised databases, (c) to assess individual-level determinants of dental research excellence, i.e., academic age and gender, and (d) to evaluate temporal trends of EDS distribution between 2017 and 2023, focusing on gender, and official language.

## 2 Materials and methods

### 2.1 Study design

**Figure 1:**
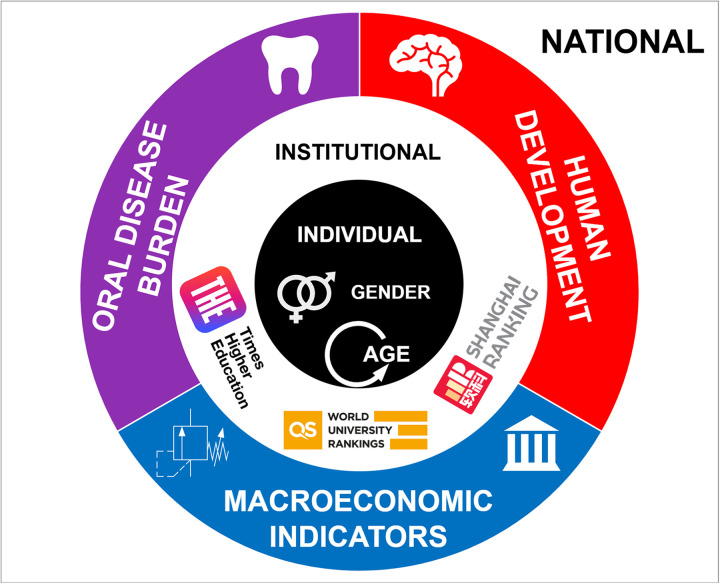
Theoretical framework of multilevel determinants shaping dental research excellence: individual-, institutional-, and national-level predictors.

This bibliometric study employed an ecological model to examine the global distribution of EDS included in the SEL of the top 2% most-cited scientists. The study is reported in accordance with the REporting of studies Conducted using Observational Routinely-collected Data (RECORD) guidelines (18). The conceptual framework of this study consists of three levels of dental research excellence determinants: national, institutional and individual Figure 1.

### 2.2 Data sources

The primary data source was the SEL, first released in July 2019 and incorporating citation metrics for the years 2017 and 2018. Subsequent updates were issued annually in October 2020, 2021, 2022, 2023, and 2024, covering citation metrics from 2017 to 2023. The raw datasets were obtained from the Elsevier Data Repository (19).

Complementary data sources for national- and institutional-level indicators included the following:

- Macroeconomic indicators, such as gross national income (GNI) per capita and research and development (R&D) expenditure as a percentage of GDP, were retrieved from the World Bank DataBank (20).
- Oral disease burden estimates, such as dental caries and edentulism, were obtained from the Global Health Data Exchange (GHDx) platform of the Institute for Health Metrics and Evaluation (IHME), as part of the Global Burden of Disease (GBD) Study (21).
- Human Development Index (HDI) values and their components, such as mean years of schooling and life expectancy, were downloaded from the United Nations Development Programme (UNDP) data centre (22).
- Economic level classifications of countries were based on the World Bank's fiscal year 2025 classification system (23).
- Official languages of countries were retrieved from the Central Intelligence Agency (CIA) World Factbook, as updated in January 2025 (24).
- Institutional rankings, including overall and discipline-specific indicators for medicine and dentistry, were obtained from the QS World University Rankings, Times Higher Education (THE) Rankings, and the Academic Ranking of World Universities (ARWU) (25–27).

### 2.3 Data cleaning and pre-processing

Both the *career-long* and *single-year* SEL datasets were downloaded and prepared for analysis. Dental scholars were identified as those whose disciplinary classification in either “subfield 1” or “subfield 2” was listed as *Dentistry*.

As the SEL datasets do not include gender information and provide only the scholars' names in the format “last name, first name” as stored in the Scopus database, gender was inferred algorithmically. Full names from the *career-long* lists (*n* = 11,023) and the *single-year* lists (*n* = 10,326) were processed using the genderizeR package in R. This tool predicts gender based on first names by aggregating data from publicly available user profiles across online platforms (28). While widely used in bibliometric research, its predictive accuracy varies depending on cultural and linguistic context (29).

Names that could not be classified with sufficient confidence using the R-based approach (*n* = 3,648 for the *career-long* lists; *n* = 2,647 for the *single-year* lists) were subsequently assessed using Claude Sonnet 3.7, a large language model developed by Anthropic AI. Nevertheless, a considerable number remained unclassified (*n* = 903 and *n* = 628, respectively), most of which comprised only initials or abbreviated given names, limiting algorithmic inference (30).

A manual review was conducted for the 50 most frequently represented institutions to harmonise names recorded in varying formats, including differences in language (official vs. English), length (full vs. abbreviated), and form (with or without acronyms). This step was undertaken to ensure consistency and accuracy in institutional-level analyses.

### 2.4 Independent variables

Independent variables were stratified into three levels: national, institutional, and individual. National-level determinants included: (i) macroeconomic and policy indicators, i.e., gross domestic product per capita (GDP), gross national income per capita (GNI), and the percentage of gross domestic product allocated to research and development (% R&D), healthcare (% Health), and education (% Education); (ii) human development metrics, i.e., Human Development Index (HDI), life expectancy, expected years of schooling, and mean years of schooling; and (iii) oral disease burden measured by disability-adjusted life years (DALYs) for deciduous dental caries, permanent dental caries, periodontal diseases, edentulism, oral and lip cancer, and other oral conditions.

Institutional-level determinants comprised global and discipline-specific rankings and scores from three major university ranking systems: QS (overall rank, dentistry rank, and dentistry score), Times Higher Education (THE; overall rank, medicine rank, and medicine score), and the Academic Ranking of World Universities (ARWU; overall rank, dentistry rank, and dentistry score).

Individual-level determinants included inferred gender (female vs. male) and academic age, calculated as the difference between the most recent and earliest years of publication listed in the Scopus database.

### 2.5 Dependent variables

Out of the variables provided in the SEL datasets, the number of EDS, their composite citation score (C-score), modified *h-*index, total citation count, and percentage of self-citations were selected for analysis.

The C-score is a composite indicator reflecting multiple dimensions of citation impact, including total citations, *h-*index, authorship position, and adjustments for co-authorship. The modified *h-*index accounts for authorship position, while the percentage of self-citations captures the proportion of citations attributed to the author's own publications (11, 12).

### 2.6 Statistical analyses

Descriptive statistics were performed using appropriate summary measures, and non-parametric tests (Chi-squared, Fisher's exact, Mann–Whitney U, Kruskal–Wallis, and Spearman's rho) were applied to assess group differences and correlations following normality testing by the Shapiro–Wilk test.

Multivariable linear regression models were constructed to examine the association of individual- and national-level determinants with scholarly metrics, including the composite citation score (C-score), modified *h-*index, total citation count, and percentage of self-citations. In addition, mixed-effects linear regression models were constructed with random intercepts specified for official language and World Bank income classification to account for unobserved heterogeneity across these contextual groupings.

Finally, binary and multinomial logistic regression analyses were performed to explore predictors of female gender group membership. A two-sided *p*-value < 0.05 was considered indicative of statistical significance.

## 3 Results

A total of 11,023 EDS were identified in the *career-long* Stanford–Elsevier Lists (SEL) between 2017 and 2023, compared to 10,326 EDS in the corresponding *single-year* lists; this minor discrepancy is due to the absence of a *single-year* SEL update for 2018. The number of EDS included in each annual SEL update increased steadily, from 803 to 2,218 (+176.2%) in the *career-long* lists, and from 628 to 2,261 (+259.9%) in the *single-year* lists between 2017 and 2023.

### 3.1 National-level analyses

According to the *career-long* SEL, 80% of EDS worldwide were concentrated in only 10 countries, all of which are high-income, with the largest share affiliated with the US (40.1%), followed by the UK (12.0%), Sweden (6.0%), Canada (3.8%), and Japan (3.6%). Similarly, in the *single-year* SEL, 80% of EDS were concentrated in only 13 countries, all high-income except for Brazil and China; the US again accounted for the largest global share (31.3%), followed by the UK (9.4%) and Italy (6.7%) Supplementary Tables 1, 2.

**Figure 2:**
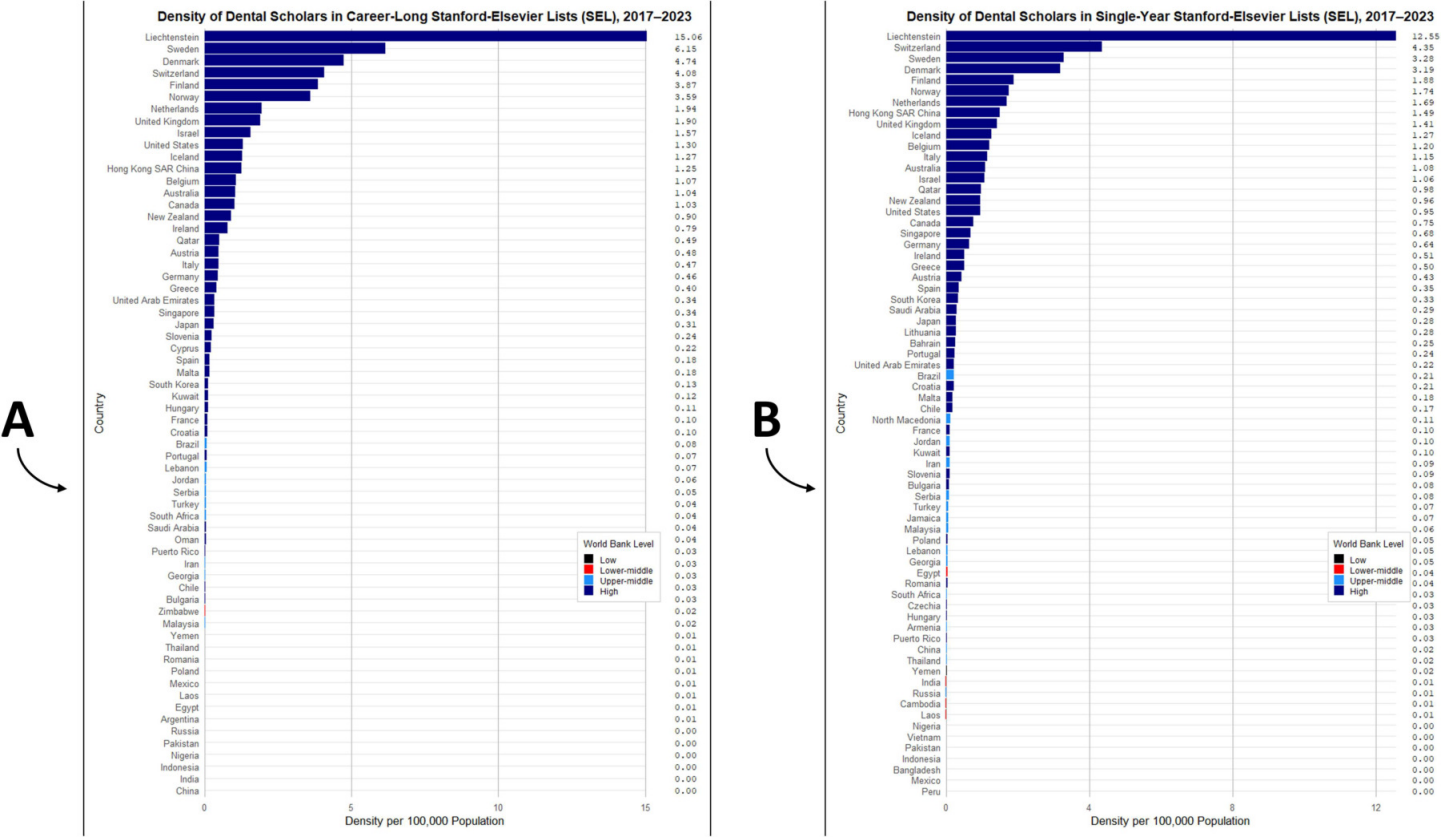
Density of excellent dental scholars (EDS) listed in the Stanford–Elsevier top 2% lists per 100,000 population, by country and world bank income classification (2017–2023); **(A)** *career-long* SEL and **(B)** *single-year* SEL.

The highest densities of EDS per 100,000 population were observed in high-income countries such as Liechtenstein (*career-long*: 15.06 and *single-year*: 12.55), Sweden (6.15 and 3.28), Denmark (4.74 and 3.19), Switzerland (4.08 and 4.35), Finland (3.87 and 1.88), Norway (3.59 and 1.74), and the Netherlands (1.94 and 1.69), while middle- and low-income countries showed substantially lower or near-zero densities. Figure 2.

In the *career-long* SEL, 96.1% of EDS were affiliated with high-income countries, vs. only <0.1% from low-income countries; 59.5% were based in English-speaking countries, followed by German- (7.3%), Swedish- (6%), and Dutch-speaking (4.4%) countries. Mean citation counts were highest among scholars from upper-middle-income countries (5,044), followed by those from high- (4,228), lower-middle- (2,052), and low-income (1,316) countries; by language, citation means were highest among Dutch-speaking countries (6,430), followed by Portuguese- (6,072) and Mandarin Chinese-speaking (5,844) countries Table 1.

**Table 1:** National-level analysis: distribution of excellent dental scholars (EDS) and their citation counts in the *career–long* and *single–year* Stanford-Elsevier lists (SEL) of top scientists worldwide (2017–2023), stratified by world bank classification (FY 2024) and official language (CIA world factbook).

Career–Long SEL										
	Variable	Outcome	SEL 2017	SEL 2018	SEL 2019	SEL 2020	SEL 2021	SEL 2022	SEL 2023	Total ▾
Scholars	World Bank	High	702 (99.0%)	738 (98.5%)	1,372 (97.0%)	1,692 (96.6%)	1,871 (95.8%)	1,963 (95.0%)	2,084 (94.5%)	10,422 (96.1%)
		Upper-middle	6 (0.8%)	11 (1.5%)	41 (2.9%)	53 (3.0%)	70 (3.6%)	87 (4.2%)	101 (4.6%)	369 (3.4%)
		Lower-middle	1 (0.1%)	0 (0.0%)	2 (0.1%)	6 (0.3%)	13 (0.7%)	15 (0.7%)	19 (0.9%)	56 (0.5%)
		Low	0 (0.0%)	0 (0.0%)	0 (0.0%)	0 (0.0%)	0 (0.0%)	1 (0.0%)	1 (0.0%)	2 (0.0%)
	Official Language	English	470 (66.3%)	500 (66.8%)	873 (61.7%)	1,035 (59.1%)	1,133 (58.0%)	1,182 (57.2%)	1,257 (57.0%)	6,450 (59.5%)
		German	48 (6.8%)	50 (6.7%)	100 (7.1%)	124 (7.1%)	149 (7.6%)	158 (7.6%)	166 (7.5%)	795 (7.3%)
		Swedish	40 (5.6%)	48 (6.4%)	91 (6.4%)	105 (6.0%)	117 (6.0%)	121 (5.9%)	126 (5.7%)	648 (6.0%)
		Dutch	31 (4.4%)	30 (4.0%)	62 (4.4%)	83 (4.7%)	84 (4.3%)	87 (4.2%)	95 (4.3%)	472 (4.4%)
		Japanese	18 (2.5%)	17 (2.3%)	45 (3.2%)	70 (4.0%)	75 (3.8%)	82 (4.0%)	83 (3.8%)	390 (3.6%)
		Danish	27 (3.8%)	22 (2.9%)	33 (2.3%)	46 (2.6%)	52 (2.7%)	51 (2.5%)	51 (2.3%)	282 (2.6%)
		Italian	8 (1.1%)	12 (1.6%)	33 (2.3%)	45 (2.6%)	49 (2.5%)	60 (2.9%)	73 (3.3%)	280 (2.6%)
		Finnish	16 (2.3%)	15 (2.0%)	27 (1.9%)	35 (2.0%)	40 (2.0%)	40 (1.9%)	43 (2.0%)	216 (2.0%)
		Norwegian	10 (1.4%)	12 (1.6%)	23 (1.6%)	36 (2.1%)	41 (2.1%)	38 (1.8%)	38 (1.7%)	198 (1.8%)
		Portuguese	2 (0.3%)	6 (0.8%)	23 (1.6%)	28 (1.6%)	37 (1.9%)	39 (1.9%)	49 (2.2%)	184 (1.7%)
		Hebrew	11 (1.6%)	8 (1.1%)	16 (1.1%)	24 (1.4%)	29 (1.5%)	33 (1.6%)	32 (1.5%)	153 (1.4%)
		Mandarin Chinese	3 (0.4%)	4 (0.5%)	12 (0.8%)	16 (0.9%)	25 (1.3%)	28 (1.4%)	30 (1.4%)	118 (1.1%)
		Spanish	3 (0.4%)	3 (0.4%)	15 (1.1%)	17 (1.0%)	20 (1.0%)	23 (1.1%)	25 (1.1%)	106 (1.0%)
		Arabic	3 (0.4%)	5 (0.7%)	11 (0.8%)	17 (1.0%)	16 (0.8%)	20 (1.0%)	25 (1.1%)	97 (0.9%)
		Chinese	6 (0.8%)	7 (0.9%)	11 (0.8%)	16 (0.9%)	19 (1.0%)	17 (0.8%)	18 (0.8%)	94 (0.9%)
		*Other*	13 (1.8%)	10 (1.3%)	40 (2.8%)	54 (3.1%)	68 (3.5%)	87 (4.2%)	94 (4.3%)	366 (3.4%)
Citations	World Bank	High	4,387.5 (3,023–7,189)	4,955 (3,456–8,025)	3,992 (2,521–6,467)	3,942 (2,485–6,348)	3,941 (2,470–6,483)	4,247 (2,656–6,942)	4,438 (2,782–7,293)	4,228 (2,668–6,882)
		Upper-middle	3,535 (2,400–3,739)	4,533 (4,224–9,257)	4,984 (3,098–6,979)	4,446 (2,326–6,703)	4,717 (2,727–6,907)	5,327 (2,870–8,088)	5,795 (3,065–8,943)	5,044 (2,857–7,866)
		Lower-middle	2,716 (2,716–2,716)	*NA*	5,457 (3,883–7,031)	1,122 (964–1,403)	1,517 (1,311–2,524)	1,893 (1,643–3,458)	2,537 (1,772–5,111)	2,052 (1,384–3,291)
		Low	*NA*	*NA*	*NA*	*NA*	*NA*	1,121 (1,121–1,121)	1,512 (1,512–1,512)	1,316 (1,219–1,414)
	Official Language	English	4,329 (2,895–6,894)	4,828 (3,311–7,636)	3,867 (2,428–6,357)	3,819 (2,400–6,255)	3,834 (2,366–6,390)	4,058 (2,555–6,894)	4,211 (2,656–7,292)	4,075 (2,583–6,784)
		German	4,862 (3,454–7,684)	5,448 (4,202–8,776)	4,694 (3,144–7,946)	4,916 (3,166–7,807)	4,917 (3,043–7,564)	5,464 (3,263–7,976)	5,621 (3,476–8,649)	5,176 (3,280–8,078)
		Swedish	4,894 (3,542–9,096)	4,677 (3,688–10,879)	3,750 (2,427–5,663)	3,790 (2,361–6,326)	3,703 (2,331–5,801)	3,665 (2,466–5,828)	3,698 (2,413–6,174)	3,868 (2,485–6,364)
		Dutch	6,805 (4,314–10,728)	6,710 (4,811–9,725)	6,154 (4,522–9,118)	6,045 (3,752–9,564)	6,375 (3,920–10,063)	6,650 (4,285–10,888)	6,916 (4,779–11,466)	6,430 (4,343–10,178)
		Japanese	4,692 (3,968–7,942)	5,022 (3,043–9,718)	4,313 (3,070–5,947)	4,480 (2,982–5,498)	4,651 (3,084–6,550)	5,014 (3,127–7,242)	5,029 (3,307–6,702)	4,758 (3,096–6,700)
		Danish	4,311 (2,524–5,818)	4,810 (3,230–6,231)	3,726 (2,861–6,317)	3,402 (2,467–5,804)	3,384 (2,540–5,881)	3,523 (2,626–6,412)	3,774 (2,846–6,705)	3,726 (2,613–6,234)
		Italian	5,606 (4,794–8,358)	6,528 (5,221–9,908)	4,420 (2,522–7,369)	4,265 (3,071–7,773)	4,277 (3,133–7,918)	4,984 (3,779–8,525)	5,649 (4,406–8,630)	5,132 (3,388–8,263)
		Finnish	6,209 (4,029–11,269)	5,622 (4,792–10,202)	3,643 (2,650–5,846)	3,942 (2,592–5,902)	4,048 (2,312–6,210)	3,988 (2,530–6,730)	4,239 (2,698–7,056)	4,232 (2,724–6,976)
		Norwegian	3,623 (2,804–5,684)	3,814 (3,020–5,564)	2,688 (1,984–3,638)	2,702 (1,954–3,651)	2,603 (1,958–3,856)	2,708 (2,024–4,216)	3,022 (2,097–4,504)	2,928 (2,014–4,244)
		Portuguese	3,688 (3,637–3,739)	6,087 (4,372–10,065)	4,799 (3,573–6,604)	5,803 (4,389–7,445)	5,850 (4,464–7,014)	6,601 (5,082–7,992)	7,270 (5,025–8,629)	6,072 (4,265–7,888)
		Hebrew	2,376 (1,890–3,724)	2,490 (2,119–3,755)	2,657 (2,274–3,094)	2,514 (2,101–3,790)	2,539 (2,096–3,795)	3,016 (2,323–4,229)	2,933 (2,236–3,735)	2,780 (2,127–3,748)
		Mandarin Chinese	2,365 (1,974–6,770)	3,478 (2,567–4,520)	4,862 (2,758–6,285)	4,132 (3,082–6,600)	5,391 (3,367–7,159)	6,982 (3,935–9,458)	7,489 (4,380–11,383)	5,844 (3,403–8,827)
		Spanish	6,556 (6,126–7,538)	8,189 (7,093–8,224)	4,492 (3,146–6,736)	5,228 (3,176–7,185)	4,260 (3,125–7,546)	4,134 (2,920–8,272)	4,798 (3,429–8,945)	4,908 (3,240–7,600)
		Arabic	3,553 (3,316–3,726)	5,033 (3,339–5,396)	2,088 (1,617–3,246)	2,462 (1,471–2,630)	2,247 (1,472–2,930)	2,406 (1,630–3,532)	3,235 (1,848–4,482)	2,600 (1,634–3,581)
		Chinese	3,358 (2,852–3,679)	4,150 (3,406–8,782)	4,138 (3,437–4,814)	4,855 (3,805–6,851)	5,076 (3,736–6,468)	5,440 (4,078–7,898)	5,674 (3,406–7,988)	4,614 (3,464–6,682)
		*Other*	3,693 (2,704–5,424)	4,488 (3,643–5,533)	3,520 (2,156–5,626)	2,863 (1,932–5,139)	2,719 (1,952–4,192)	3,175 (2,096–4,643)	3,122 (2,057–4,884)	3,210 (2,090–5,025)
Single–Year SEL										
	Variable	Outcome	SEL 2017	SEL 2019	SEL 2020	SEL 2021	SEL 2022	SEL 2023	Total ▾	
Scholars	World Bank	High	534 (95.5%)	1,293 (91.9%)	1,639 (90.0%)	1,789 (88.8%)	1,854 (87.2%)	1,943 (86.2%)	9,052 (88.9%)	
		Upper-middle	23 (4.1%)	102 (7.2%)	155 (8.5%)	188 (9.3%)	232 (10.9%)	267 (11.8%)	967 (9.5%)	
		Lower-middle	2 (0.4%)	11 (0.8%)	25 (1.4%)	36 (1.8%)	38 (1.8%)	43 (1.9%)	155 (1.5%)	
		Low	0 (0.0%)	1 (0.1%)	2 (0.1%)	1 (0.0%)	1 (0.0%)	1 (0.0%)	6 (0.1%)	
	Official Language	English	338 (60.5%)	722 (51.3%)	893 (49.0%)	938 (46.6%)	966 (45.5%)	1,000 (44.4%)	4,857 (47.7%)	
		German	59 (10.6%)	142 (10.1%)	173 (9.5%)	185 (9.2%)	201 (9.5%)	207 (9.2%)	967 (9.5%)	
		Italian	20 (3.6%)	91 (6.5%)	114 (6.3%)	142 (7.1%)	153 (7.2%)	160 (7.1%)	680 (6.7%)	
		Portuguese	12 (2.1%)	59 (4.2%)	79 (4.3%)	99 (4.9%)	110 (5.2%)	110 (4.9%)	469 (4.6%)	
		Dutch	28 (5.0%)	65 (4.6%)	76 (4.2%)	88 (4.4%)	88 (4.1%)	98 (4.3%)	443 (4.4%)	
		Mandarin Chinese	6 (1.1%)	33 (2.3%)	56 (3.1%)	66 (3.3%)	85 (4.0%)	113 (5.0%)	359 (3.5%)	
		Swedish	23 (4.1%)	64 (4.5%)	71 (3.9%)	64 (3.2%)	65 (3.1%)	59 (2.6%)	346 (3.4%)	
		Japanese	10 (1.8%)	47 (3.3%)	59 (3.2%)	72 (3.6%)	75 (3.5%)	81 (3.6%)	344 (3.4%)	
		Arabic	5 (0.9%)	18 (1.3%)	34 (1.9%)	43 (2.1%)	54 (2.5%)	62 (2.8%)	216 (2.1%)	
		Spanish	3 (0.5%)	20 (1.4%)	35 (1.9%)	50 (2.5%)	43 (2.0%)	53 (2.4%)	204 (2.0%)	
		Danish	17 (3.0%)	28 (2.0%)	39 (2.1%)	39 (1.9%)	33 (1.6%)	34 (1.5%)	190 (1.9%)	
		Korean	4 (0.7%)	19 (1.4%)	26 (1.4%)	31 (1.5%)	44 (2.1%)	48 (2.1%)	172 (1.7%)	
		Chinese	6 (1.1%)	21 (1.5%)	22 (1.2%)	22 (1.1%)	19 (0.9%)	22 (1.0%)	112 (1.1%)	
		Finnish	8 (1.4%)	13 (0.9%)	19 (1.0%)	23 (1.1%)	19 (0.9%)	23 (1.0%)	105 (1.0%)	
		Hebrew	6 (1.1%)	10 (0.7%)	20 (1.1%)	21 (1.0%)	24 (1.1%)	22 (1.0%)	103 (1.0%)	
		*Other*	14 (2.5%)	55 (3.9%)	105 (5.8%)	131 (6.5%)	146 (6.9%)	162 (7.2%)	613 (6.0%)	
Citations	World Bank	High	447 (310–683)	489 (312–751)	634 (373–1,014.5)	427 (265–697)	439 (279–735)	434 (278–716)	471 (298–769)	
		Upper-middle	490 (360–631)	617 (470–889)	766 (489–1,202)	536 (356–822)	547 (374–850)	548 (378–893)	577 (390–898)	
		Lower-middle	286 (245–326)	287 (193–334)	409 (334–650)	387 (218–721)	315 (208–818)	365 (197–1,026)	343 (208–752)	
		Low	*N/A*	188 (188–188)	319 (315–324)	239 (239–239)	303 (303–303)	385 (385–385)	306 (255–324)	
	Official Language	English	436 (300–649)	469 (290–740)	587 (348–994)	410 (238–684)	416 (260–715)	412 (256–708)	448 (278–756)	
		German	442 (324–728)	598 (416–810)	765 (515–1,111)	523 (368–805)	529 (346–804)	523 (324–788)	570 (368–861)	
		Italian	550 (336–652)	516 (357–684)	779 (526–1,029)	480 (341–714)	517 (353–799)	484 (348–736)	540 (361–800)	
		Portuguese	528 (462–605)	665 (537–874)	872 (624–1,178)	566 (414–800)	576 (432–828)	574 (441–892)	628 (463–892)	
		Dutch	664 (426–959)	738 (483–1,044)	1,000 (544–1,442)	636 (338–899)	635 (360–892)	600 (360–894)	695 (384–1,038)	
		Mandarin Chinese	576 (424–892)	770 (487–1,016)	870 (638–1,574)	546 (399–988)	587 (422–948)	584 (385–937)	637 (430–1,020)	
		Swedish	522 (346–959)	453 (246–832)	587 (288–994)	398 (254–835)	398 (236–762)	406 (210–889)	448 (259–888)	
		Japanese	456 (373–556)	568 (402–720)	731 (445–926)	462 (310–636)	488 (288–622)	451 (275–602)	500 (324–686)	
		Arabic	347 (336–367)	292 (232–487)	319 (162–697)	239 (142–464)	278 (162–528)	282 (194–568)	280 (168–540)	
		Spanish	574 (522–757)	628 (458–748)	624 (458–998)	454 (316–658)	480 (348–720)	463 (342–661)	504 (350–742)	
		Danish	321 (217–487)	390 (276–593)	508 (361–874)	349 (238–629)	309 (253–589)	342 (238–583)	377 (265–602)	
		Korean	330 (233–504)	472 (286–679)	516 (342–688)	363 (263–506)	376 (262–452)	366 (242–445)	386 (260–524)	
		Chinese	522 (381–668)	453 (329–809)	822 (522–1,321)	625 (346–889)	696 (398–1,024)	548 (364–729)	627 (360–942)	
		Finnish	728 (467–957)	460 (319–1,132)	689 (428–1,008)	554 (307–820)	536 (317–875)	550 (282–882)	554 (315–935)	
		Hebrew	197 (161–237)	246 (190–322)	374 (262–622)	267 (175–436)	248 (175–471)	331 (203–490)	271 (191–460)	
		*Other*	362 (258–622)	394 (230–533)	450 (289–694)	299 (199–494)	324 (233–558)	352 (226–632)	363 (230–579)	

Likewise, in the *single-year* SEL, 88.9% of EDS were affiliated with high-income countries vs. only 0.1% from low-income countries; 47.7% were based in English-speaking countries, followed by German- (9.5%), Italian- (6.7%), and Portuguese-speaking (4.6%) countries. Mean citation counts were highest among scholars from upper-middle-income countries (577), followed by those from high- (471), lower-middle- (343), and low-income (306) countries; by language, citation means were highest among scholars from Dutch-speaking countries (695), followed by Mandarin Chinese- (637) and Cantonese Chinese-speaking (627) countries. Table 1.

The number of EDS was strongly and positively correlated with % GDP allocated to research and development (*career-long ρ* = 0.739; *single-year ρ* = 0.706), and moderately correlated with HDI (*ρ* = 0.621 and 0.564), life expectancy (*ρ* = 0.608 and 0.549), GDP per capita (*ρ* = 0.604 and 0.527), % GDP for health (*ρ* = 0.538 and 0.497), and % GDP for education (*ρ* = 0.462 and 0.376). Conversely, negative correlations were observed with DALYs from caries of deciduous teeth (*ρ* = –0.527 and −0.495) and caries of permanent teeth (*ρ* = –0.375 and −0.312). Similar correlation patterns were observed for citation counts and were consistent across scholars classified under *Clinical Medicine*, the parent field of *Dentistry* Table 2.

**Table 2:** National– and institutional–level analyses: correlations between macroeconomic indicators, human development metrics, oral disease burden, and global university rankings with the number and citations of excellent dental scholars (EDS) in the Stanford–Elsevier lists (2017–2023).

National–level analysis (*N* = All countries)									
Group	Parameter	*Career–long* SEL				*Single–year* SEL			
		Clinical Medicine		Dentistry		Clinical Medicine		Dentistry	
		Scholars	Citations	Scholars	Citations	Scholars	Citations	Scholars	Citations
Macroeconomic Indicators	Gross National Income *per* Capita (GNI)	0.558**	0.541**	0.575**	0.571**	0.562**	0.529**	0.521**	0.459**
	Gross Domestic Product *per* Capita (GDP)	0.458**	0.462**	0.604**	0.605**	0.430**	0.399**	0.527**	0.475**
	% GDP Expenditure on Research	0.775**	0.772**	0.739**	0.749**	0.758**	0.743**	0.706**	0.638**
	% GDP Expenditure on Health	0.326**	0.330**	0.538**	0.521**	0.284**	0.244**	0.497**	0.469**
	% GDP Expenditure on Education	0.203*	0.178*	0.462**	0.429**	0.163	0.119	0.376**	0.313**
Human Development Metrics	Human Development Index (HDI)	0.603**	0.584**	0.621**	0.630**	0.589**	0.554**	0.564**	0.518**
	Life Expectancy	0.555**	0.543**	0.608**	0.603**	0.534**	0.483**	0.549**	0.515**
	Expected Schooling Years	0.587**	0.573**	0.534**	0.558**	0.558**	0.533**	0.499**	0.459**
	Mean Schooling Years	0.511**	0.485**	0.443**	0.461**	0.494**	0.468**	0.404**	0.362**
National Burden of Oral Disease**s**	Deciduous Teeth Caries	−0.466**	−0.455**	−0.527**	−0.536**	−0.458**	−0.406**	−0.495**	−0.437**
	Permanent Teeth Caries	−0.132	−0.147	−0.375**	−0.386**	−0.166*	−0.210**	−0.312**	−0.374**
	Periodontal Diseases	0.251**	0.259**	0.122	0.152	0.245**	0.188*	0.168	0.125
	Edentulism	0.352**	0.328**	0.152	0.187	0.344**	0.286**	0.167	0.118
	Oral & Lip Cancer	0.199*	0.165*	0.002	0.052	0.170*	0.145	0.110	0.100
	Other Oral Diseases	0.382**	0.361**	0.179	0.227	0.377**	0.320**	0.244*	0.189
Institutional–level analysis (*N* = top 20 institutions)									
Group	Parameter	*Career–long* SEL				*Single–year* SEL			
		Scholars		Citations		Scholars		Citations	
QS World University Rankings	Rank: General	−0.267		−0.143		−0.469*		−0.223	
	Rank: Dentistry	−0.067		−0.311		−0.197		−0.316	
	Score: Dentistry	0.129		0.176		0.059		0.168	
Times Higher Education (THE)	Rank: General	−0.349		−0.219		−0.576**		−0.318	
	Rank: Medicine	−0.411		−0.301		−0.601**		−0.405	
	Score: Medicine	0.411		0.301		0.601**		0.405	
Academic Ranking of World Universities (ARWU)	Rank: General	−0.505*		−0.219		−0.606**		−0.283	
	Rank: Dentistry	−0.484*		−0.668**		−0.675**		−0.633**	
	Score: Dentistry	0.544*		0.631**		0.676**		0.634**	

Spearman's rank correlation (*ρ*) was carried out with a significance level.

* *p* ≤ 0.05 level.

** *p* ≤ 0.01 level.

### 3.2 Institutional-level analyses

About 20% of the world's EDS were concentrated in only 20 institutions (*career-long*: 23.8%; *single-year*: 19.8%). This institutional elitism declined over time, from 29.8% to 22.3% in the *career-long* SEL (–7.5%) and from 24.7% to 20.0% in the *single-year* SEL (–4.7%) between 2017 and 2023. The leading institution was the University of Washington (*career-long*: 1.9%; *single-year*: 1.6% of the global share), followed by King's College London (1.7% and 1.4%), Harvard University (1.6% and 1.3%), and the University of Toronto (1.5% and 1.0%). The only institutions among the top 20 not located in North America or Western Europe were the University of São Paulo and the University of Hong Kong, which appeared in the *single-year* SEL but not in the *career-long* list, suggesting the emerging nature of dental research excellence in developing countries Table 3.

**Table 3:** Institution-Level analysis: distribution of excellent dental scholars (EDS) and their citation counts of Top 20 institutions in the Stanford-elsevier lists (SEL) of Top scientists worldwide (2,017–2,023).

Career–Long SEL: Total Scholar Count										
R	Institution (Acronym)	CTY	SEL 2017	SEL 2018	SEL 2019	SEL 2020	SEL 2021	SEL 2022	SEL 2023	Total ▾
1	University of Washington (UW)	USA	20 (2.5%)	16 (2.1%)	26 (1.8%)	34 (1.9%)	34 (1.7%)	39 (1.9%)	40 (1.8%)	209 (1.9%)
2	King's College London (KCL)	GBR	22 (2.7%)	9 (1.2%)	38 (2.7%)	28 (1.6%)	27 (1.4%)	26 (1.2%)	33 (1.5%)	183 (1.7%)
3	Harvard University (HU)	USA	21 (2.6%)	22 (2.9%)	24 (1.7%)	22 (1.2%)	26 (1.3%)	30 (1.4%)	35 (1.6%)	180 (1.6%)
4	University of Toronto (UofT)	CAN	17 (2.1%)	14 (1.9%)	23 (1.6%)	24 (1.4%)	28 (1.4%)	27 (1.3%)	27 (1.2%)	160 (1.5%)
5	University of California, San Francisco (UCSF)	USA	14 (1.7%)	14 (1.9%)	22 (1.5%)	27 (1.5%)	27 (1.4%)	27 (1.3%)	26 (1.2%)	157 (1.4%)
6	University of Michigan (U-M)	USA	14 (1.7%)	12 (1.6%)	21 (1.5%)	25 (1.4%)	29 (1.5%)	27 (1.3%)	28 (1.3%)	156 (1.4%)
7	Karolinska Institute (KI)	SWE	10 (1.2%)	19 (2.5%)	17 (1.2%)	24 (1.4%)	27 (1.4%)	27 (1.3%)	27 (1.2%)	151 (1.4%)
8	New York University (NYU)	USA	4 (0.5%)	11 (1.5%)	23 (1.6%)	22 (1.2%)	25 (1.3%)	26 (1.2%)	27 (1.2%)	138 (1.3%)
9	University of Zurich (UZH)	CHE	11 (1.4%)	8 (1.1%)	18 (1.3%)	21 (1.2%)	24 (1.2%)	24 (1.2%)	25 (1.1%)	131 (1.2%)
10	University of North Carolina (UNC)	USA	18 (2.2%)	16 (2.1%)	12 (0.8%)	20 (1.1%)	18 (0.9%)	24 (1.2%)	22 (1.0%)	130 (1.2%)
11	University of Texas (UT)	USA	13 (1.6%)	11 (1.5%)	18 (1.3%)	21 (1.2%)	21 (1.1%)	22 (1.1%)	22 (1.0%)	128 (1.2%)
12	University of Pennsylvania (UPenn)	USA	9 (1.1%)	11 (1.5%)	13 (0.9%)	17 (1.0%)	23 (1.2%)	27 (1.3%)	27 (1.2%)	127 (1.2%)
13	University of Florida (UF)	USA	13 (1.6%)	13 (1.7%)	19 (1.3%)	19 (1.1%)	19 (1.0%)	19 (0.9%)	18 (0.8%)	120 (1.1%)
14	University of Bern (UNIBE)	CHE	15 (1.9%)	13 (1.7%)	14 (1.0%)	15 (0.8%)	17 (0.9%)	19 (0.9%)	20 (0.9%)	113 (1.0%)
15	University of Oslo (UIO)	NOR	9 (1.1%)	9 (1.2%)	11 (0.8%)	19 (1.1%)	22 (1.1%)	20 (1.0%)	21 (0.9%)	111 (1.0%)
16	Academic Centre for Dentistry Amsterdam (ACTA)	NLD	0 (0.0%)	0 (0.0%)	14 (1.0%)	21 (1.2%)	22 (1.1%)	23 (1.1%)	16 (0.7%)	96 (0.9%)
17	The University of Hong Kong (HKU)	HKG	6 (0.7%)	7 (0.9%)	11 (0.8%)	16 (0.9%)	18 (0.9%)	16 (0.8%)	20 (0.9%)	94 (0.9%)
18	University of Gothenburg (GU)	SWE	0 (0.0%)	12 (1.6%)	8 (0.6%)	9 (0.5%)	10 (0.5%)	10 (0.5%)	36 (1.6%)	85 (0.8%)
19	Catholic University of Leuven (KU Leuven)	BEL	10 (1.2%)	7 (0.9%)	9 (0.6%)	12 (0.7%)	13 (0.7%)	12 (0.6%)	15 (0.7%)	78 (0.7%)
20	University College London (UCL)	GBR	13 (1.6%)	8 (1.1%)	0 (0.0%)	12 (0.7%)	17 (0.9%)	13 (0.6%)	10 (0.5%)	73 (0.7%)
Total (Top 20 Institutions)			239 (29.8%)	232 (30.9%)	341 (24.0%)	408 (23.0%)	447 (22.7%)	458 (22.0%)	495 (22.3%)	2,620 (23.8%)
Career–Long SEL: Average Citation Count										
R	Institution (Acronym)	CTY	SEL 2017	SEL 2018	SEL 2019	SEL 2020	SEL 2021	SEL 2022	SEL 2023	Total ▾
1	Catholic University of Leuven (KU Leuven)	BEL	12,173	12,917	14,254	13,477	13,654	15,038	16,256	14,153
2	University of Bern (UNIBE)	CHE	7,435	11,423	10,924	12,226	10,959	12,237	13,891	11,442
3	Harvard University (HU)	USA	9,529	10,131	9,583	8,041	10,284	9,397	9,833	9,574
4	University of Gothenburg (GU)	SWE	*NA*	8,472	7,718	7,722	4,990	9,471	9,082	8,288
5	University of Texas (UT)	USA	6,877	8,255	7,757	6,408	6,944	7,541	7,827	7,330
6	King's College London (KCL)	GBR	5,038	5,558	6,684	7,172	8,529	6,759	9,157	7,234
7	University of Michigan (U-M)	USA	5,906	6,903	7,171	7,015	6,005	7,283	7,180	6,816
8	University College London (UCL)	GBR	6,150	9,135	*NA*	6,851	6,700	5,979	6,406	6,725
9	University of California, San Francisco (UCSF)	USA	6,592	7,109	5,792	6,263	6,507	6,900	7,470	6,653
10	Academic Centre for Dentistry Amsterdam (ACTA)	NLD	*NA*	*NA*	4,946	6,016	6,111	6,547	7,921	6,327
11	The University of Hong Kong (HKU)	HKG	4,598	6,815	5,386	6,106	5,445	6,361	7,497	6,191
12	University of Toronto (UofT)	CAN	5,833	7,174	5,817	6,079	5,781	6,357	6,388	6,158
13	University of Zurich (UZH)	CHE	6,760	6,091	4,902	5,438	5,565	6,342	6,963	5,995
14	University of North Carolina (UNC)	USA	5,871	6,481	5,171	6,050	5,784	6,050	5,953	5,944
15	University of Washington (UW)	USA	6,117	6,430	5,677	6,220	5,401	5,959	5,568	5,852
16	University of Pennsylvania (UPenn)	USA	7,342	7,128	7,207	5,054	4,564	5,172	5,673	5,684
17	University of Florida (UF)	USA	5,300	5,994	4,844	5,444	5,616	6,168	5,257	5,507
18	Karolinska Institute (KI)	SWE	5,147	7,272	4,772	4,473	3,895	4,937	4,286	4,850
19	New York University (NYU)	USA	6,212	5,603	4,038	4,895	4,649	4,678	5,288	4,838
20	University of Oslo (UIO)	NOR	3,899	4,416	4,022	3,328	3,336	3,649	3,851	3,690
Total (Top 20 Institutions)			6,544	7,582	6,477	6,465	6,360	6,835	7,456	6,807
Single–Year SEL: Total Scholar Count										
R	Institution (Acronym)	CTY	SEL 2017	SEL 2019	SEL 2020	SEL 2021		SEL 2022	SEL 2023	Total ▾
1	University of Washington (UW)	USA	17 (2.7%)	25 (1.8%)	30 (1.6%)	31 (1.5%)		31 (1.5%)	29 (1.3%)	163 (1.6%)
2	King's College London (KCL)	GBR	16 (2.5%)	27 (1.9%)	26 (1.4%)	25 (1.2%)		25 (1.2%)	30 (1.3%)	149 (1.4%)
3	University of Bern (UNIBE)	CHE	17 (2.7%)	21 (1.5%)	25 (1.4%)	23 (1.1%)		28 (1.3%)	29 (1.3%)	143 (1.4%)
4	Harvard University (HU)	USA	14 (2.2%)	22 (1.5%)	21 (1.1%)	24 (1.2%)		24 (1.1%)	30 (1.3%)	135 (1.3%)
5	University of Michigan (U-M)	USA	10 (1.6%)	20 (1.4%)	24 (1.3%)	22 (1.1%)		22 (1.0%)	23 (1.0%)	121 (1.2%)
6	New York University (NYU)	USA	2 (0.3%)	20 (1.4%)	26 (1.4%)	23 (1.1%)		23 (1.1%)	25 (1.1%)	119 (1.2%)
7	University of Zurich (UZH)	CHE	11 (1.8%)	17 (1.2%)	20 (1.1%)	24 (1.2%)		23 (1.1%)	24 (1.1%)	119 (1.2%)
8	The University of Hong Kong (HKU)	HKG	6 (1.0%)	20 (1.4%)	22 (1.2%)	23 (1.1%)		21 (1.0%)	25 (1.1%)	117 (1.1%)
9	University of Pennsylvania (UPenn)	USA	6 (1.0%)	12 (0.8%)	18 (1.0%)	22 (1.1%)		28 (1.3%)	27 (1.2%)	113 (1.1%)
10	University of California, San Francisco (UCSF)	USA	9 (1.4%)	19 (1.3%)	22 (1.2%)	22 (1.1%)		20 (0.9%)	20 (0.9%)	112 (1.1%)
11	University of North Carolina (UNC)	USA	13 (2.1%)	11 (0.8%)	21 (1.1%)	21 (1.0%)		25 (1.2%)	20 (0.9%)	111 (1.1%)
12	University of São Paulo (USP)	BRA	3 (0.5%)	22 (1.5%)	28 (1.5%)	17 (0.8%)		17 (0.8%)	17 (0.8%)	104 (1.0%)
13	University of Toronto (UofT)	USA	10 (1.6%)	15 (1.1%)	17 (0.9%)	20 (1.0%)		19 (0.9%)	18 (0.8%)	99 (1.0%)
14	University of Texas (UT)	USA	6 (1.0%)	14 (1.0%)	16 (0.9%)	16 (0.8%)		17 (0.8%)	19 (0.8%)	88 (0.9%)
15	Catholic University of Leuven (KU Leuven)	BEL	11 (1.8%)	13 (0.9%)	13 (0.7%)	16 (0.8%)		15 (0.7%)	18 (0.8%)	86 (0.8%)
16	University of Milan (UNIMI)	ITA	3 (0.5%)	12 (0.8%)	12 (0.7%)	18 (0.9%)		17 (0.8%)	18 (0.8%)	80 (0.8%)
17	São Paulo State University (UNESP)	BRA	0 (0.0%)	7 (0.5%)	13 (0.7%)	13 (0.6%)		11 (0.5%)	17 (0.8%)	61 (0.6%)
18	Sichuan University (SCU)	CHN	0 (0.0%)	4 (0.3%)	11 (0.6%)	13 (0.6%)		3 (0.1%)	24 (1.1%)	55 (0.5%)
19	University of Gothenburg (GU)	SWE	0 (0.0%)	5 (0.4%)	4 (0.2%)	4 (0.2%)		6 (0.3%)	23 (1.0%)	42 (0.4%)
20	University of Amsterdam (UvA)	NEL	1 (0.2%)	1 (0.1%)	4 (0.2%)	3 (0.1%)		4 (0.2%)	17 (0.8%)	30 (0.3%)
Total (Top 20 Institutions)			155 (24.7%)	307 (21.5%)	373 (20.2%)	380 (18.8%)		379 (17.7%)	453 (20.0%)	2,047 (19.8%)
Single–Year SEL: Average Citation Count										
R	Institution (Acronym)	CTY	SEL 2017		SEL 2019	SEL 2020	SEL 2021	SEL 2022	SEL 2023	Total ▾
1	Sichuan University (SCU)	CHN	NA		1,273	1,810	1,343	1,339	1,088	1,320
2	King's College London (KCL)	GBR	1,112		1,561	1,605	1,298	627	928	1,192
3	Catholic University of Leuven (KU Leuven)	BEL	970		1,262	1,779	1,070	1,110	1,023	1,190
4	University of Texas (UT)	USA	694		1,205	1,686	1,260	1,261	656	1,160
5	Harvard University (HU)	USA	931		1,030	1,223	994	958	907	1,003
6	University of Bern (UNIBE)	CHE	642		994	1,323	940	931	1,006	991
7	University of Zurich (UZH)	CHE	812		862	1,152	716	779	851	858
8	University of Gothenburg (GU)	SWE	*NA*		909	1,315	415	851	777	820
9	The University of Hong Kong (HKU)	HKG	546		637	1,060	663	802	758	772
10	University of Michigan (U-M)	USA	607		728	951	626	743	726	746
11	University of São Paulo (USP)	BRA	552		688	860	582	586	753	707
12	University of Pennsylvania (UPenn)	USA	959		900	764	543	531	593	647
13	University of California, San Francisco (UCSF)	USA	678		655	892	574	573	481	642
14	University of Washington (UW)	USA	784		798	701	421	478	711	631
15	New York University (NYU)	USA	364		467	818	569	575	556	601
16	São Paulo State University (UNESP)	BRA	*NA*		632	756	539	564	519	595
17	University of Milan (UNIMI)	ITA	457		511	774	536	574	583	584
18	University of Amsterdam (UvA)	NEL	346		359	813	634	533	513	557
19	University of Toronto (UofT)	USA	451		538	746	469	475	467	526
20	University of North Carolina (UNC)	USA	496		449	800	492	469	375	520
Total (Top 20 Institutions)			742		854	1,063	735	703	733	807

The number of EDS and their citation counts were negatively correlated with university ranking positions and positively correlated with ranking scores, particularly within the subject-specific categories of *Dentistry* or *Medicine*. The strongest correlation was observed between the number of scholars in the *single-year* SEL and the ARWU database (rank: *ρ* = –0.675; score: *ρ* = 0.676), followed by THE (*ρ* = –0.601 and 0.601), whereas correlations with the QS ranking were not statistically significant (*ρ* = –0.197 and 0.059) Table 2.

### 3.3 Individual-level analyses

#### 3.3.1 Gender

On analysing gender, male dominance was evident in the *career-long* SEL (85.2% vs. 14.8%) and the *single-year* SEL (81.9% vs. 18.1%). Among countries with at least 50 scholars, the highest female proportions were observed in Finland (27.3%), France (24.2%), Sweden (22.0%), Denmark (21.9%), and the UK (20.1%) in the *career-long* SEL, and in Finland (32.3%), Denmark (31.5%), China (26.1%), Belgium (24.6%), and India (24.2%) in the *single-year* SEL. Contrarily, the lowest female representation was found in Australia (9.2%), the Netherlands (10.3%), Switzerland (10.5%), Hong Kong (10.5%), and Spain (10.8%) in the *career-long* SEL, and in Hong Kong (6.5%), Taiwan (10.8%), Turkey (11.5%), Canada (12.0%), and Saudi Arabia (12.4%) in the *single-year* SEL Supplementary Tables 3, 4.

Despite this pronounced gender gap in scholar numbers, the median citation count did not significantly differ between genders in either the *career-long* SEL (female: 4,287 vs. male: 4,233; *p* = 0.864) or the *single-year* SEL (472 vs. 280; *p* = 0.684). When examining gender-based differences in citation counts across SEL annual updates, World Bank income levels, and official language categories in both the *career-long* and *single-year* SEL, no statistically significant differences were observed (*p* > 0.05) Table 4.

**Table 4:** Individual-level analysis: sex and academic age of excellent dental scholars (EDS) in the Stanford-Elsevier lists (SEL) of top scientists worldwide (2017–2023).

*Career–long* SEL										
Variable	Outcome	Female			Male			*p*		
		Scholars: N (%)	Citations: Median (IQR)	Academic Age: Median (IQR)	Scholars: N (%)	Citations: Median (IQR)	Academic Age: Median (IQR)	Scholars	Citations	Age
Year	SEL 2017	83 (10.9%)	4,888 (3,352–7,921)	36 (29–41)	679 (89.1%)	4,283 (2,964–6,941)	37 (31–43)	**<0**.**001**	0.070	0.136
	SEL 2018	84 (11.7%)	5,280 (3,972–7,747)	35 (29–41)	636 (88.3%)	4,912 (3,380–8,188)	39 (32–45)		0.199	**<0**.**001**
	SEL 2019	171 (13.4%)	4,178 (2,625–6,552)	35 (27–41)	1,104 (86.6%)	3,993 (2,514–6,601)	38 (31–45)		0.481	**<0**.**001**
	SEL 2020	245 (15.2%)	4,132 (2,568–6,166)	35 (27–41)	1,368 (84.8%)	3,972 (2,471–6,526)	37 (31–44)		0.774	**<0**.**001**
	SEL 2021	298 (16.4%)	3,864 (2,476–5,897)	34.5 (27–40)	1,522 (83.6%)	3,998 (2,429–6,626)	37 (31–45)		0.401	**<0**.**001**
	SEL 2022	280 (14.7%)	4,266 (2,734–6,557)	34 (28–42)	1,619 (85.3%)	4,260 (2,630–7,024)	38 (31–45)		0.900	**<0**.**001**
	SEL 2023	338 (16.6%)	4,367 (2,828–7,094)	34 (28–40)	1,693 (83.4%)	4,473 (2,745–7,465)	38 (31–45)		0.732	**<0**.**001**
World Bank	High	1,440 (15.0%)	4,264 (2,727–6,549)	35 (28–41)	8,164 (85.0%)	4,249 (2,665–7,013)	38 (31–45)	0.631	0.808	**<0**.**001**
	Upper-middle	39 (13.0%)	6,117 (3,724–8,555)	24 (22–29)	261 (87.0%)	4,770 (2,759–7,473)	29 (23–42)		0.276	**0**.**002**
	Lower-middle	9 (18.4%)	1,848 (1,311–2,524)	14 (13–30)	40 (81.6%)	2,310 (1,463–3,291)	22 (19–31)		0.638	0.102
	Low	0 (0.0%)	NA	NA	2 (100.0%)	1,317 (1,219–1,414)	10 (9–10)		NA	NA
Official Language	English	900 (14.8%)	4,062 (2,670–6,067)	34 (27–40)	5,163 (85.2%)	4,106 (2,582–6,980)	39 (32–46)	**<0**.**001**	0.198	**<0**.**001**
	German	78 (10.6%)	5,442 (4,121–7,502)	26 (19–32)	661 (89.4%)	5,100 (3,261–8,188)	33 (26–40)		0.389	**<0**.**001**
	Swedish	126 (22.0%)	4,832 (2,696–7,598)	40 (35–45)	447 (78.0%)	3,794 (2,439–5,845)	40 (33–45)		0.060	0.351
	Dutch	50 (12.3%)	7,109 (5,452–11,353)	33 (21–36)	355 (87.7%)	6,579 (4,355–10,341)	36 (33–42)		0.271	**<0**.**001**
	Japanese	43 (12.2%)	5,529 (3,600– 10,144)	38 (32–42)	310 (87.8%)	4,679 (3,099–6,638)	37 (33–41)		**0**.**019**	0.832
	Danish	55 (21.9%)	3,994 (2,976–5,607)	37 (33–43)	196 (78.1%)	3,735 (2,623–6,307)	41 (33–49)		0.712	**0**.**009**
	Italian	39 (14.8%)	5,764 (2,961–7,191)	35 (22–39)	225 (85.2%)	5,015 (3,377–8,691)	32 (26–36)		0.535	0.398
	Finnish	51 (27.3%)	4,123 (2,128–14,538)	38 (32–45)	136 (72.7%)	4,214 (2,845–6,464)	35 (30–45)		0.564	0.905
	Norwegian	28 (16.7%)	2,956 (2,152–4,288)	43 (35–47)	140 (83.3%)	2,822 (1,978–4,337)	39 (35–46)		0.570	0.346
	Portuguese	28 (18.8%)	6,281 (4,968–8,869)	24 (22–27)	121 (81.2%)	5,850 (4,129–7,641)	27 (23–37)		0.208	**0**.**019**
	Hebrew	17 (11.4%)	2,412 (2,211–2,736)	42 (32–47)	132 (88.6%)	2,868 (2,122–4,145)	38 (34–44)		0.112	0.340
	Mandarin Chinese	10 (11.0%)	6,925 (4,144–7,734)	20 (19–30)	81 (89.0%)	5,272 (3,386–10,217)	30 (23–35)		0.694	0.074
	Spanish	10 (10.2%)	5,175 (3,124–7,516)	32 (28–34)	88 (89.8%)	5,222 (3,345–8,196)	35 (30–40)		0.769	0.242
	Arabic	7 (8.0%)	1,617 (1,453–4,545)	14 (13–33)	81 (92.0%)	2,600 (1,639–3,557)	30 (27–36)		0.483	0.276
	Chinese	8 (10.5%)	4,154 (3,451–6,108)	31 (29–36)	68 (89.5%)	5,095 (3,468–7,072)	31 (26–34)		0.554	1.000
	*Other*	49 (10.5%)	2,976 (2,435–5,057)	34 (26–43)	417 (89.5%)	3,274 (2,079–5,149)	34 (25–44)		0.384	0.276
Total		1,499 (14.8%)	4,287 (2,721–6,624)	35 (28–41)	8,621 (85.2%)	4,233 (2,656–6,993)	38 (31–45)	10,120 (91.8%)	0.864	**<0**.**001**
*Single–year* SEL										
Variable	Outcome	Female			Male			*p.*		
		Scholars: N (%)	Citations: Median (IQR)	Academic Age: Median (IQR)	Scholars: N (%)	Citations: Median (IQR)	Academic Age: Median (IQR)	Scholars	Citations	Age
Year	SEL 2017	96 (15.7%)	416 (266–611)	*NA*	514 (84.3%)	451 (313–693)	*NA*	**0**.**020**	0.180	*NA*
	SEL 2019	227 (17.1%)	494 (328–762)	27 (20–35)	1,097 (82.9%)	491 (312–753)	32 (22–40)		0.581	**<0**.**001**
	SEL 2020	304 (17.6%)	664 (397–1,050)	26 (18–36)	1,421 (82.4%)	634 (369–1,020)	31 (20–40)		0.274	**<0**.**001**
	SEL 2021	327 (17.1%)	460 (303–688)	25 (18–35)	1,584 (82.9%)	430 (260–713)	30 (20–39)		0.290	**<0**.**001**
	SEL 2022	364 (18.2%)	440 (301–699)	25 (18–34)	1,640 (81.8%)	445 (281–760)	31 (20–40)		0.757	**<0**.**001**
	SEL 2023	437 (20.6%)	410 (273–663)	26 (19–35)	1,687 (79.4%)	451 (286–752)	30 (20–40)		0.072	**<0**.**001**
World Bank	High	1,539 (18.0%)	460 (303–737)	27 (19–36)	7,027 (82.0%)	473 (297–774)	32 (22–40)	0.199	0.337	**<0**.**001**
	Upper-middle	160 (18.8%)	604 (405–988)	20.5 (16–26)	690 (81.2%)	570 (374–881)	20 (16–27)		0.063	0.913
	Lower-middle	35 (24.1%)	364 (253–513)	14 (11.5–17)	110 (75.9%)	339 (203–843)	14 (11–18)		0.969	0.808
	Low	0 (0.0%)	*NA*	*NA*	5 (100.0%)	310 (303–328)	9 (8–10)		*NA*	*NA*
Official Language	English	795 (17.1%)	435 (286–718)	27 (21–36)	3,853 (82.9%)	450 (277–759)	35 (25–44)	**<0**.**001**	0.929	**<0**.**001**
	German	136 (14.8%)	485 (360–710)	21 (16–30)	780 (85.2%)	588 (368–877)	25 (18–34)		**0**.**048**	**0**.**001**
	Italian	157 (23.5%)	520 (359–730)	23 (16–33)	512 (76.5%)	542 (362–824)	26 (17–34)		0.371	0.156
	Portuguese	76 (18.7%)	596 (479–994)	22 (18–26)	331 (81.3%)	619 (456–873)	21 (18–27)		0.558	0.487
	Dutch	72 (17.8%)	739 (421–996)	25 (19–35)	333 (82.2%)	678 (368–1,061)	34 (25–40)		0.954	**<0**.**001**
	Mandarin Chinese	70 (22.9%)	688 (455–1,090)	20 (16–26)	236 (77.1%)	647 (431–1,025)	21 (15–29)		0.440	0.570
	Swedish	75 (23.4%)	383 (265–1,027)	37 (26–44)	245 (76.6%)	462 (262–873)	37 (27–43)		0.953	0.557
	Japanese	50 (15.4%)	525 (408–692)	32 (28–38)	275 (84.6%)	488 (312–680)	32 (26–37)		0.242	0.612
	Arabic	30 (15.1%)	274 (104–741)	13 (11–16)	169 (84.9%)	282 (185–510)	14 (10–19)		0.415	0.332
	Spanish	28 (14.7%)	563 (442–877)	26 (19–31)	163 (85.3%)	503 (350–747)	25 (16–34)		0.274	0.925
	Danish	58 (31.5%)	369 (274–590)	37 (33–45)	126 (68.5%)	363 (259–590)	40 (27–49)		0.949	0.879
	Korean	24 (17.0%)	338 (242–457)	18 (16–24)	117 (83.0%)	388 (265–515)	21 (16–25)		0.186	0.563
	Chinese	6 (6.5%)	745 (529–1,140)	31 (27–33)	87 (93.5%)	630 (359–937)	26 (21–33)		0.416	0.431
	Finnish	31 (32.3%)	674 (455–1,081)	43 (33–45)	65 (67.7%)	536 (309–926)	31 (27–39)		0.062	**0**.**019**
	Hebrew	19 (18.6%)	357 (160–422)	26 (21–46)	83 (81.4%)	271 (202–482)	37 (24–41)		0.300	0.238
	Other	107 (19.0%)	348 (200–548)	19 (14–26)	457 (81.0%)	368 (235–591)	23 (15–36)		0.171	0.058
Total		1,755 (18.1%)	472 (309–548)	26 (18–35)	7,943 (81.9%)	480 (299–786)	31 (20–40)	9,698 (93.9%)	0.684	**<0**.**001**

Chi-squared (*χ^2^*) test, Fisher's exact test, and Mann–Whitney (*U*) test were used with a significance level *p* ≤ 0.05.

Bold font indicates statistically significant values (*p* < 0.05).

Further gender-based analysis of scholarly output metrics indicated that in the *career-long* SEL, male scholars had significantly higher C-scores (3.40 vs. 3.35; *p* < 0.001) and modified *h-*indices (16.47 vs. 15.69; *p* < 0.001), while female scholars had a higher self-citation percentage (0.10 vs. 0.09; *p* = 0.002). Similarly, in the *single-year* SEL, males exhibited significantly higher C-scores (2.59 vs. 2.58; *p* = 0.001) and modified *h-*indices (4.92 vs. 4.88; *p* = 0.002), whereas females had higher self-citation percentages (0.09 vs. 0.08; *p* = 0.002) Supplementary Table 5.

#### 3.3.2 Academic age

Academic age, which was used in this study as a proxy for scholar age, had a median of 37 years [IQR: 30–44] in the *career-long* SEL, notably longer than the 29 years [20–39] observed in the single-year SEL. Among countries with at least 50 scholars in the career-long SEL, the shortest median academic ages were observed in South Korea [24 (21–29.5)], Brazil [26 (22–34)], Taiwan [26 (22–32)], China [31 (26.5–35.25)], and Germany [31 (25–39)], whereas the longest were in France [44 (27–48)], Sweden [40 (33–45)], Denmark [40 (33–48)], the US [39 (32–46)], and Norway [39 (35–45.75)]. Likewise, in the single-year SEL, the shortest median academic ages were observed in Saudi Arabia [12 (8–16)], India [15 (12–18)], Iran [16 (13–19)], China [20 (15–26)], and Turkey [20 (12.5–25.5)], while the longest were found in Denmark [39 (29–48)], Norway [38.5 (33.75–44.25)], Sweden [37 (27–43)], Israel [36 (23–42)], and the US [35 (24–43)] Supplementary Tables 6, 7.

Among all scholarly output metrics established by the SEL methodology, the number of single-authored publications exhibited the strongest positive correlation with academic age (*career-long ρ* = 0.404; *single-year ρ* = 0.452), whereas the percentage of self-citations was the only metric negatively correlated with academic age (*ρ* = –0.232 and −0.361, respectively) Supplementary Table 8.

Male scholars had significantly longer academic ages in both the *career-long* SEL (38 vs. 35 years; *p* < 0.001) and the *single-year* SEL (31 vs. 26; *p* < 0.001). This pattern was consistently observed across all SEL annual updates and within high-income countries, except for Finland where females had longer academic age in the *single-year* SEL (43 vs. 31; *p* < 0.001) Table 4.

### 3.4 Time-trend analyses

**Figure 3:**
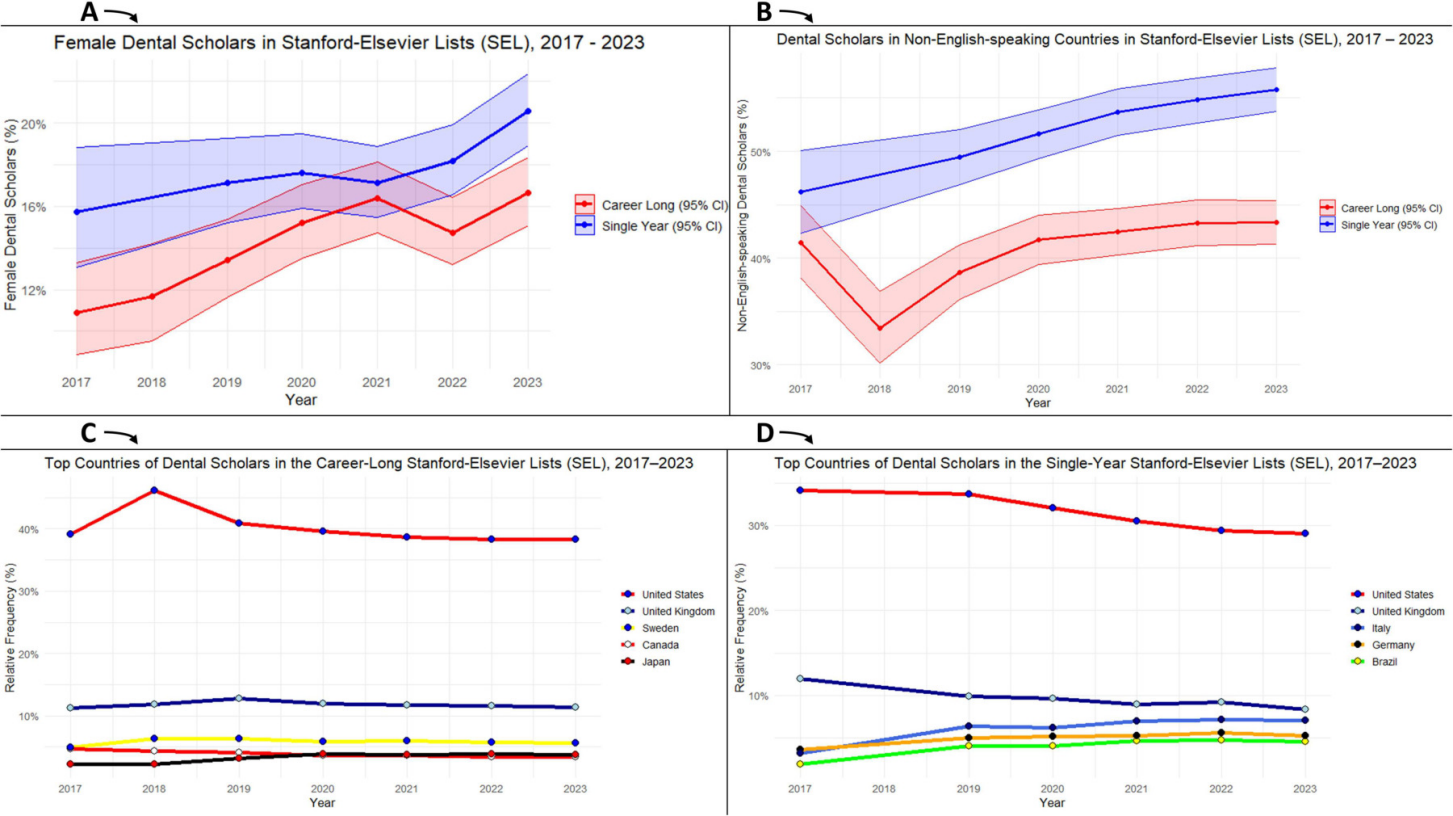
Time trends in dental research excellence in the Stanford–Elsevier top 2% lists (2017–2023); **(A)** female representation, **(B)** representation of non-English-speaking countries, **(C)** top countries in the *career-long* SEL, and **(D)** top countries in the *single-year* SEL.

Tracking changes from SEL 2017 to SEL 2023, the proportion of female scholars gradually increased in both the *career-long* (10.9% vs. 16.6%) and *single-year* (15.7% vs. 20.6%) lists. Moreover, the proportion of scholars based in non-English-speaking countries rose in the *career-long* (33.7% vs. 43.0%) and *single-year* (39.5% vs. 55.6%) lists. Similarly, the proportion of scholars based in countries outside the high-income group increased in the *career-long* (1.0% vs. 5.5%) and *single-year* (4.5% vs. 13.8%) lists Figure 3.

### 3.5 Gender gap in dental research excellence

To better understand the gender gap in dental research excellence, logistic regression models were constructed to identify factors associated with female group membership among EDS. The analysis revealed that female gender was significantly associated with shorter academic age; for each additional year of scholarly activity, the odds of female group membership decreased [*career-long*: OR = 0.967 (95% CI: 0.962–0.973); *single-year*: OR = 0.977 (0.973–0.981)]. Consistently, female gender was also significantly associated with lower scholarly output metrics, except for citation count. This included C-score [0.629 (0.520–0.761); 0.727 (0.617–0.856)], modified *h-*index [0.968 (0.959–0.978); 0.954 (0.925–0.984)], and percentage of self-citations [4.293 (1.805–10.209); 2.479 (1.360–4.517)].

The likelihood of being female increased with higher national investment in education, as each one-point increase in GDP share allocated to education was associated with higher odds of female representation [1.238 (1.168–1.312); 1.102 (1.040–1.167)]. Contrarily, higher national burdens of deciduous caries [0.643 (0.499–0.827); 0.715 (0.581–0.880)], permanent caries [0.976 (0.965–0.987); 0.990 (0.981–0.999)], and periodontal disease [0.994 (0.991–0.997); 0.997 (0.995–0.999)] were associated with lower odds of female representation Supplementary Table 9.

### 3.6 Social and macroeconomic determinants of dental research excellence

Mixed-effects linear regression models were constructed for each scholarly output metric (C-score, modified *h-*index, citation count, and self-citation percentage). World Bank income classification and official language were specified as fixed effects to evaluate their consistent and group-distinguishing influence on scholarly productivity across countries, as they represent broad structural determinants that are stable and theoretically grounded. In contrast, other national-level indicators (such as life expectancy, disease burden from deciduous caries, permanent caries, and edentulism, and the percentages of GDP spent on research, health, and education) were included as continuous covariates. Individual-level indicators (gender and academic age) were also controlled for. Country was treated as a random intercept to account for clustering and unobserved heterogeneity at the national level Table 5.

**Table 5:** Individual-level analysis: mixed-effects regression models of scholarly outputs of excellent dental scholars (EDS) in the Stanford-Elsevier lists (SEL) of Top scientists worldwide (2017–2023).

Model fit	*Career–long* SEL							
	Composite score (C)		Modified H-index		Citations count		% Self-citations	
Official Language as Intercept: ICC	12.1%		17%		13.8%		33.5%	
World Bank Level as Intercept: ICC	7%		5.9%		31.7%		0%	
Predictor	Adj. *β* (95% CI)	*p.*	Adj. *β* (95% CI)	*p.*	Adj. *β* (95% CI)	*p.*	Adj. *β* (95% CI)	*p.*
Gender (Male vs. Female)	0.02 (0.002–0.034)	**0**.**030**	0.55 (0.23–0.88)	**<0**.**001**	73.53 (−214.45 to −361.5)	0.617	−1.3 × 10^−^³ (−4.4 × 10^−^³ to−1.8 × 10^−^³)	0.408
Academic Age (per Year)	0.01 (0.007–0.008)	**<0**.**001**	0.18 (0.17–0.19)	**<0**.**001**	48.41 (38.37–58.45)	**<0**.**001**	−1.1 × 10^−^³ (−1.2 × 10^−^³ to −9.9 × 10^−^⁴)	**<0**.**001**
Life Expectancy (per Year)	0.02 (0.010–0.021)	**<0**.**001**	0.31 (0.2–0.43)	**<0**.**001**	325.81 (209.74–441.88)	**<0**.**001**	3.2 × 10^−^⁴ (−4.3 × 10^−^⁴ to −1.1 × 10^−^³)	0.398
Deciduous Caries (per DALY/100 K)	0.01 (−0.015 to –0.042)	0.347	0.11 (−0.49 to –0.71)	0.721	321.48 (−204.36 to –847.32)	0.232	6.1 × 10^−^⁴ (−5.2 × 10^−^³−6.4 × 10^−^³)	0.837
Permanent Caries (per DALY/100 K)	0.003 (0.001–0.004)	**0**.**005**	0.02 (−0.02 to –0.06)	0.328	27.47 (−6.39 to –61.33)	0.113	−2.7 × 10^−^⁴ (−6.6 × 10^−^⁴ to− 1.1 × 10^−^⁴)	0.168
Edentulism (per DALY/100 K)	−0.001 (−0.001 to −0.0003)	**<0**.**001**	−0.01 (−0.01 to −0.002)	**0**.**014**	−6.39 (−11.59 to −1.19)	**0**.**016**	1.2 × 10^−^⁴ (6.4 × 10^−^⁵ to −1.8 × 10^−^⁴)	**<0**.**001**
GDP Expenditure on R & D (per %)	0.01 (−0.020 to –0.034)	0.606	−0.07 (−0.67 to –0.54)	0.830	289.15 (−216.49 to –794.79)	0.266	−6.7 × 10^−^³ (−1.3 × 10^−^² to −2.4 × 10^−^⁴)	**0**.**043**
GDP Expenditure on Health (*per* %)	0.01 (0.006–0.018)	**<0**.**001**	0.04 (−0.1 to –0.17)	0.579	173.33 (52.81–293.85)	**0**.**005**	−1.4 × 10^−^³ (−2.7 × 10^−^³ to −1.0 × 10^−^⁵)	**0**.**049**
GDP Expenditure on Education (*per* %)	−0.001 (−0.023 to –0.021)	0.929	−0.16 (−0.63 to –0.31)	0.508	314.44 (−97.43 to −726.31)	0.137	4.5 × 10^−^³ (−1.1 × 10^−^⁴ to 9.1 × 10^−^³)	0.056
Model Fit	Single–Year SEL							
	Composite Score (C)		Modified H-index		Citations Count		% Self-citations	
Official Language as Intercept: ICC	15.2%		18.6%		41.3%		25.7%	
World Bank Level as Intercept: ICC	25.2%		24.1%		79.2%		0%	
Predictor	Adj. *β* (95% CI)	*p.*	Adj. *β* (95% CI)	*p.*	Adj. *β* (95% CI)	*p.*	Adj. *β* (95% CI)	*p.*
Gender (Male vs. Female)	0.02 (−7.92 × 10^−^⁴ to −0.03)	0.062	0.03 (−0.06 to –0.12)	0.476	84.25 (34.08–134.43)	**0**.**001**	1.7 × 10^−^⁴ (−3.7 × 10^−^³to −4.1 × 10^−^³)	0.932
Academic Age (*per* Year)	0.004 (0.004–0.01)	**<0**.**001**	0.03 (0.02–0.03)	**<0**.**001**	−2.26 (−3.9 to −0.62)	**0**.**007**	−1.9 × 10^−^³ (−2.0 × 10^−^³ to −1.8 × 10^−^³)	**<0**.**001**
Life Expectancy (*per* Year)	0.01 (0.01–0.02)	**<0**.**001**	0.14 (0.1–0.18)	**<0**.**001**	62.13 (37.79–86.47)	**<0**.**001**	3.5 × 10^−^⁴ (−7.5 × 10^−^⁴ to −1.4 × 10^−^³)	0.534
Deciduous Caries (*per* DALY/100 K)	0.01 (−0.02 to −0.04)	0.569	0.08 (−0.09 to –0.25)	0.373	74.59 (−30.32 to −179.51)	0.164	4.7 × 10^−^³ (−2.8 × 10^−^³ to −1.2 × 10^−^²)	0.218
Permanent Caries (*per* DALY/100 K)	0.004 (0.003–0.01)	**<0**.**001**	0.02 (0.01–0.04)	**<0**.**001**	2.26 (−4.43 to −8.95)	0.508	−2.9 × 10^−^⁴ (−7.5 × 10^−^⁴ to −1.6 × 10^−^⁴)	0.210
Edentulism (*per* DALY/100 K)	−1.14 × 10^−^⁴ (−4.24 × 10^−^⁴ to –0.)	0.469	−8.82 × 10^−^⁴ (−0.003 to –8.19 × 10^−^⁴)	0.310	1.26 (0.24–2.28)	**0**.**016**	1.3 × 10^−^⁴ (5.7 × 10^−^⁵ to −2.1 × 10^−^⁴)	**<0**.**001**
GDP Expenditure on R & D (*per* %)	0.03 (0.003–0.06)	**0**.**032**	−0.05 (−0.21 to –0.12)	0.561	93.4 (−14.97 to −201.76)	0.092	−3.1 × 10^−^³ (−1.1 × 10^−^² to −4.5 × 10^−^³)	0.423
GDP Expenditure on Health (*per* %)	0.01 (−9.33 × 10^−^⁴ to –0.01)	0.090	0.07 (0.03–0.11)	**<0**.**001**	7.86 (−16.18 to −31.89)	0.522	−1.7 × 10^−^³ (−3.4 × 10^−^³ to −−1.1 × 10^−^⁴)	**0**.**037**
GDP Expenditure on Education (*per* %)	0. (−0.03 to –0.02)	0.783	−0.06 (−0.19 to –0.06)	0.319	−5.46 (−87.07 to −76.14)	0.896	8.7 × 10^−^⁴ (−4.6 × 10^−^³ to −6.4 × 10^−^³)	0.757

Bold font indicates statistically significant values (*p* < 0.05).

In the *career-long* SEL, official language accounted for a substantial portion of the variance in self-citation percentage (ICC = 33.5%), modified *h-*index (17.0%), and C-score (12.1%), whereas World Bank income level showed the strongest effect for citation count (31.7%). Moreover, the *single-year* SEL demonstrated stronger clustering by World Bank level, which explained the majority of the variance in citation count (79.2%) and considerable portions in C-score (25.2%) and modified *h-*index (24.1%), while official language continued to exert notable influence on citation count (41.3%) and self-citation percentage (25.7%) Table 5.

**Figure 4:**
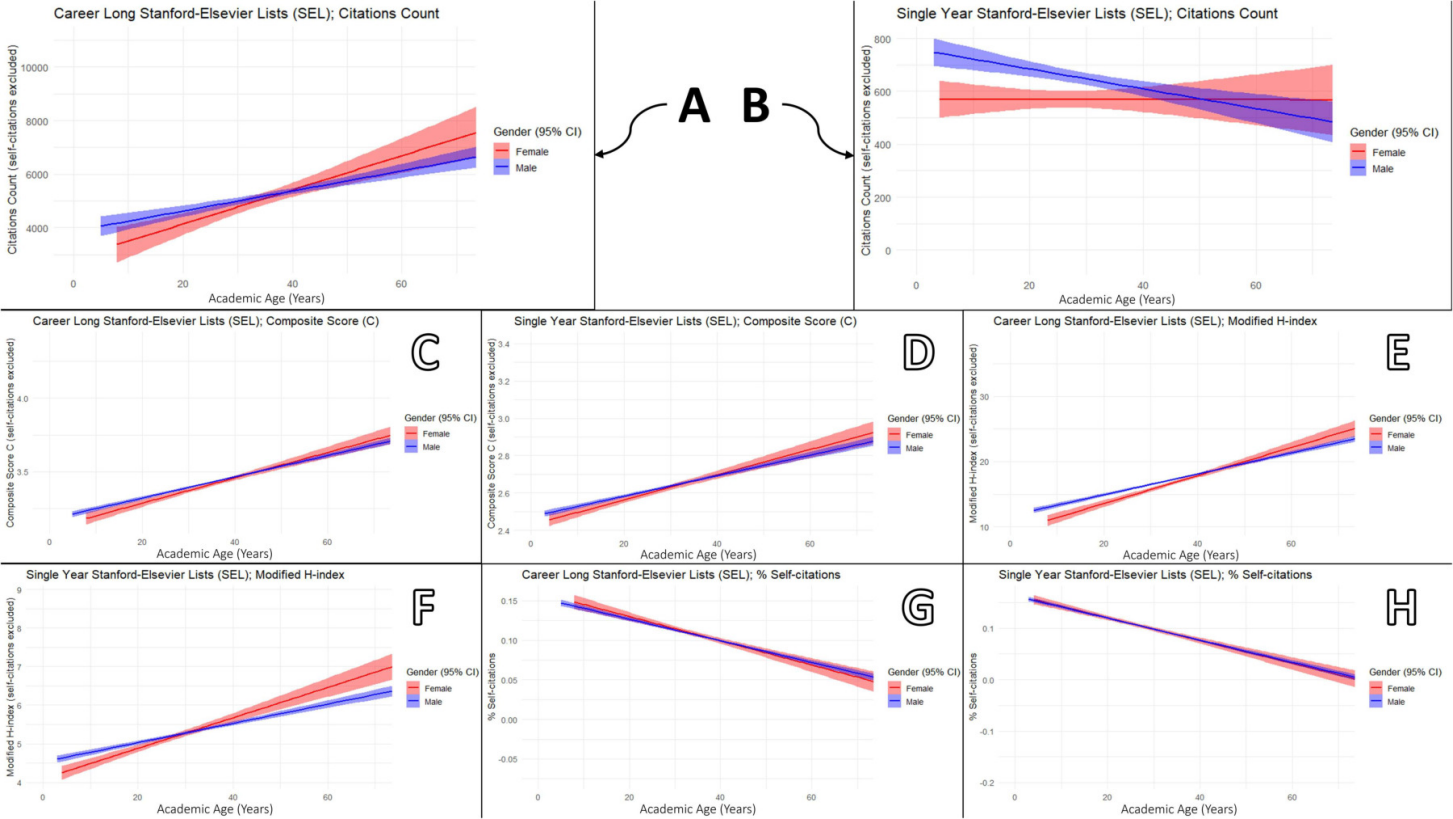
Gender-stratified associations between academic age and scholarly output metrics among excellent dental scholars (EDS) in the Stanford–Elsevier top 2% lists (2017–2023): **(A,B)** citation count, **(C,D)** composite score, **(E,F)** modified h-index, and **(G,H)** percentage of self-citations for *career-long* and *single-year* lists, respectively.

For individual-level indicators, male gender was significantly associated with a higher modified *h-*index in the *career-long* SEL [adjusted *β* = 0.55 (95% CI: 0.23–0.88)] and with higher citation counts in the *single-year* SEL [84.25 (34.08–134.43)]. Academic age demonstrated a more consistent effect across scholarly metrics: for each additional year of academic activity, the C-score increased (*career-long*: 0.01; *single-year*: 0.004), the modified *h-*index rose (0.18; 0.03), and the percentage of self-citations decreased slightly (–0.0011; −0.0019), all with statistically significant confidence intervals Figure 4.

Concerning national-level budgetary indicators, GDP expenditure on education was not significantly associated with any scholarly metric in either the *career-long* or *single-year* SEL. In the *career-long* SEL, higher GDP expenditure on health was associated with increased C-score [0.01 (0.006–0.018)], citation count [173.33 (52.81–293.85)], and reduced percentage of self-citations [–1.4 × 10^−^³ (–2.7 × 10^−^³ to −1.0 × 10^−^⁵)] Table 5.

Three sequential multivariable linear regression models were developed for each scholarly metric: Model 1 included individual-level indicators (gender and academic age); Model 2 added public health indicators (life expectancy and oral disease burden); and Model 3 further incorporated economic indicators (GNI per capita and GDP shares for R&D, health, and education). The progressive increase in explained variance (*R*²₃ > *R*²₂ > *R*²₁) aligns with the mixed-effects model findings, underscoring the effect of national-level indicators Supplementary Table 10.

## 4 Discussion

### 4.1 Summary of findings

The present analysis revealed that the distribution of EDS was strikingly uneven worldwide, with 80% of those listed in the *career-long* and *single-year* SEL were based in only 10 and 13 countries, respectively. Moreover, 96.1% and 88.9% of scholars in the *career-long* and *single-year* SEL were based in high-income countries. English-speaking countries accounted for 59.5% of *career-long* and 47.7% of *single-year* SEL, reflecting both the historical origins of dental sciences in Anglophone settings and the language bias inherent in Elsevier's journal indexing, which predominantly favours English-language publications (31–36). Institutional elitism was also evident, with 23.8% of *career-long* and 19.8% of *single-year* scholars affiliated with only 20 institutions, all of which are historically prestigious centres located in high-income countries. This concentration illustrates the continued clustering of resources and talent within long-established academic powerhouses such as the University of Washington, King's College London, and Harvard University, consistent with historical patterns of institutional dominance (37–39).

Macroeconomic indicators were positively correlated with number of EDS, particularly GDP share allocated to R&D (*career-long ρ* = 0.739; *single-year ρ* = 0.706), GDP per capita (0.604; 0.527), and GDP shares for health (0.538; 0.497) and education (0.462; 0.376). Gender disparities in dental research excellence were substantial, with women comprising only 14.8% of *career-long* and 18.1% of *single-year* SEL, while male scholars exhibited longer academic ages and higher scholarly output metrics. Mixed-effect regression models emphasised the principal role of World Bank income classification and official language as categorical determinants of scholarly output metrics. Academic age consistently emerged as a stronger predictor than gender across all scholarly performance indicators. In addition, national public health indicators, particularly life expectancy and the burden of oral diseases such as deciduous and permanent caries and edentulism, were also significantly associated with citation outcomes, suggesting that broader social and health system contexts are associated with research productivity, though causality cannot be inferred from these ecological associations.

### 4.2 Macroeconomic determinants of dental research excellence

The unequal distribution of research productivity in the biomedical sciences, including dentistry, is a well-documented, longstanding, and global phenomenon, predominantly attributed to macroeconomic factors (6, 40–44). A bibliometric analysis revealed that over half of dental research publications worldwide in 2013 originated from only five countries, i.e., the US, Brazil, India, Japan, and the UK (6). Moreover, the findings demonstrated strong positive correlations between dental research output and macroeconomic indicators such as HDI and GNI; countries in the highest HDI and GNI groups published on average 166.2 and 177.7 articles respectively, compared to only 1.7 and 0.8 articles in the lowest groups (6). Remarkably, certain middle-income economies, namely India and Brazil, exhibited disproportionately high research outputs, suggesting that strategic national priorities can significantly drive research productivity beyond macroeconomic determinants (45, 46).

Subnational disparities in dental research productivity are also evident, as shown by a recent bibliometric analysis of North East England (NEE), one of the most socioeconomically deprived regions in the UK, where all five NEE universities collectively contributed less than 4% of the country's dental publications, alongside low inter-institutional collaboration and uneven output distribution (47). In Brazil, a bibliometric analysis of dental research productivity across states revealed moderate correlations between the number of publications per 100,000 inhabitants and key socioeconomic indicators, including GNI per capita (*ρ* = 0.38), mean individual income (*ρ* = 0.40), and proportion of poverty (*ρ* = –0.48) (48). The subnational distribution of research output was strongly disproportionate, with the State of São Paulo alone accounting for 46% of all dental publications, while four states produced none during the study period (2006–2016) (48).

In contrast to research productivity, which is a predominantly quantitative metric, research excellence is a more qualitative construct that emphasises the potential impact of dental research on oral health outcomes and clinical practice (49–51). A recent study by Lalloo and Borrell analysed the distribution of recipients of the IADR annual awards from 2019 to 2024 and found that 94% were from high-income countries and 6% from upper-middle-income countries, with no recipients from lower-middle- or low-income countries, nor from the African or Middle Eastern regions (52). The US (38.8%), the UK (12.2%), and Australia (11.1%) accounted for the majority of awards, reflecting a dominance of both high-income and English-speaking countries in the global recognition of dental research excellence (52).

The present study identified consistent associations between dental research excellence and macroeconomic indicators, such as the GDP shares allocated to R&D and education. Empirical evidence suggests that higher efficiency of R&D investment, as measured by the number of scientific publications generated per 1% of GDP allocated to R&D, is not only indicative of enhanced research productivity but also significantly associated with long-term economic growth, particularly in emerging economies where the marginal returns appear more pronounced (53). Complementary findings from institutional-level analyses of US and European universities indicate that scholarly output and impact increase more than proportionally with the financial resources available to universities (54). In particular, funding per academic staff member emerged as a key driver of bibliometric performance, highlighting how concentrated investments enable institutions to attract talent and amplify research visibility (54).

Beyond macroeconomic indicators, structural barriers constrain the development of dental research excellence in low- and middle-income countries (LMIC) (55). Limited laboratory infrastructure, high costs of biomedical equipment, and disparities in remuneration discourage dentist-scientist careers and drive talent abroad. Although Africa bears a disproportionate share of the global disease burden, it receives only a fraction of global health research funding (56, 57). Additional non-economic barriers, including political instability, restricted access to scientific journals, and weak regional research networks, further limit capacity development and perpetuate inequities in global knowledge production (58, 59).

### 4.3 Institutional elitism in dental research excellence

The *Matthew effect* refers to the cumulative advantage by which well-resourced and visible institutions continue to attract disproportionate recognition, funding, and talent (60). To formally describe this persistent and historically documented phenomenon, particularly in academic medicine and dentistry, we propose the term *institutional elitism*, defined as “the systemic concentration of academic prestige, research productivity, and investment within a limited subset of institutions, reinforced by performance-based funding, reputation-driven rankings, and policy frameworks that favour scale and visibility” (54, 60–63).

The vicious circle underlying institutional elitism can be attributed to funding sustainability, as Katz and Matter found that since 1985, an increasing share of the US National Institutes of Health (NIH) funding has been captured by a small, fixed segment of scholars and institutions, with those initially in top funding tiers consistently retaining their positions, ultimately resulting in stasis and reduced academic mobility (63). Editorial bias is a contributing factor to institutional elitism, reflected in the preferential treatment received by authors affiliated with prestigious institutions, who benefit from both higher acceptance rates and shorter peer-review durations in leading academic journals (61). Another explanation for institutional elitism is the self-reinforcing nature of faculty hiring networks, in which a small number of prestigious institutions disproportionately place their graduates into academic positions across the system (64). This entrenched hierarchy not only perpetuates institutional dominance but also limits upward mobility and reinforces disparities in academic visibility, influence, and opportunity (64).

In terms of dental research productivity, institutional elitism is consistently observed; for example, in Brazil, more than half of all dental publications originate from only three institutions: the University of São Paulo (28.2%), São Paulo State University (14.7%), and the State University of Campinas (12.8%) (48). Likewise, in Spain, dental research between 1993 and 2012 was dominated by three institutions: the University of Granada (14.9%), the Complutense University of Madrid (13.2%), and the University of Valencia (10.3%) (65). Moreover, two Saudi institutions accounted for over half of the country's dental research output between 2009 and 2018, namely King Saud University (37.7%) and King Abdulaziz University (17.6%) (66).

The results of the present study largely echo previous findings from sporadic national analyses on the concentration of dental research production within historically prestigious institutions (48, 65, 66). They further demonstrate that dental research excellence, as measured by citation-based metrics, is similarly dominated by a narrow subset of institutions globally, with 20 institutions accounting for 23.8% of *career-long* and 19.8% of *single-year* scholars.

### 4.4 Gender disparities in dental research excellence

Dentistry, which originated as a predominantly male profession, has been undergoing a gradual feminisation globally, with women now comprising over 60% of practising dentists across Europe and nearly 80% in countries such as Finland, Russia, Latvia, and Lithuania (67–71). In the UK, this shift reached a milestone in 2021 when women accounted for 51% of all registered dentists; yet significant gender disparities persist: women represent only 22% of professors in academia, while they account for 58% of lecturers; they outnumber men in six of fifteen dental specialities, but remain markedly under-represented in oral surgery (less than one-third), prosthodontics (27%), and restorative dentistry (24%), highlighting a steep gender gradient in higher-status roles and specialties (71)..

Globally, gender disparities persist across various aspects of dental research and practice; for instance, an examination of editorial boards of dental journals indexed in the Journal Citation Reports® (Clarivate Analytics) revealed that 82% of editors-in-chief were men (72). Encouraging developments are emerging within leading dental organisations such as the World Dental Federation (FDI) and the IADR, which have adopted diversification policies aimed at promoting gender balance; at present, women comprise 76% of IADR and 84% of FDI headquarters staff, offering a potential model for national member organisations (73). Consequently, men account for 54% of chief dental officer positions, indicating a near-balanced distribution and signalling gradual progress towards gender parity in senior leadership roles (73).

A recent bibliometric analysis of the most-cited dental publications from 1980 to 2019 revealed a pronounced gender imbalance, with men accounting for 83.8% of first authors and 86.8% of last authors (74). Although women's representation as last authors increased modestly from 6% in the 1980s to 22% in the 2010s, no significant progress was observed in their representation as first authors (74). Likewise, another bibliometric analysis of dental publications between 1996 and 2019 revealed that women accounted for 28.4% of first authors and 22.1% of last authors, with a modest upward trend in last authorship from 16.1% in 1996 to 22.1% in 2019 (75).

On the other side of the coin, women scholars appear to lead dental research output in low-income settings (76). For instance, in Nigeria, women scholars had significantly more Web of Science-indexed publications (3.7 vs. 2.6; *p* = 0.03), received more citations (3,892 vs. 3,779; *p* = 0.04), and held a greater share of first-authorship positions (26.6% vs. 20.5%; *p* = 0.048) compared to their male counterparts (76). Moreover, between 2016 and 2023, four women scholars were ranked among the top ten most productive authors in Africa in terms of dental research output (7).

In the US, women constituted only 36.5% of dental faculty and just 24.4% of full professors at the top eight NIH-funded dental schools in 2017 (77). Women dental faculty tend to be younger, as they have generally graduated more recently (77, 78). Although women faculty had fewer publications and lower *h-*indices than men, they had graduated more recently, and when adjusted for age and productivity, gender was not significantly associated with academic rank (77). Furthermore, between 2007 and 2016, approximately two-thirds of NIH Research Project Grant applicants and awardees in dental and oral health research were men (79). Although men submitted more applications and received more awards, no gender differences were observed in award rates or in the age at which the first early-stage investigator grant (R01) was obtained (79). These results reflect historic gender disparities reflected in senior dental academic positions distribution, yet suggest that such imbalances may be gradually diminishing as increasing numbers of female dental graduates enter academia and research, where they increasingly match or surpass their male counterparts in early-career performance.

In the present study, women were significantly underrepresented among EDS globally, constituting only 14.8% of the *career-long* SEL and 18.1% of the *single-year* SEL, thus reflecting the historic gender imbalance in dentistry. Nevertheless, female representation across SEL annual updates from 2017 to 2023 demonstrated a clear upward trend, and women scholars were significantly younger than their male counterparts. Notably, regression analyses adjusted for academic age and macroeconomic indicators revealed no significant gender differences in scholarly output metrics, suggesting comparable research influence between male and female scholars.

### 4.5 Academic age and dental research excellence

The association between academic age and research productivity in dentistry has been recognised for decades, predating the widespread adoption of common author-level scientometrics, such as the *h-*index (80). In contrast to intuitive assumptions of linear growth, the relationship between academic age and citation-based metrics exhibits a non-linear pattern, characterised by phases of initial increase, subsequent stabilisation, and eventual decline (81, 82). Milestones in academic careers, such as the attainment of a PhD, can serve as important catalysts for subsequent research performance, with empirical evidence suggesting that younger PhD graduates tend to achieve higher productivity and citation impact over time (83). Nevertheless, there remains a lack of in-depth analyses focusing on dental scholars to determine whether similar milestones contribute to catalysing and sustaining long-term research productivity or excellence.

The findings of this study demonstrated that academic age was the only factor significantly associated with all scholarly output metrics, i.e., citation count, *h*-index, C-score, and the proportion of self-citations, in both unadjusted and adjusted regression models, confirming its predictive value for dental research excellence. However, further research is needed to explore the underlying mechanisms and trajectories of this factor within the context of dental research.

### 4.6 Oral diseases burden and dental research excellence

Disease burden is an appealing contextual variable in bibliometric studies, as it links research activity to population health needs. A recent bibliometric analysis of surgical publications from 2010 to 2022 revealed a weak and statistically non-significant correlation between surgical disease burden and both research output (*ρ* = –0.041, *p* = 0.682) and research-producing human capital (*ρ* = –0.047, *p* = 0.641) (84). Furthermore, more than 90% of the global surgical disease burden was concentrated in countries outside the top 20 contributors to surgical research, underscoring a substantial misalignment between research efforts and global health priorities (84). Likewise, a bibliometric analysis of oncology research in Southeast Asia (SEA) between 1980 and 2020 revealed a significant inverse relationship between disease burden and research productivity, with higher incidence, mortality, and DALYs associated with lower levels of key bibliometric indicators, including total publications, citations, and social media attention (85). Furthermore, dementia research production in SEA was not significantly associated with disease burden in any of the region's countries (86).

Funding allocation for health research may explain observed mismatches between research activity and disease burden in different disciplines. A bibliometric analysis of 52 infectious diseases showed that while HIV/AIDS and influenza attracted disproportionately high research attention, many neglected tropical diseases such as paratyphoid fever and schistosomiasis remained under-researched relative to their burden, as indicated by the Burden Adjusted Research Intensity (BARI) index (87). Oral diseases emphasise this mismatch, as shown by a recent Australian study that assessed government research funding in relation to disease burden (88). Although oral disorders accounted for a substantial proportion of non-fatal DALYs, they received only 15 million AUD in NHMRC funding between 2017 and 2021, representing just 0.23% of the total NHMRC budget allocated to the top 75 disease categories in Australia. This resulted in a Fair Research Funding (FRF) index of 10.7, the highest level of underfunding among all categories assessed (88).

In spite of oral diseases constituting the largest share of the non-fatal disease burden across all WHO regions and countries, the present study identified a negative correlation between this burden and both the number of EDS per country and their citation count, with deciduous caries showing *ρ* = −0.527 and −0.536, and permanent caries showing *ρ* = −0.375 and −0.386, respectively (89).

### 4.7 Time-trends of dental research excellence

The inclusion of both *career-long* and *single-year* SEL aimed to capture temporal patterns in dental research excellence. While the *career-long* SEL provides a cumulative and historical perspective, the *single-year* SEL offers a more current, cross-sectional view of ongoing shifts. Complementing this approach, annual comparisons of SEL updates from 2017 to 2023 revealed five major trends that suggest a progressively inclusive and evolving global dental research landscape.

Firstly, increasing female representation, with women comprising a higher proportion of the *single-year* SEL (18.1%) compared to the *career-long* SEL (14.8%). This trend is further supported by the rise in female inclusion from 10.9% (*career-long*) and 15.7% (*single-year*) in 2017 to 16.6% and 20.6%, respectively, in 2023. This finding echoes the global trend of feminisation in dental education, practice, and research (67–71)..

Secondly, increasing geographical diversity, with more countries represented in the *single-year* SEL (*n* = 71) compared to the *career-long* SEL (*n* = 65), including a greater presence of Global South countries in the former, e.g., Bangladesh, Cambodia, and Vietnam. Annual SEL comparisons also revealed an upward trend in the number of represented countries, increasing from 33 to 57 in the *career-long* SEL and from 37 to 63 in the *single-year* SEL between 2017 and 2023. This finding aligns with previous observations of increased dental research productivity in countries that have historically shown limited engagement in dental research (8).

Thirdly, increasing the representation of non-high-income countries, with their proportion being higher in the *single-year* SEL (11.1%) than the *career-long* SEL (3.9%), and showing an upward trend between 2017 (1.0% and 4.5%) and 2023 (5.5% and 13.8%), respectively. It is worth noting that this rise is largely driven by upper-middle-income countries, particularly Brazil and China, with only minimal and stagnating contributions from lower-middle and low-income countries.

Fourthly, increasing non-English-speaking countries representation, with their proportion being higher in the *single-year* SEL (52.3%) than the *career-long* SEL (41.5%), and showing an upward trend between 2017 (33.7% and 39.5%) and 2023 (43% and 55.6%), respectively. Given that the vast majority of Elsevier-indexed publications are in English, this trend reflects the growing engagement of scholars based in non-English-speaking countries in publishing their work in English as the lingua franca of science (90, 91).

Fifthly, decreasing median age of enlisted EDS was observed, with women in the *single-year* SEL having a lower median age compared to those in the *career-long* SEL (26 vs. 35 years), and men showing a similar pattern (31 vs. 38 years). Recently, empirical evidence indicated that pursuing an academic career was cited as the first or second career preference by 3.6% and 13.5%, respectively, of a global sample of dental students (92). This reflects a growing interest among young dentists in engaging with research.

### 4.8 Implications

The concentration of EDS in high-income countries reflects persistent global disparities in research capacity and suggests a potential role for sustained investment in research infrastructure and academic training programmes within low- and middle-income settings. The dominance of a small number of elite institutions in global dental research reflects systemic institutional elitism, which underscores the importance of diversifying funding allocation and enhancing visibility of emerging research centres. The pronounced underrepresentation of women among EDS, despite comparable citation outcomes, calls for institutional reforms that promote gender equity through supportive academic pathways and inclusive leadership development. Finally, the negative correlation between oral disease burden and scholarly excellence signals a misalignment between research outputs and population health needs, highlighting the urgency of reorienting national research priorities toward high-burden conditions.

### 4.9 Limitations

One limitation of this study is the reliance on citation-based metrics, which may favour senior scholars and underrepresent regionally relevant or non-English outputs (93). The SEL methodology partly mitigates these concerns by excluding self-citations, adjusting for authorship position, and employing a composite citation score that better reflects individual contributions. Complementary dimensions of excellence, including translational value, societal outcomes, and clinical relevance, were not captured by the SEL framework and should be considered in future investigations for a more comprehensive assessment.

Another limitation is gender assignment, an inherent challenge in bibliometric research. Algorithmic inference is vulnerable to cultural variation and inconsistent naming conventions, leading to potential misclassification. Such errors are more likely to attenuate observed gender differences than to generate spurious disparities, rendering estimates conservative. Greater transparency and standardisation in gender determination, together with integration of self-reported demographic data where feasible, remain essential for future research (94, 95).

Additionally, the use of academic age as a proxy for biological age, a practice that is criticised as the correlation between the two is not universal; empirical evidence suggests that the use of academic age should be limited to science, technology, engineering, mathematics, and medicine (STEMM) disciplines and scientifically advanced countries (96).

### 4.10 Strengths

This study is the first to focus specifically on dental research excellence, rather than productivity, offering a qualitatively distinct perspective on scholarly impact. It represents the most extensive analysis to date, utilising both the *career-long* and *single-year* SEL over a seven-year period (2017–2023) to enable cumulative and time-trend assessments. Moreover, by incorporating national-, institutional-, and individual-level determinants, the study provides a multidimensional framework for understanding the macroeconomic and sociodemographic drivers of dental research excellence. Finally, the use of mixed-effects linear regression models with country-level clustering and contextual fixed effects enhances the robustness of the findings by accounting for unobserved heterogeneity and structural confounding.

## 5 Conclusion

Excellence in dental research, as measured by citation-based indicators, is not evenly distributed worldwide but shaped by national-, institutional-, and individual-level factors. This study found that high-income countries and a select group of elite institutions dominate the global landscape of dental research excellence, while lower-income regions remain markedly underrepresented. Gender disparities persist, with female scholars comprising a minority of EDS, although their citation performance was comparable to that of male peers when adjusted for academic age. Notably, academic age proved to be a consistent predictor of scholarly output across all metrics. The negative correlation between oral disease burden and the presence of EDS points to a misalignment between research impact and public health relevance. These findings highlight disparities in research excellence and point to the need for more inclusive research policies that strengthen capacity in underserved regions and promote equity in academic recognition systems.

## Data Availability

Publicly available datasets were analyzed in this study. This data can be found here: Elsevier data repository; version 1 (July 2019) doi: 10.17632/btchxktzyw.1; Version 2 (October 2020) doi: 10.17632/btchxktzyw.2; version 3 (October 2021) doi: 10.17632/btchxktzyw.3; version 4 (October 2022) doi: 10.17632/btchxktzyw.4; version 5 (November 2022) doi: 10.17632/btchxktzyw.5; version 6 (October 2023) doi: 10.17632/btchxktzyw.6; version 7 (September 2024) doi: 10.17632/btchxktzyw.7.
