# Supplementary material for: National-, institutional-, and individual-level determinants of dental research excellence: an analysis of Stanford–Elsevier lists of the top 2% scholars worldwide (2017–2023)
Source: Front Oral Health. 2025 Oct 3;6:1675102. doi: 10.3389/froh.2025.1675102 (PMC12531218; doi:10.3389/froh.2025.1675102)
Supplement: Supplementary file 1 [file Table1.docx]

**Table S1.** National-level Analysis: Distribution of Dental Scholars in the *Career-Long* Stanford-Elsevier Lists (SEL) of Top 2% Scientists Worldwide (2017–2023)

| **Rank** | **Country** | **SEL 2017** | **SEL 2018** | **SEL 2019** | **SEL 2020** | **SEL 2021** | **SEL 2022** | **SEL 2023** | **Total ▼** | **Σ%** |
| --- | --- | --- | --- | --- | --- | --- | --- | --- | --- | --- |
| **1** | United States | 314 (44.3%) | 346 (46.2%) | 582 (41.1%) | 703 (40.1%) | 760 (38.9%) | 797 (38.6%) | 850 (38.5%) | 4352 (40.1%) | 40.1% |
| **2** | United Kingdom | 90 (12.7%) | 89 (11.9%) | 182 (12.9%) | 213 (12.2%) | 232 (11.9%) | 243 (11.8%) | 251 (11.4%) | 1300 (12.0%) | 52.1% |
| **3** | Sweden | 40 (5.6%) | 48 (6.4%) | 91 (6.4%) | 105 (6.0%) | 117 (6.0%) | 121 (5.9%) | 126 (5.7%) | 648 (6.0%) | 58.1% |
| **4** | Canada | 38 (5.4%) | 33 (4.4%) | 58 (4.1%) | 64 (3.7%) | 72 (3.7%) | 71 (3.4%) | 77 (3.5%) | 413 (3.8%) | 61.9% |
| **5** | Japan | 18 (2.5%) | 17 (2.3%) | 45 (3.2%) | 70 (4.0%) | 75 (3.8%) | 82 (4.0%) | 83 (3.8%) | 390 (3.6%) | 65.5% |
| **6** | Germany | 15 (2.1%) | 19 (2.5%) | 42 (3.0%) | 62 (3.5%) | 76 (3.9%) | 81 (3.9%) | 87 (3.9%) | 382 (3.5%) | 69.0% |
| **7** | Switzerland | 32 (4.5%) | 28 (3.7%) | 49 (3.5%) | 54 (3.1%) | 63 (3.2%) | 68 (3.3%) | 69 (3.1%) | 363 (3.3%) | 72.3% |
| **8** | Netherlands | 20 (2.8%) | 22 (2.9%) | 45 (3.2%) | 62 (3.5%) | 62 (3.2%) | 65 (3.1%) | 70 (3.2%) | 346 (3.2%) | 75.5% |
| **9** | Denmark | 27 (3.8%) | 22 (2.9%) | 33 (2.3%) | 46 (2.6%) | 52 (2.7%) | 51 (2.5%) | 51 (2.3%) | 282 (2.6%) | 78.1% |
| **10** | Italy | 8 (1.1%) | 12 (1.6%) | 33 (2.3%) | 45 (2.6%) | 49 (2.5%) | 60 (2.9%) | 73 (3.3%) | 280 (2.6%) | 80.7% |
| **11** | Australia | 23 (3.2%) | 23 (3.1%) | 37 (2.6%) | 40 (2.3%) | 48 (2.5%) | 51 (2.5%) | 55 (2.5%) | 277 (2.6%) | 83.3% |
| **12** | Finland | 16 (2.3%) | 15 (2.0%) | 27 (1.9%) | 35 (2.0%) | 40 (2.0%) | 40 (1.9%) | 43 (2.0%) | 216 (2.0%) | 85.3% |
| **13** | Norway | 10 (1.4%) | 12 (1.6%) | 23 (1.6%) | 36 (2.1%) | 41 (2.1%) | 38 (1.8%) | 38 (1.7%) | 198 (1.8%) | 87.1% |
| **14** | Brazil | 2 (0.3%) | 6 (0.8%) | 22 (1.6%) | 27 (1.5%) | 35 (1.8%) | 37 (1.8%) | 48 (2.2%) | 177 (1.6%) | 88.7% |
| **15** | Israel | 11 (1.6%) | 8 (1.1%) | 16 (1.1%) | 24 (1.4%) | 29 (1.5%) | 33 (1.6%) | 32 (1.5%) | 153 (1.4%) | 90.1% |
| **16** | Belgium | 11 (1.6%) | 8 (1.1%) | 17 (1.2%) | 21 (1.2%) | 22 (1.1%) | 22 (1.1%) | 25 (1.1%) | 126 (1.2%) | 91.3% |
| **17** | Hong Kong | 6 (0.8%) | 7 (0.9%) | 11 (0.8%) | 16 (0.9%) | 19 (1.0%) | 17 (0.8%) | 18 (0.8%) | 94 (0.9%) | 92.1% |
| **18** | Spain | 2 (0.3%) | 3 (0.4%) | 12 (0.8%) | 14 (0.8%) | 17 (0.9%) | 19 (0.9%) | 22 (1.0%) | 89 (0.8%) | 93.0% |
| **19** | France | 5 (0.7%) | 5 (0.7%) | 11 (0.8%) | 7 (0.4%) | 12 (0.6%) | 14 (0.7%) | 15 (0.7%) | 69 (0.6%) | 93.6% |
| **20** | South Korea | 1 (0.1%) | 2 (0.3%) | 9 (0.6%) | 11 (0.6%) | 13 (0.7%) | 16 (0.8%) | 15 (0.7%) | 67 (0.6%) | 94.2% |
| **21** | China | 1 (0.1%) | 1 (0.1%) | 5 (0.4%) | 8 (0.5%) | 12 (0.6%) | 16 (0.8%) | 17 (0.8%) | 60 (0.6%) | 94.8% |
| **22** | Taiwan | 2 (0.3%) | 3 (0.4%) | 7 (0.5%) | 8 (0.5%) | 13 (0.7%) | 12 (0.6%) | 13 (0.6%) | 58 (0.5%) | 95.3% |
| **23** | New Zealand | 3 (0.4%) | 3 (0.4%) | 7 (0.5%) | 8 (0.5%) | 9 (0.5%) | 8 (0.4%) | 9 (0.4%) | 47 (0.4%) | 95.7% |
| **24** | Austria | 1 (0.1%) | 2 (0.3%) | 8 (0.6%) | 7 (0.4%) | 9 (0.5%) | 8 (0.4%) | 9 (0.4%) | 44 (0.4%) | 96.1% |
| **25** | Greece | 1 (0.1%) | 0 (0.0%) | 5 (0.4%) | 9 (0.5%) | 8 (0.4%) | 9 (0.4%) | 10 (0.5%) | 42 (0.4%) | 96.5% |
| **=25** | Ireland | 3 (0.4%) | 1 (0.1%) | 5 (0.4%) | 6 (0.3%) | 7 (0.4%) | 9 (0.4%) | 11 (0.5%) | 42 (0.4%) | 96.9% |
| **26** | United Arab Emirates | 0 (0.0%) | 3 (0.4%) | 7 (0.5%) | 6 (0.3%) | 6 (0.3%) | 7 (0.3%) | 7 (0.3%) | 36 (0.3%) | 97.3% |
| **27** | Turkey | 1 (0.1%) | 1 (0.1%) | 1 (0.1%) | 6 (0.3%) | 8 (0.4%) | 8 (0.4%) | 10 (0.5%) | 35 (0.3%) | 97.6% |
| **28** | South Africa | 2 (0.3%) | 2 (0.3%) | 4 (0.3%) | 2 (0.1%) | 5 (0.3%) | 6 (0.3%) | 6 (0.3%) | 27 (0.2%) | 97.8% |
| **29** | Iran | 0 (0.0%) | 0 (0.0%) | 3 (0.2%) | 4 (0.2%) | 4 (0.2%) | 7 (0.3%) | 8 (0.4%) | 26 (0.2%) | 98.1% |
| **30** | India | 1 (0.1%) | 0 (0.0%) | 0 (0.0%) | 2 (0.1%) | 6 (0.3%) | 5 (0.2%) | 9 (0.4%) | 23 (0.2%) | 98.3% |
| **31** | Singapore | 0 (0.0%) | 4 (0.5%) | 3 (0.2%) | 3 (0.2%) | 3 (0.2%) | 2 (0.1%) | 5 (0.2%) | 20 (0.2%) | 98.5% |
| **32** | Egypt | 0 (0.0%) | 0 (0.0%) | 1 (0.1%) | 2 (0.1%) | 2 (0.1%) | 4 (0.2%) | 5 (0.2%) | 14 (0.1%) | 98.6% |
| **33** | Qatar | 0 (0.0%) | 0 (0.0%) | 1 (0.1%) | 3 (0.2%) | 3 (0.2%) | 3 (0.1%) | 3 (0.1%) | 13 (0.1%) | 98.7% |
| **=33** | Saudi Arabia | 0 (0.0%) | 0 (0.0%) | 1 (0.1%) | 2 (0.1%) | 2 (0.1%) | 3 (0.1%) | 5 (0.2%) | 13 (0.1%) | 98.8% |
| **34** | Hungary | 0 (0.0%) | 0 (0.0%) | 0 (0.0%) | 3 (0.2%) | 2 (0.1%) | 3 (0.1%) | 3 (0.1%) | 11 (0.1%) | 98.9% |
| **35** | Nigeria | 0 (0.0%) | 0 (0.0%) | 0 (0.0%) | 1 (0.1%) | 3 (0.2%) | 3 (0.1%) | 3 (0.1%) | 10 (0.1%) | 99.0% |
| **36** | Mexico | 0 (0.0%) | 0 (0.0%) | 2 (0.1%) | 2 (0.1%) | 1 (0.1%) | 2 (0.1%) | 1 (0.0%) | 8 (0.1%) | 99.1% |
| **37** | Jordan | 0 (0.0%) | 0 (0.0%) | 1 (0.1%) | 1 (0.1%) | 1 (0.1%) | 1 (0.0%) | 3 (0.1%) | 7 (0.1%) | 99.2% |
| **=37** | Malaysia | 0 (0.0%) | 0 (0.0%) | 0 (0.0%) | 0 (0.0%) | 1 (0.1%) | 3 (0.1%) | 3 (0.1%) | 7 (0.1%) | 99.2% |
| **=37** | Portugal | 0 (0.0%) | 0 (0.0%) | 1 (0.1%) | 1 (0.1%) | 2 (0.1%) | 2 (0.1%) | 1 (0.0%) | 7 (0.1%) | 99.3% |
| **38** | Kuwait | 3 (0.4%) | 2 (0.3%) | 0 (0.0%) | 1 (0.1%) | 0 (0.0%) | 0 (0.0%) | 0 (0.0%) | 6 (0.1%) | 99.3% |
| **=38** | Liechtenstein | 0 (0.0%) | 1 (0.1%) | 1 (0.1%) | 1 (0.1%) | 1 (0.1%) | 1 (0.0%) | 1 (0.0%) | 6 (0.1%) | 99.4% |
| **=38** | Thailand | 0 (0.0%) | 1 (0.1%) | 1 (0.1%) | 1 (0.1%) | 1 (0.1%) | 1 (0.0%) | 1 (0.0%) | 6 (0.1%) | 99.5% |
| **39** | Chile | 0 (0.0%) | 0 (0.0%) | 1 (0.1%) | 1 (0.1%) | 1 (0.1%) | 1 (0.0%) | 1 (0.0%) | 5 (0.0%) | 99.5% |
| **=39** | Iceland | 0 (0.0%) | 0 (0.0%) | 1 (0.1%) | 1 (0.1%) | 0 (0.0%) | 1 (0.0%) | 2 (0.1%) | 5 (0.0%) | 99.5% |
| **=39** | Slovenia | 0 (0.0%) | 0 (0.0%) | 1 (0.1%) | 1 (0.1%) | 1 (0.1%) | 1 (0.0%) | 1 (0.0%) | 5 (0.0%) | 99.6% |
| **40** | Croatia | 0 (0.0%) | 0 (0.0%) | 0 (0.0%) | 1 (0.1%) | 1 (0.1%) | 1 (0.0%) | 1 (0.0%) | 4 (0.0%) | 99.6% |
| **=40** | Lebanon | 0 (0.0%) | 0 (0.0%) | 0 (0.0%) | 1 (0.1%) | 1 (0.1%) | 1 (0.0%) | 1 (0.0%) | 4 (0.0%) | 99.7% |
| **=40** | Russia | 0 (0.0%) | 0 (0.0%) | 0 (0.0%) | 0 (0.0%) | 0 (0.0%) | 3 (0.1%) | 1 (0.0%) | 4 (0.0%) | 99.7% |
| **=40** | Zimbabwe | 0 (0.0%) | 0 (0.0%) | 0 (0.0%) | 1 (0.1%) | 1 (0.1%) | 1 (0.0%) | 1 (0.0%) | 4 (0.0%) | 99.7% |
| **41** | Argentina | 0 (0.0%) | 0 (0.0%) | 0 (0.0%) | 0 (0.0%) | 1 (0.1%) | 1 (0.0%) | 1 (0.0%) | 3 (0.0%) | 99.8% |
| **=41** | Cyprus | 0 (0.0%) | 0 (0.0%) | 0 (0.0%) | 0 (0.0%) | 1 (0.1%) | 1 (0.0%) | 1 (0.0%) | 3 (0.0%) | 99.8% |
| **=41** | Pakistan | 0 (0.0%) | 0 (0.0%) | 0 (0.0%) | 0 (0.0%) | 1 (0.1%) | 1 (0.0%) | 1 (0.0%) | 3 (0.0%) | 99.8% |
| **=41** | Poland | 0 (0.0%) | 0 (0.0%) | 0 (0.0%) | 1 (0.1%) | 1 (0.1%) | 1 (0.0%) | 0 (0.0%) | 3 (0.0%) | 99.9% |
| **=41** | Serbia | 0 (0.0%) | 0 (0.0%) | 0 (0.0%) | 0 (0.0%) | 1 (0.1%) | 1 (0.0%) | 1 (0.0%) | 3 (0.0%) | 99.9% |
| **42** | Bulgaria | 0 (0.0%) | 0 (0.0%) | 0 (0.0%) | 0 (0.0%) | 0 (0.0%) | 1 (0.0%) | 1 (0.0%) | 2 (0.0%) | 99.9% |
| **=42** | Oman | 0 (0.0%) | 0 (0.0%) | 0 (0.0%) | 1 (0.1%) | 1 (0.1%) | 0 (0.0%) | 0 (0.0%) | 2 (0.0%) | 99.9% |
| **=42** | Romania | 0 (0.0%) | 0 (0.0%) | 1 (0.1%) | 1 (0.1%) | 0 (0.0%) | 0 (0.0%) | 0 (0.0%) | 2 (0.0%) | 99.9% |
| **=42** | Yemen | 0 (0.0%) | 0 (0.0%) | 0 (0.0%) | 0 (0.0%) | 0 (0.0%) | 1 (0.0%) | 1 (0.0%) | 2 (0.0%) | 100.0% |
| **43** | Georgia | 0 (0.0%) | 0 (0.0%) | 1 (0.1%) | 0 (0.0%) | 0 (0.0%) | 0 (0.0%) | 0 (0.0%) | 1 (0.0%) | 100.0% |
| **=43** | Indonesia | 0 (0.0%) | 0 (0.0%) | 1 (0.1%) | 0 (0.0%) | 0 (0.0%) | 0 (0.0%) | 0 (0.0%) | 1 (0.0%) | 100.0% |
| **=43** | Laos | 0 (0.0%) | 0 (0.0%) | 0 (0.0%) | 0 (0.0%) | 0 (0.0%) | 1 (0.0%) | 0 (0.0%) | 1 (0.0%) | 100.0% |
| **=43** | Malta | 1 (0.1%) | 0 (0.0%) | 0 (0.0%) | 0 (0.0%) | 0 (0.0%) | 0 (0.0%) | 0 (0.0%) | 1 (0.0%) | 100.0% |
| **=43** | Puerto Rico | 1 (0.1%) | 0 (0.0%) | 0 (0.0%) | 0 (0.0%) | 0 (0.0%) | 0 (0.0%) | 0 (0.0%) | 1 (0.0%) | 100.0% |
| *NA* | *Missed* | 94 (11.7%) | 2 (0.3%) | 8 (0.6%) | 24 (1.4%) | 15 (0.8%) | 18 (0.9%) | 13 (0.6%) | 174 (1.6%) |  |
|  | **Total** | 803 | 751 | 1423 | 1775 | 1969 | 2084 | 2218 | 11023 |  |

**Table S2.** National-level Analysis: Distribution of Dental Scholars in the *Single-Year* Stanford-Elsevier Lists (SEL) of Top 2% Scientists Worldwide (2017–2023)

| **Rank** | **Country** | **SEL 2017** | **SEL 2019** | **SEL 2020** | **SEL 2021** | **SEL 2022** | **SEL 2023** | **Total ▼** | **Σ%** |
| --- | --- | --- | --- | --- | --- | --- | --- | --- | --- |
| **1** | United States | 214 (38.3%) | 481 (34.2%) | 591 (32.5%) | 619 (30.7%) | 629 (29.6%) | 657 (29.1%) | 3191 (31.3%) | 31.3% |
| **2** | United Kingdom | 75 (13.4%) | 141 (10.0%) | 178 (9.8%) | 181 (9.0%) | 197 (9.3%) | 189 (8.4%) | 961 (9.4%) | 40.8% |
| **3** | Italy | 20 (3.6%) | 91 (6.5%) | 114 (6.3%) | 142 (7.1%) | 153 (7.2%) | 160 (7.1%) | 680 (6.7%) | 47.5% |
| **4** | Germany | 23 (4.1%) | 72 (5.1%) | 96 (5.3%) | 106 (5.3%) | 120 (5.6%) | 119 (5.3%) | 536 (5.3%) | 52.7% |
| **5** | Brazil | 12 (2.1%) | 58 (4.1%) | 75 (4.1%) | 94 (4.7%) | 102 (4.8%) | 103 (4.6%) | 444 (4.4%) | 57.1% |
| **6** | Switzerland | 35 (6.3%) | 61 (4.3%) | 69 (3.8%) | 70 (3.5%) | 75 (3.5%) | 77 (3.4%) | 387 (3.8%) | 60.9% |
| **7** | Sweden | 23 (4.1%) | 64 (4.5%) | 71 (3.9%) | 64 (3.2%) | 65 (3.1%) | 59 (2.6%) | 346 (3.4%) | 64.3% |
| **8** | Japan | 10 (1.8%) | 47 (3.3%) | 59 (3.2%) | 72 (3.6%) | 75 (3.5%) | 81 (3.6%) | 344 (3.4%) | 67.7% |
| **9** | Netherlands | 16 (2.9%) | 45 (3.2%) | 54 (3.0%) | 59 (2.9%) | 61 (2.9%) | 67 (3.0%) | 302 (3.0%) | 70.6% |
| **10** | Canada | 25 (4.5%) | 47 (3.3%) | 54 (3.0%) | 56 (2.8%) | 55 (2.6%) | 64 (2.8%) | 301 (3.0%) | 73.6% |
| **11** | Australia | 19 (3.4%) | 38 (2.7%) | 50 (2.7%) | 59 (2.9%) | 61 (2.9%) | 60 (2.7%) | 287 (2.8%) | 76.4% |
| **12** | China | 4 (0.7%) | 18 (1.3%) | 38 (2.1%) | 49 (2.4%) | 70 (3.3%) | 94 (4.2%) | 273 (2.7%) | 79.1% |
| **13** | Denmark | 17 (3.0%) | 28 (2.0%) | 39 (2.1%) | 39 (1.9%) | 33 (1.6%) | 34 (1.5%) | 190 (1.9%) | 81.0% |
| **14** | South Korea | 4 (0.7%) | 19 (1.4%) | 26 (1.4%) | 31 (1.5%) | 44 (2.1%) | 48 (2.1%) | 172 (1.7%) | 82.7% |
| **15** | Spain | 2 (0.4%) | 18 (1.3%) | 30 (1.6%) | 40 (2.0%) | 35 (1.6%) | 43 (1.9%) | 168 (1.7%) | 84.3% |
| **16** | Belgium | 12 (2.1%) | 20 (1.4%) | 22 (1.2%) | 29 (1.4%) | 27 (1.3%) | 31 (1.4%) | 141 (1.4%) | 85.7% |
| **17** | Hong Kong | 6 (1.1%) | 21 (1.5%) | 22 (1.2%) | 22 (1.1%) | 19 (0.9%) | 22 (1.0%) | 112 (1.1%) | 86.8% |
| **18** | Finland | 8 (1.4%) | 13 (0.9%) | 19 (1.0%) | 23 (1.1%) | 19 (0.9%) | 23 (1.0%) | 105 (1.0%) | 87.8% |
| **19** | Israel | 6 (1.1%) | 10 (0.7%) | 20 (1.1%) | 21 (1.0%) | 24 (1.1%) | 22 (1.0%) | 103 (1.0%) | 88.8% |
| **20** | India | 0 (0.0%) | 5 (0.4%) | 14 (0.8%) | 25 (1.2%) | 24 (1.1%) | 28 (1.2%) | 96 (0.9%) | 89.8% |
| **=20** | Norway | 4 (0.7%) | 13 (0.9%) | 19 (1.0%) | 24 (1.2%) | 19 (0.9%) | 17 (0.8%) | 96 (0.9%) | 90.7% |
| **=20** | Saudi Arabia | 0 (0.0%) | 7 (0.5%) | 14 (0.8%) | 20 (1.0%) | 26 (1.2%) | 29 (1.3%) | 96 (0.9%) | 91.7% |
| **21** | Iran | 2 (0.4%) | 9 (0.6%) | 14 (0.8%) | 13 (0.6%) | 22 (1.0%) | 26 (1.2%) | 86 (0.8%) | 92.5% |
| **=21** | Taiwan | 2 (0.4%) | 15 (1.1%) | 18 (1.0%) | 17 (0.8%) | 15 (0.7%) | 19 (0.8%) | 86 (0.8%) | 93.3% |
| **22** | France | 2 (0.4%) | 6 (0.4%) | 8 (0.4%) | 17 (0.8%) | 16 (0.8%) | 20 (0.9%) | 69 (0.7%) | 94.0% |
| **23** | Turkey | 1 (0.2%) | 5 (0.4%) | 12 (0.7%) | 12 (0.6%) | 13 (0.6%) | 13 (0.6%) | 56 (0.6%) | 94.6% |
| **24** | Greece | 1 (0.2%) | 2 (0.1%) | 11 (0.6%) | 12 (0.6%) | 12 (0.6%) | 14 (0.6%) | 52 (0.5%) | 95.1% |
| **25** | New Zealand | 3 (0.5%) | 7 (0.5%) | 9 (0.5%) | 10 (0.5%) | 10 (0.5%) | 11 (0.5%) | 50 (0.5%) | 95.6% |
| **26** | Egypt | 2 (0.4%) | 4 (0.3%) | 7 (0.4%) | 7 (0.3%) | 10 (0.5%) | 11 (0.5%) | 41 (0.4%) | 96.0% |
| **27** | Singapore | 0 (0.0%) | 5 (0.4%) | 7 (0.4%) | 7 (0.3%) | 7 (0.3%) | 14 (0.6%) | 40 (0.4%) | 96.4% |
| **28** | Austria | 1 (0.2%) | 8 (0.6%) | 7 (0.4%) | 8 (0.4%) | 5 (0.2%) | 10 (0.4%) | 39 (0.4%) | 96.8% |
| **29** | Chile | 0 (0.0%) | 1 (0.1%) | 5 (0.3%) | 10 (0.5%) | 8 (0.4%) | 9 (0.4%) | 33 (0.3%) | 97.1% |
| **30** | Ireland | 1 (0.2%) | 3 (0.2%) | 4 (0.2%) | 4 (0.2%) | 7 (0.3%) | 8 (0.4%) | 27 (0.3%) | 97.3% |
| **31** | Qatar | 0 (0.0%) | 1 (0.1%) | 4 (0.2%) | 7 (0.3%) | 7 (0.3%) | 7 (0.3%) | 26 (0.3%) | 97.6% |
| **32** | Portugal | 0 (0.0%) | 1 (0.1%) | 4 (0.2%) | 5 (0.2%) | 8 (0.4%) | 7 (0.3%) | 25 (0.2%) | 97.8% |
| **33** | United Arab Emirates | 0 (0.0%) | 3 (0.2%) | 4 (0.2%) | 4 (0.2%) | 6 (0.3%) | 6 (0.3%) | 23 (0.2%) | 98.1% |
| **34** | Malaysia | 0 (0.0%) | 1 (0.1%) | 2 (0.1%) | 4 (0.2%) | 5 (0.2%) | 8 (0.4%) | 20 (0.2%) | 98.3% |
| **35** | Poland | 0 (0.0%) | 1 (0.1%) | 5 (0.3%) | 3 (0.1%) | 5 (0.2%) | 5 (0.2%) | 19 (0.2%) | 98.5% |
| **=35** | South Africa | 2 (0.4%) | 2 (0.1%) | 2 (0.1%) | 5 (0.2%) | 4 (0.2%) | 4 (0.2%) | 19 (0.2%) | 98.6% |
| **36** | Thailand | 1 (0.2%) | 1 (0.1%) | 3 (0.2%) | 5 (0.2%) | 3 (0.1%) | 2 (0.1%) | 15 (0.1%) | 98.8% |
| **37** | Russia | 0 (0.0%) | 1 (0.1%) | 3 (0.2%) | 1 (0.0%) | 5 (0.2%) | 3 (0.1%) | 13 (0.1%) | 98.9% |
| **38** | Jordan | 1 (0.2%) | 2 (0.1%) | 2 (0.1%) | 2 (0.1%) | 1 (0.0%) | 4 (0.2%) | 12 (0.1%) | 99.0% |
| **39** | Croatia | 0 (0.0%) | 0 (0.0%) | 1 (0.1%) | 1 (0.0%) | 4 (0.2%) | 2 (0.1%) | 8 (0.1%) | 99.1% |
| **=39** | Lithuania | 0 (0.0%) | 1 (0.1%) | 1 (0.1%) | 2 (0.1%) | 2 (0.1%) | 2 (0.1%) | 8 (0.1%) | 99.2% |
| **40** | Romania | 0 (0.0%) | 0 (0.0%) | 2 (0.1%) | 1 (0.0%) | 2 (0.1%) | 2 (0.1%) | 7 (0.1%) | 99.3% |
| **41** | Nigeria | 0 (0.0%) | 1 (0.1%) | 1 (0.1%) | 1 (0.0%) | 2 (0.1%) | 1 (0.0%) | 6 (0.1%) | 99.3% |
| **=41** | Yemen | 0 (0.0%) | 1 (0.1%) | 2 (0.1%) | 1 (0.0%) | 1 (0.0%) | 1 (0.0%) | 6 (0.1%) | 99.4% |
| **42** | Bulgaria | 0 (0.0%) | 1 (0.1%) | 1 (0.1%) | 1 (0.0%) | 1 (0.0%) | 1 (0.0%) | 5 (0.0%) | 99.4% |
| **=42** | Iceland | 1 (0.2%) | 1 (0.1%) | 1 (0.1%) | 0 (0.0%) | 1 (0.0%) | 1 (0.0%) | 5 (0.0%) | 99.5% |
| **=42** | Kuwait | 2 (0.4%) | 0 (0.0%) | 0 (0.0%) | 1 (0.0%) | 1 (0.0%) | 1 (0.0%) | 5 (0.0%) | 99.5% |
| **=42** | Liechtenstein | 0 (0.0%) | 1 (0.1%) | 1 (0.1%) | 1 (0.0%) | 1 (0.0%) | 1 (0.0%) | 5 (0.0%) | 99.6% |
| **=42** | Serbia | 0 (0.0%) | 1 (0.1%) | 0 (0.0%) | 1 (0.0%) | 1 (0.0%) | 2 (0.1%) | 5 (0.0%) | 99.6% |
| **43** | Bahrain | 0 (0.0%) | 0 (0.0%) | 1 (0.1%) | 1 (0.0%) | 1 (0.0%) | 1 (0.0%) | 4 (0.0%) | 99.7% |
| **=43** | Vietnam | 0 (0.0%) | 0 (0.0%) | 1 (0.1%) | 1 (0.0%) | 1 (0.0%) | 1 (0.0%) | 4 (0.0%) | 99.7% |
| **44** | Czech Republic | 0 (0.0%) | 0 (0.0%) | 0 (0.0%) | 0 (0.0%) | 1 (0.0%) | 2 (0.1%) | 3 (0.0%) | 99.7% |
| **=44** | Hungary | 0 (0.0%) | 0 (0.0%) | 1 (0.1%) | 1 (0.0%) | 0 (0.0%) | 1 (0.0%) | 3 (0.0%) | 99.8% |
| **=44** | Lebanon | 0 (0.0%) | 0 (0.0%) | 0 (0.0%) | 0 (0.0%) | 1 (0.0%) | 2 (0.1%) | 3 (0.0%) | 99.8% |
| **=44** | Pakistan | 0 (0.0%) | 0 (0.0%) | 1 (0.1%) | 1 (0.0%) | 0 (0.0%) | 1 (0.0%) | 3 (0.0%) | 99.8% |
| **45** | Georgia | 0 (0.0%) | 2 (0.1%) | 0 (0.0%) | 0 (0.0%) | 0 (0.0%) | 0 (0.0%) | 2 (0.0%) | 99.8% |
| **=45** | Indonesia | 0 (0.0%) | 1 (0.1%) | 1 (0.1%) | 0 (0.0%) | 0 (0.0%) | 0 (0.0%) | 2 (0.0%) | 99.9% |
| **=45** | Jamaica | 0 (0.0%) | 0 (0.0%) | 1 (0.1%) | 0 (0.0%) | 1 (0.0%) | 0 (0.0%) | 2 (0.0%) | 99.9% |
| **=45** | North Macedonia | 0 (0.0%) | 0 (0.0%) | 0 (0.0%) | 0 (0.0%) | 1 (0.0%) | 1 (0.0%) | 2 (0.0%) | 99.9% |
| **=45** | Slovenia | 0 (0.0%) | 1 (0.1%) | 0 (0.0%) | 1 (0.0%) | 0 (0.0%) | 0 (0.0%) | 2 (0.0%) | 99.9% |
| **46** | Armenia | 0 (0.0%) | 0 (0.0%) | 0 (0.0%) | 0 (0.0%) | 0 (0.0%) | 1 (0.0%) | 1 (0.0%) | 99.9% |
| **=46** | Bangladesh | 0 (0.0%) | 0 (0.0%) | 0 (0.0%) | 1 (0.0%) | 0 (0.0%) | 0 (0.0%) | 1 (0.0%) | 99.9% |
| **=46** | Cambodia | 0 (0.0%) | 0 (0.0%) | 0 (0.0%) | 0 (0.0%) | 0 (0.0%) | 1 (0.0%) | 1 (0.0%) | 100.0% |
| **=46** | Laos | 0 (0.0%) | 0 (0.0%) | 0 (0.0%) | 0 (0.0%) | 1 (0.0%) | 0 (0.0%) | 1 (0.0%) | 100.0% |
| **=46** | Mexico | 0 (0.0%) | 1 (0.1%) | 0 (0.0%) | 0 (0.0%) | 0 (0.0%) | 0 (0.0%) | 1 (0.0%) | 100.0% |
| **=46** | Malta | 1 (0.2%) | 0 (0.0%) | 0 (0.0%) | 0 (0.0%) | 0 (0.0%) | 0 (0.0%) | 1 (0.0%) | 100.0% |
| **=46** | Peru | 0 (0.0%) | 0 (0.0%) | 0 (0.0%) | 0 (0.0%) | 0 (0.0%) | 1 (0.0%) | 1 (0.0%) | 100.0% |
| **=46** | Puerto Rico | 1 (0.2%) | 0 (0.0%) | 0 (0.0%) | 0 (0.0%) | 0 (0.0%) | 0 (0.0%) | 1 (0.0%) | 100.0% |
| *NA* | *Missed* | 69 (11.0%) | 21 (1.5%) | 25 (1.4%) | 12 (0.6%) | 12 (0.6%) | 7 (0.3%) | 146 (1.4%) |  |
|  | **Total** | 628 | 1428 | 1846 | 2026 | 2173 | 2261 | 10326 |  |

**Table S3.** Scholar-level Analysis: Sex Stratification of Dental Scholars in the *Career-Long* Stanford-Elsevier Lists (SEL) of Top 2% Scientists Worldwide (2017–2023)

| **Rank** | **Country** | **SEL 2017** | **SEL 2018** | **SEL 2019** | **SEL 2020** | **SEL 2021** | **SEL 2022** | **SEL 2023** | **Total ▼** |
| --- | --- | --- | --- | --- | --- | --- | --- | --- | --- |
| **1** | USA | 32/272 (10.5%) | 40/298 (11.8%) | 76/458 (14.2%) | 92/564 (14.0%) | 105/621 (14.5%) | 106/654 (13.9%) | 126/681 (15.6%) | 577/3548 (14.0%) |
| **2** | GBR | 9/70 (11.4%) | 11/73 (13.1%) | 28/135 (17.2%) | 44/151 (22.6%) | 51/166 (23.5%) | 45/178 (20.2%) | 52/179 (22.5%) | 240/952 (20.1%) |
| **3** | SWE | 6/31 (16.2%) | 7/38 (15.6%) | 14/68 (17.1%) | 23/71 (24.5%) | 25/75 (25.0%) | 25/81 (23.6%) | 26/83 (23.9%) | 126/447 (22.0%) |
| **4** | DNK | 6/20 (23.1%) | 5/15 (25.0%) | 8/24 (25.0%) | 9/29 (23.7%) | 9/38 (19.1%) | 10/33 (23.3%) | 8/37 (17.8%) | 55/196 (21.9%) |
| **5** | CAN | 4/33 (10.8%) | 2/31 (6.1%) | 2/52 (3.7%) | 10/48 (17.2%) | 12/56 (17.6%) | 10/56 (15.2%) | 12/57 (17.4%) | 52/333 (13.5%) |
| **6** | FIN | 4/12 (25.0%) | 4/11 (26.7%) | 5/15 (25.0%) | 8/22 (26.7%) | 10/24 (29.4%) | 9/25 (26.5%) | 11/27 (28.9%) | 51/136 (27.3%) |
| **7** | JPN | 2/16 (11.1%) | 1/13 (7.1%) | 5/37 (11.9%) | 8/58 (12.1%) | 10/59 (14.5%) | 6/66 (8.3%) | 11/61 (15.3%) | 43/310 (12.2%) |
| **8** | DEU | 1/14 (6.7%) | 2/17 (10.5%) | 3/34 (8.1%) | 7/51 (12.1%) | 10/57 (14.9%) | 8/67 (10.7%) | 9/74 (10.8%) | 40/314 (11.3%) |
| **9** | ITA | 0/8 (0%) | 2/10 (16.7%) | 4/26 (13.3%) | 5/38 (11.6%) | 7/40 (14.9%) | 8/48 (14.3%) | 13/55 (19.1%) | 39/225 (14.8%) |
| **10** | CHE | 5/27 (15.6%) | 4/23 (14.8%) | 5/39 (11.4%) | 4/48 (7.7%) | 6/53 (10.2%) | 5/61 (7.6%) | 7/56 (11.1%) | 36/307 (10.5%) |
| **11** | NLD | 2/17 (10.5%) | 4/18 (18.2%) | 3/34 (8.1%) | 6/44 (12.0%) | 5/46 (9.8%) | 3/49 (5.8%) | 7/53 (11.7%) | 30/261 (10.3%) |
| **12** | NOR | 2/7 (22.2%) | 1/9 (10.0%) | 3/14 (17.6%) | 6/26 (18.8%) | 6/32 (15.8%) | 5/26 (16.1%) | 5/26 (16.1%) | 28/140 (16.7%) |
| **13** | BRA | 0/2 (0%) | 0/5 (0%) | 2/18 (10.0%) | 3/18 (14.3%) | 6/21 (22.2%) | 7/22 (24.1%) | 9/30 (23.1%) | 27/116 (18.9%) |
| **14** | AUS | 0/21 (0%) | 0/21 (0%) | 4/33 (10.8%) | 4/35 (10.3%) | 5/42 (10.6%) | 6/41 (12.8%) | 5/45 (10.0%) | 24/238 (9.2%) |
| **15** | BEL | 1/8 (11.1%) | 0/8 (0%) | 2/12 (14.3%) | 3/15 (16.7%) | 5/15 (25.0%) | 5/16 (23.8%) | 4/20 (16.7%) | 20/94 (17.5%) |
| **16** | ISR | 1/9 (10.0%) | 1/7 (12.5%) | 1/15 (6.3%) | 3/20 (13.0%) | 4/23 (14.8%) | 4/29 (12.1%) | 3/29 (9.4%) | 17/132 (11.4%) |
| **17** | FRA | 0/4 (0%) | 0/5 (0%) | 2/8 (20.0%) | 1/4 (20.0%) | 4/7 (36.4%) | 3/10 (23.1%) | 5/9 (35.7%) | 15/47 (24.2%) |
| **18** | ESP | 0/2 (0%) | 0/3 (0%) | 1/10 (9.1%) | 2/9 (18.2%) | 2/14 (12.5%) | 2/16 (11.1%) | 2/20 (9.1%) | 9/74 (10.8%) |
| **19** | HKG | 0/5 (0%) | 0/6 (0%) | 0/8 (0%) | 0/12 (0%) | 5/12 (29.4%) | 3/10 (23.1%) | 0/15 (0%) | 8/68 (10.5%) |
| **20** | IRL | 1/2 (33.3%) | 0/1 (0%) | 1/2 (33.3%) | 0/6 (0%) | 1/6 (14.3%) | 1/7 (12.5%) | 3/7 (30.0%) | 7/31 (18.4%) |
| **21** | TWN | 0/2 (0%) | 0/2 (0%) | 0/4 (0%) | 1/6 (14.3%) | 1/11 (8.3%) | 2/7 (22.2%) | 2/9 (18.2%) | 6/41 (12.8%) |
| **22** | EGY | *NA* | *NA* | 0/1 (0%) | 1/1 (50.0%) | 1/1 (50.0%) | 1/3 (25.0%) | 2/3 (40.0%) | 5/9 (35.7%) |
| **=22** | KOR | 0/1 (0%) | 0/2 (0%) | 0/6 (0%) | 1/5 (16.7%) | 1/9 (10.0%) | 0/11 (0%) | 3/8 (27.3%) | 5/42 (10.6%) |
| **23** | CHN | 0/1 (0%) | 0/1 (0%) | 0/4 (0%) | 0/6 (0%) | 0/10 (0%) | 1/8 (11.1%) | 3/10 (23.1%) | 4/40 (9.1%) |
| **24** | IND | 0/1 (0%) | *NA* | *NA* | 0/1 (0%) | 1/5 (16.7%) | 0/3 (0%) | 2/5 (28.6%) | 3/15 (16.7%) |
| **=24** | SGP | *NA* | 0/3 (0%) | 0/2 (0%) | 0/3 (0%) | 0/3 (0%) | 1/0 (100%) | 2/3 (40.0%) | 3/14 (17.6%) |
| **=24** | ZAF | 0/2 (0%) | 0/2 (0%) | 0/3 (0%) | 0/2 (0%) | 1/4 (20.0%) | 1/5 (16.7%) | 1/5 (16.7%) | 3/23 (11.5%) |
| **25** | ARE | *NA* | 0/3 (0%) | 2/5 (28.6%) | 0/6 (0%) | 0/6 (0%) | 0/5 (0%) | 0/7 (0%) | 2/32 (5.9%) |
| **=25** | AUT | 0/1 (0%) | 0/2 (0%) | 0/6 (0%) | 0/6 (0%) | 1/8 (11.1%) | 1/5 (16.7%) | 0/8 (0%) | 2/36 (5.3%) |
| **=25** | IRN | *NA* | *NA* | 0/2 (0%) | 0/3 (0%) | 0/2 (0%) | 0/6 (0%) | 2/6 (25.0%) | 2/19 (9.5%) |
| **=25** | SRB | *NA* | *NA* | *NA* | *NA* | 1/0 (100%) | 0/1 (0%) | 1/0 (100%) | 2/1 (66.7%) |
| **26** | BGR | *NA* | *NA* | *NA* | *NA* | *NA* | 1/0 (100%) | *NA* | 1/0 (100%) |
| **=26** | CHL | *NA* | *NA* | 0/1 (0%) | *NA* | 0/1 (0%) | *NA* | 1/0 (100%) | 1/2 (33.3%) |
| **=26** | GRC | 0/1 (0%) | *NA* | 0/4 (0%) | 0/7 (0%) | 0/7 (0%) | 0/8 (0%) | 1/8 (11.1%) | 1/35 (2.8%) |
| **=26** | MLT | 1/0 (100%) | *NA* | *NA* | *NA* | *NA* | *NA* | *NA* | 1/0 (100%) |
| **=26** | NGA | *NA* | *NA* | *NA* | 0/1 (0%) | 1/2 (33.3%) | 0/3 (0%) | 0/2 (0%) | 1/8 (11.1%) |
| **=26** | PRT | *NA* | *NA* | *NA* | 0/1 (0%) | 1/1 (50.0%) | 0/2 (0%) | 0/1 (0%) | 1/5 (16.7%) |
| **=26** | SVN | *NA* | *NA* | 0/1 (0%) | 1/0 (100%) | 0/1 (0%) | *NA* | *NA* | 1/2 (33.3%) |
| **27** | ARG | *NA* | *NA* | *NA* | *NA* | 0/1 (0%) | 0/1 (0%) | 0/1 (0%) | 0/3 (0%) |
| **=27** | CYP | *NA* | *NA* | *NA* | *NA* | 0/1 (0%) | 0/1 (0%) | 0/1 (0%) | 0/3 (0%) |
| **=27** | GEO | *NA* | *NA* | 0/1 (0%) | *NA* | *NA* | *NA* | *NA* | 0/1 (0%) |
| **=27** | HRV | *NA* | *NA* | *NA* | 0/1 (0%) | 0/1 (0%) | 0/1 (0%) | 0/1 (0%) | 0/4 (0%) |
| **=27** | HUN | *NA* | *NA* | *NA* | 0/1 (0%) | 0/1 (0%) | 0/3 (0%) | 0/3 (0%) | 0/8 (0%) |
| **=27** | IDN | *NA* | *NA* | 0/1 (0%) | *NA* | *NA* | *NA* | *NA* | 0/1 (0%) |
| **=27** | ISL | *NA* | *NA* | 0/1 (0%) | *NA* | *NA* | 0/1 (0%) | 0/2 (0%) | 0/4 (0%) |
| **=27** | JOR | *NA* | *NA* | 0/1 (0%) | 0/1 (0%) | 0/1 (0%) | 0/1 (0%) | 0/2 (0%) | 0/6 (0%) |
| **=27** | KWT | 0/3 (0%) | 0/2 (0%) | *NA* | 0/1 (0%) | *NA* | *NA* | *NA* | 0/6 (0%) |
| **=27** | LAO | *NA* | *NA* | *NA* | *NA* | *NA* | 0/1 (0%) | *NA* | 0/1 (0%) |
| **=27** | LBN | *NA* | *NA* | *NA* | 0/1 (0%) | 0/1 (0%) | 0/1 (0%) | 0/1 (0%) | 0/4 (0%) |
| **=27** | LIE | *NA* | 0/1 (0%) | *NA* | 0/1 (0%) | *NA* | 0/1 (0%) | 0/1 (0%) | 0/4 (0%) |
| **=27** | MEX | *NA* | *NA* | 0/2 (0%) | 0/2 (0%) | 0/1 (0%) | 0/2 (0%) | 0/1 (0%) | 0/8 (0%) |
| **=27** | MYS | *NA* | *NA* | *NA* | *NA* | 0/1 (0%) | 0/2 (0%) | 0/3 (0%) | 0/6 (0%) |
| **=27** | NZL | 0/3 (0%) | 0/2 (0%) | 0/5 (0%) | 0/8 (0%) | 0/9 (0%) | 0/8 (0%) | 0/9 (0%) | 0/44 (0%) |
| **=27** | OMN | *NA* | *NA* | *NA* | 0/1 (0%) | 0/1 (0%) | *NA* | *NA* | 0/2 (0%) |
| **=27** | PAK | *NA* | *NA* | *NA* | *NA* | 0/1 (0%) | 0/1 (0%) | 0/1 (0%) | 0/3 (0%) |
| **=27** | POL | *NA* | *NA* | *NA* | 0/1 (0%) | 0/1 (0%) | 0/1 (0%) | *NA* | 0/3 (0%) |
| **=27** | PRI | 0/1 (0%) | *NA* | *NA* | *NA* | *NA* | *NA* | *NA* | 0/1 (0%) |
| **=27** | QAT | *NA* | *NA* | *NA* | 0/3 (0%) | 0/2 (0%) | 0/2 (0%) | 0/2 (0%) | 0/9 (0%) |
| **=27** | ROU | *NA* | *NA* | 0/1 (0%) | 0/1 (0%) | *NA* | *NA* | *NA* | 0/2 (0%) |
| **=27** | RUS | *NA* | *NA* | *NA* | *NA* | *NA* | 0/3 (0%) | 0/1 (0%) | 0/4 (0%) |
| **=27** | SAU | *NA* | *NA* | 0/1 (0%) | 0/2 (0%) | 0/1 (0%) | 0/3 (0%) | 0/4 (0%) | 0/11 (0%) |
| **=27** | THA | *NA* | 0/1 (0%) | 0/1 (0%) | 0/1 (0%) | 0/1 (0%) | 0/1 (0%) | 0/1 (0%) | 0/6 (0%) |
| **=27** | TUR | 0/1 (0%) | 0/1 (0%) | 0/1 (0%) | 0/5 (0%) | 0/6 (0%) | 0/5 (0%) | 0/6 (0%) | 0/25 (0%) |
| **=27** | YEM | *NA* | *NA* | *NA* | *NA* | *NA* | 0/1 (0%) | 0/1 (0%) | 0/2 (0%) |
| **=27** | ZWE | *NA* | *NA* | *NA* | 0/1 (0%) | *NA* | 0/1 (0%) | 0/1 (0%) | 0/3 (0%) |
| *NA* | *Missed* | 6/82 (6.8%) | 0/2 (0%) | 0/8 (0%) | 3/20 (13.0%) | 1/14 (6.7%) | 1/16 (5.9%) | 0/12 (0%) | 11/154 (6.7%) |
|  | **Total** | 83/679  (10.9%) | 84/636  (11.7%) | 171/1104 (13.4%) | 245/1368  (15.2%) | 298/1522  (16.4%) | 280/1619  (14.7%) | 338/1693  (16.6%) | 1499/8621  (14.8%) |

Female / Male (Female/Both Sexes*100)

**Table S4.** Scholar-level Analysis: Sex Stratification of Dental Scholars in the *Single-Year* Stanford-Elsevier Lists (SEL) of Top 2% Scientists Worldwide (2017–2023)

| **Rank** | **Country** | **SEL 2017** | **SEL 2019** | **SEL 2020** | **SEL 2021** | **SEL 2022** | **SEL 2023** | **Total ▼** |
| --- | --- | --- | --- | --- | --- | --- | --- | --- |
| **1** | USA | 28/185 (13.1%) | 71/388 (15.5%) | 93/473 (16.4%) | 101/490 (17.1%) | 108/489 (18.1%) | 115/522 (18.1%) | 516/2547 (16.8%) |
| **2** | GBR | 11/59 (15.7%) | 32/101 (24.1%) | 41/125 (24.7%) | 37/136 (21.4%) | 36/154 (18.9%) | 37/143 (20.6%) | 194/718 (21.3%) |
| **3** | ITA | 1/19 (5.0%) | 20/70 (22.2%) | 26/85 (23.4%) | 25/114 (18.0%) | 38/112 (25.3%) | 47/112 (29.6%) | 157/512 (23.5%) |
| **4** | SWE | 5/18 (21.7%) | 12/47 (20.3%) | 15/52 (22.4%) | 12/48 (20.0%) | 14/41 (25.5%) | 17/39 (30.4%) | 75/245 (23.4%) |
| **5** | DEU | 5/18 (21.7%) | 11/57 (16.2%) | 11/79 (12.2%) | 10/91 (9.9%) | 18/96 (15.8%) | 16/97 (14.2%) | 71/438 (13.9%) |
| **6** | BRA | 1/10 (9.1%) | 8/39 (17.0%) | 11/56 (16.4%) | 18/70 (20.5%) | 17/69 (19.8%) | 13/73 (15.1%) | 68/317 (17.7%) |
| **7** | CHN | 1/3 (25.0%) | 4/14 (22.2%) | 9/23 (28.1%) | 9/32 (22.0%) | 17/46 (27.0%) | 23/60 (27.7%) | 63/178 (26.1%) |
| **8** | CHE | 8/27 (22.9%) | 10/46 (17.9%) | 11/55 (16.7%) | 9/58 (13.4%) | 9/63 (12.5%) | 11/61 (15.3%) | 58/310 (15.8%) |
| **=8** | DNK | 5/10 (33.3%) | 8/20 (28.6%) | 11/26 (29.7%) | 14/24 (36.8%) | 8/25 (24.2%) | 12/21 (36.4%) | 58/126 (31.5%) |
| **9** | JPN | 2/8 (20.0%) | 6/35 (14.6%) | 9/45 (16.7%) | 10/62 (13.9%) | 9/63 (12.5%) | 14/62 (18.4%) | 50/275 (15.4%) |
| **10** | NLD | 2/13 (13.3%) | 7/35 (16.7%) | 4/45 (8.2%) | 7/45 (13.5%) | 7/49 (12.5%) | 13/48 (21.3%) | 40/235 (14.5%) |
| **11** | AUS | 2/15 (11.8%) | 3/33 (8.3%) | 4/45 (8.2%) | 9/48 (15.8%) | 8/49 (14.0%) | 13/45 (22.4%) | 39/235 (14.2%) |
| **12** | CAN | 2/23 (8.0%) | 4/40 (9.1%) | 7/44 (13.7%) | 8/46 (14.8%) | 4/51 (7.3%) | 10/53 (15.9%) | 35/257 (12.0%) |
| **13** | BEL | 2/10 (16.7%) | 6/12 (33.3%) | 8/13 (38.1%) | 4/22 (15.4%) | 5/21 (19.2%) | 7/20 (25.9%) | 32/98 (24.6%) |
| **14** | FIN | 3/5 (37.5%) | 3/9 (25.0%) | 5/10 (33.3%) | 7/15 (31.8%) | 6/13 (31.6%) | 7/13 (35.0%) | 31/65 (32.3%) |
| **15** | ESP | 0/2 (0.0%) | 3/13 (18.8%) | 6/22 (21.4%) | 5/33 (13.2%) | 4/31 (11.4%) | 7/34 (17.1%) | 25/135 (15.6%) |
| **16** | KOR | 2/2 (50.0%) | 3/12 (20.0%) | 2/19 (9.5%) | 3/23 (11.5%) | 7/29 (19.4%) | 7/32 (17.9%) | 24/117 (17.0%) |
| **17** | IND | *NA* | 2/3 (40.0%) | 3/11 (21.4%) | 5/18 (21.7%) | 3/19 (13.6%) | 9/18 (33.3%) | 22/69 (24.2%) |
| **18** | ISR | 1/5 (16.7%) | 2/8 (20.0%) | 3/17 (15.0%) | 3/17 (15.0%) | 4/20 (16.7%) | 6/16 (27.3%) | 19/83 (18.6%) |
| **19** | NOR | 1/3 (25.0%) | 4/7 (36.4%) | 3/14 (17.6%) | 2/18 (10.0%) | 3/15 (16.7%) | 3/11 (21.4%) | 16/68 (19.0%) |
| **20** | GRC | 0/1 (0.0%) | 0/2 (0.0%) | 4/6 (40.0%) | 2/10 (16.7%) | 4/8 (33.3%) | 4/8 (33.3%) | 14/35 (28.6%) |
| **21** | EGY | 0/2 (0.0%) | 1/2 (33.3%) | 2/5 (28.6%) | 2/4 (33.3%) | 3/6 (33.3%) | 3/8 (27.3%) | 11/27 (28.9%) |
| **=21** | SAU | *NA* | 0/7 (0.0%) | 2/11 (15.4%) | 2/15 (11.8%) | 2/23 (8.0%) | 5/22 (18.5%) | 11/78 (12.4%) |
| **22** | FRA | 0/2 (0.0%) | 0/6 (0.0%) | 1/7 (12.5%) | 3/12 (20.0%) | 3/13 (18.8%) | 3/15 (16.7%) | 10/55 (15.4%) |
| **=22** | IRN | 0/2 (0.0%) | 0/7 (0.0%) | 0/13 (0.0%) | 2/11 (15.4%) | 3/14 (17.6%) | 5/17 (22.7%) | 10/64 (13.5%) |
| **23** | PRT | *NA* | 0/1 (0.0%) | 0/4 (0.0%) | 2/2 (50.0%) | 3/3 (50.0%) | 3/4 (42.9%) | 8/14 (36.4%) |
| **24** | IRL | 0/1 (0.0%) | 2/1 (66.7%) | 1/3 (25.0%) | 1/3 (25.0%) | 2/5 (28.6%) | 1/7 (12.5%) | 7/20 (25.9%) |
| **=24** | POL | *NA* | 0/1 (0.0%) | 3/2 (60.0%) | 1/2 (33.3%) | 1/4 (20.0%) | 2/3 (40.0%) | 7/12 (36.8%) |
| **=24** | TWN | 1/1 (50.0%) | 0/9 (0.0%) | 0/12 (0.0%) | 2/12 (14.3%) | 1/13 (7.1%) | 3/11 (21.4%) | 7/58 (10.8%) |
| **25** | AUT | 0/1 (0.0%) | 1/6 (14.3%) | 1/6 (14.3%) | 1/6 (14.3%) | 1/3 (25.0%) | 2/8 (20.0%) | 6/30 (16.7%) |
| **=25** | HKG | 0/6 (0.0%) | 0/19 (0.0%) | 1/16 (5.9%) | 3/16 (15.8%) | 1/15 (6.3%) | 1/15 (6.3%) | 6/87 (6.5%) |
| **=25** | TUR | *NA* | 1/3 (25.0%) | 0/11 (0.0%) | 2/10 (16.7%) | 1/12 (7.7%) | 2/10 (16.7%) | 6/46 (11.5%) |
| **26** | NZL | 0/2 (0.0%) | 0/7 (0.0%) | 0/8 (0.0%) | 1/9 (10.0%) | 2/8 (20.0%) | 1/9 (10.0%) | 4/43 (8.5%) |
| **=26** | QAT | *NA* | 0/1 (0.0%) | 0/3 (0.0%) | 0/5 (0.0%) | 1/4 (20.0%) | 3/3 (50.0%) | 4/16 (20.0%) |
| **27** | ARE | *NA* | 0/3 (0.0%) | 0/4 (0.0%) | 0/4 (0.0%) | 2/4 (33.3%) | 1/5 (16.7%) | 3/20 (13.0%) |
| **=27** | LTU | *NA* | 0/1 (0.0%) | 0/1 (0.0%) | 1/1 (50.0%) | 1/1 (50.0%) | 1/1 (50.0%) | 3/5 (37.5%) |
| **=27** | SGP | *NA* | 0/5 (0.0%) | 0/7 (0.0%) | 0/6 (0.0%) | 1/5 (16.7%) | 2/11 (15.4%) | 3/34 (8.1%) |
| **=27** | SRB | *NA* | 1/0 (100.0%) | *NA* | 1/0 (100.0%) | 0/1 (0.0%) | 1/1 (50.0%) | 3/2 (60.0%) |
| **28** | BGR | *NA* | 0/1 (0.0%) | 1/0 (100.0%) | *NA* | 1/0 (100.0%) | *NA* | 2/1 (66.7%) |
| **=28** | HRV | *NA* | *NA* | 0/1 (0.0%) | 0/1 (0.0%) | 1/3 (25.0%) | 1/1 (50.0%) | 2/6 (25.0%) |
| **=28** | NGA | *NA* | 0/1 (0.0%) | 1/0 (100.0%) | *NA* | 1/1 (50.0%) | 0/1 (0.0%) | 2/3 (40.0%) |
| **=28** | ROU | *NA* | *NA* | 0/2 (0.0%) | 0/1 (0.0%) | 0/2 (0.0%) | 2/0 (100.0%) | 2/5 (28.6%) |
| **=28** | ZAF | 0/2 (0.0%) | *NA* | 1/1 (50.0%) | 0/5 (0.0%) | 1/3 (25.0%) | 0/4 (0.0%) | 2/15 (11.8%) |
| **29** | CHL | *NA* | 0/1 (0.0%) | 0/4 (0.0%) | 0/9 (0.0%) | 1/5 (16.7%) | 0/8 (0.0%) | 1/27 (3.6%) |
| **=29** | JOR | 0/1 (0.0%) | 0/2 (0.0%) | 0/2 (0.0%) | 0/2 (0.0%) | 1/0 (100.0%) | 0/4 (0.0%) | 1/11 (8.3%) |
| **=29** | LIE | *NA* | 0/1 (0.0%) | *NA* | 1/0 (100.0%) | *NA* | 0/1 (0.0%) | 1/2 (33.3%) |
| **=29** | MLT | 1/0 (100.0%) | *NA* | *NA* | *NA* | *NA* | *NA* | 1/0 (100.0%) |
| **=29** | MYS | *NA* | 0/1 (0.0%) | 0/2 (0.0%) | 0/4 (0.0%) | 1/3 (25.0%) | 0/8 (0.0%) | 1/18 (5.3%) |
| **=29** | PER | *NA* | *NA* | *NA* | *NA* | *NA* | 1/0 (100.0%) | 1/0 (100.0%) |
| **=29** | PRI | 1/0 (100.0%) | *NA* | *NA* | *NA* | *NA* | *NA* | 1/0 (100.0%) |
| **=29** | THA | 0/1 (0.0%) | 0/1 (0.0%) | 0/3 (0.0%) | 0/5 (0.0%) | 0/2 (0.0%) | 1/0 (100.0%) | 1/12 (7.7%) |
| **30** | ARM | *NA* | *NA* | *NA* | *NA* | *NA* | 0/1 (0.0%) | 0/1 (0.0%) |
| **=30** | BGD | *NA* | *NA* | *NA* | 0/1 (0.0%) | *NA* | *NA* | 0/1 (0.0%) |
| **=30** | BHR | *NA* | *NA* | 0/1 (0.0%) | 0/1 (0.0%) | 0/1 (0.0%) | 0/1 (0.0%) | 0/4 (0.0%) |
| **=30** | CZE | *NA* | *NA* | *NA* | *NA* | 0/1 (0.0%) | 0/2 (0.0%) | 0/3 (0.0%) |
| **=30** | GEO | *NA* | 0/2 (0.0%) | *NA* | *NA* | *NA* | *NA* | 0/2 (0.0%) |
| **=30** | HUN | *NA* | *NA* | 0/1 (0.0%) | 0/1 (0.0%) | *NA* | 0/1 (0.0%) | 0/3 (0.0%) |
| **=30** | IDN | *NA* | 0/1 (0.0%) | *NA* | *NA* | *NA* | *NA* | 0/1 (0.0%) |
| **=30** | ISL | 0/1 (0.0%) | 0/1 (0.0%) | 0/1 (0.0%) | *NA* | 0/1 (0.0%) | 0/1 (0.0%) | 0/5 (0.0%) |
| **=30** | JAM | *NA* | *NA* | *NA* | *NA* | 0/1 (0.0%) | *NA* | 0/1 (0.0%) |
| **=30** | KHM | *NA* | *NA* | *NA* | *NA* | *NA* | 0/1 (0.0%) | 0/1 (0.0%) |
| **=30** | KWT | 0/2 (0.0%) | *NA* | *NA* | 0/1 (0.0%) | 0/1 (0.0%) | 0/1 (0.0%) | 0/5 (0.0%) |
| **=30** | LAO | *NA* | *NA* | *NA* | *NA* | 0/1 (0.0%) | *NA* | 0/1 (0.0%) |
| **=30** | LBN | *NA* | *NA* | *NA* | *NA* | 0/1 (0.0%) | 0/2 (0.0%) | 0/3 (0.0%) |
| **=30** | MEX | *NA* | 0/1 (0.0%) | *NA* | *NA* | *NA* | *NA* | 0/1 (0.0%) |
| **=30** | MKD | *NA* | *NA* | *NA* | *NA* | *NA* | 0/1 (0.0%) | 0/1 (0.0%) |
| **=30** | PAK | *NA* | *NA* | 0/1 (0.0%) | 0/1 (0.0%) | *NA* | 0/1 (0.0%) | 0/3 (0.0%) |
| **=30** | RUS | *NA* | 0/1 (0.0%) | 0/3 (0.0%) | 0/1 (0.0%) | 0/4 (0.0%) | 0/3 (0.0%) | 0/12 (0.0%) |
| **=30** | SVN | *NA* | *NA* | *NA* | 0/1 (0.0%) | *NA* | *NA* | 0/1 (0.0%) |
| **=30** | VNM | *NA* | *NA* | 0/1 (0.0%) | 0/1 (0.0%) | 0/1 (0.0%) | 0/1 (0.0%) | 0/4 (0.0%) |
| **=30** | YEM | *NA* | *NA* | 0/2 (0.0%) | 0/1 (0.0%) | 0/1 (0.0%) | 0/1 (0.0%) | 0/5 (0.0%) |
| *NA* | *Missed* | 11/54 (16.9%) | 2/13 (13.3%) | 4/18 (18.2%) | 2/10 (16.7%) | 0/11 (0.0%) | 2/5 (28.6%) | 21/111 (15.9%) |
|  | **Total** | 96/514 (15.7%) | 227/1097 (17.1%) | 304/1421 (17.6%) | 327/1584 (17.1%) | 364/1640 (18.2%) | 437/1687 (20.6%) | 1755/7943 (18.1%) |

Female / Male (Female/Both Sexes*100)

**Table S5.** Scholar-Level Analysis: Sex Stratification of Scholarly Output Metrics of Dental Scholars in the Stanford-Elsevier Lists (SEL) of Top Scientists Worldwide (2017–2023)

| ***Career-Long*** | | | | | | | | | | | | | |
| --- | --- | --- | --- | --- | --- | --- | --- | --- | --- | --- | --- | --- | --- |
| **Variable** | **Outcome** | **Female** | | | **Male** | | | ***p.*** | | | | |  |
|  |  | **C-Score:**  **Median (IQR)** | **Modified H:**  **Median (IQR)** | **Self-citations:**  **Median (IQR)** | **C-Score:**  **Median (IQR)** | **Modified H:**  **Median (IQR)** | **Self-citations:**  **Median (IQR)** | **C-Score** | | **Modified H** | | **Self-citations** | |
| **Year** | SEL 2017 | 3.70 (3.51–3.82) | 17.53 (14.83–21.96) | 0.11 (0.07–0.14) | 3.60 (3.48–3.80) | 17.73 (14.98–21.14) | 0.10 (0.06–0.14) | 0.144 | | 0.992 | | 0.224 | |
|  | SEL 2018 | 3.57 (3.36–3.68) | 18.38 (15.26–22.35) | 0.11 (0.07–0.14) | 3.48 (3.36–3.69) | 18.80 (16.12–22.73) | 0.10 (0.06–0.13) | 0.423 | | 0.225 | | 0.050 | |
|  | SEL 2019 | 3.44 (3.29–3.61) | 15.77 (12.93–19.65) | 0.10 (0.07–0.13) | 3.43 (3.27–3.65) | 16.20 (13.64–19.99) | 0.10 (0.06–0.14) | 0.939 | | 0.079 | | 0.448 | |
|  | SEL 2020 | 3.36 (3.21–3.55) | 15.73 (12.83–18.71) | 0.10 (0.07–0.13) | 3.38 (3.20–3.60) | 15.92 (13.17–19.99) | 0.09 (0.06–0.14) | 0.582 | | **0.040** | | 0.830 | |
|  | SEL 2021 | 3.29 (3.15–3.50) | 14.82 (11.80–17.88) | 0.10 (0.07–0.13) | 3.35 (3.17–3.59) | 15.82 (12.95–19.95) | 0.09 (0.06–0.13) | **0.007** | | **<0.001** | | 0.222 | |
|  | SEL 2022 | 3.29 (3.16–3.50) | 14.91 (11.94–18.86) | 0.10 (0.07–0.13) | 3.34 (3.16–3.58) | 15.96 (13.18–20.44) | 0.09 (0.06–0.13) | **0.037** | | **<0.001** | | 0.099 | |
|  | SEL 2023 | 3.31 (3.14–3.47) | 15.34 (12.31–19.01) | 0.09 (0.07–0.13) | 3.32 (3.15–3.57) | 15.96 (13.24–20.53) | 0.09 (0.06–0.13) | **0.035** | | **0.001** | | **0.042** | |
| **World Bank** | High | 3.36 (3.19–3.56) | 15.70 (12.47–19.00) | 0.10 (0.07–0.13) | 3.41 (3.22–3.63) | 16.54 (13.66–20.69) | 0.09 (0.06–0.13) | **<0.001** | | **<0.001** | | **0.008** | |
|  | Upper-middle | 3.26 (3.08–3.32) | 14.92 (13.20–19.76) | 0.16 (0.11–0.22) | 3.25 (3.13–3.45) | 15.60 (12.73–18.97) | 0.14 (0.08–0.18) | 0.192 | | 0.972 | | 0.075 | |
|  | Lower-middle | 3.24 (3.15–3.36) | 15.53 (12.83–17.87) | 0.05 (0.04–0.09) | 3.13 (3.10–3.17) | 12.50 (10.90–13.84) | 0.05 (0.03–0.09) | 0.065 | | 0.101 | | 0.887 | |
|  | Low | *NA* | *NA* | *NA* | 3.14 (3.12–3.17) | 13.12 (12.86–13.38) | 0.05 (0.04–0.05) | *NA* | | *NA* | | *NA* | |
| **Official Language** | English | 3.34 (3.19–3.54) | 14.94 (12.00–18.01) | 0.10 (0.06–0.12) | 3.44 (3.24–3.65) | 16.69 (13.70–20.84) | 0.09 (0.05–0.12) | 0.298 | | 0.170 | | 0.771 | |
|  | German | 3.49 (3.21–3.74) | 18.27 (14.77–21.26) | 0.10 (0.09–0.16) | 3.38 (3.18–3.67) | 17.77 (13.92–21.59) | 0.11 (0.08–0.15) | 0.100 | | 0.402 | | 0.924 | |
|  | Swedish | 3.45 (3.18–3.83) | 17.43 (13.19–24.56) | 0.08 (0.05–0.11) | 3.40 (3.25–3.66) | 16.70 (13.46–20.03) | 0.07 (0.05–0.10) | 0.951 | | 0.952 | | 0.336 | |
|  | Dutch | 3.41 (3.26–3.51) | 17.42 (14.10–20.15) | 0.11 (0.09–0.16) | 3.40 (3.24–3.64) | 18.93 (15.26–24.15) | 0.10 (0.08–0.13) | 0.490 | | 0.930 | | 0.706 | |
|  | Japanese | 3.44 (3.27–3.71) | 16.50 (12.96–18.68) | 0.11 (0.09–0.14) | 3.25 (3.12–3.41) | 14.92 (12.96–16.95) | 0.13 (0.11–0.17) | 0.186 | | 0.668 | | 0.614 | |
|  | Danish | 3.49 (3.36–3.62) | 15.77 (13.95–20.45) | 0.09 (0.08–0.14) | 3.43 (3.21–3.65) | 15.91 (13.90–23.23) | 0.08 (0.05–0.12) | 0.196 | | 0.050 | | 0.333 | |
|  | Italian | 3.27 (3.12–3.36) | 14.98 (13.04–16.96) | 0.17 (0.14–0.24) | 3.30 (3.15–3.55) | 15.15 (12.58–19.85) | 0.15 (0.11–0.19) | 0.055 | | 0.503 | | 0.481 | |
|  | Finnish | 3.37 (3.14–3.77) | 16.77 (12.14–27.56) | 0.11 (0.07–0.14) | 3.37 (3.17–3.54) | 16.84 (14.19–19.78) | 0.12 (0.09–0.18) | 0.444 | | **0.031** | | 0.676 | |
|  | Norwegian | 3.37 (3.17–3.71) | 13.86 (10.99–21.31) | 0.09 (0.05–0.12) | 3.35 (3.20–3.52) | 14.99 (13.23–18.97) | 0.06 (0.04–0.10) | 0.541 | | 0.591 | | 0.864 | |
|  | Portuguese | 3.28 (3.22–3.33) | 16.02 (13.98–20.99) | 0.16 (0.10–0.20) | 3.26 (3.12–3.44) | 16.29 (13.98–18.94) | 0.17 (0.14–0.19) | 0.861 | | **0.025** | | 0.062 | |
|  | Hebrew | 3.20 (3.13–3.48) | 15.58 (12.10–16.50) | 0.08 (0.05–0.09) | 3.31 (3.15–3.50) | 15.00 (12.78–17.33) | 0.09 (0.06–0.11) | **0.003** | | **<0.001** | | 0.054 | |
|  | Mandarin Chinese | 3.08 (2.69–3.18) | 16.55 (9.90–20.99) | 0.19 (0.12–0.44) | 3.31 (3.11–3.46) | 17.24 (12.99–20.62) | 0.14 (0.10–0.22) | 0.760 | | 0.688 | | 0.850 | |
|  | Spanish | 3.18 (3.13–3.31) | 18.11 (16.74–18.90) | 0.08 (0.07–0.19) | 3.31 (3.15–3.54) | 15.26 (13.55–19.87) | 0.11 (0.07–0.17) | 0.995 | | 0.499 | | 0.905 | |
|  | Arabic | 3.36 (3.28–3.60) | 17.87 (16.03–21.87) | 0.06 (0.05–0.12) | 3.33 (3.16–3.39) | 13.99 (12.66–15.77) | 0.06 (0.05–0.09) | **0.025** | | 0.415 | | 0.594 | |
|  | Chinese | 3.23 (3.17–3.30) | 16.77 (15.97–18.12) | 0.14 (0.08–0.21) | 3.39 (3.24–3.53) | 20.20 (16.76–23.24) | 0.11 (0.09–0.15) | 0.373 | | 0.558 | | 0.644 | |
|  | *Other* | 3.31 (3.21–3.43) | 14.05 (12.91–16.06) | 0.09 (0.08–0.14) | 3.32 (3.15–3.52) | 14.69 (11.99–17.88) | 0.10 (0.05–0.14) | 0.096 | | 0.153 | | 0.562 | |
| **Total** | | 3.35 (3.19–3.56) | 15.69 (12.49–19.00) | 0.10 (0.07–0.13) | 3.40 (3.21–3.62) | 16.47 (13.60–20.61) | 0.09 (0.06–0.13) | **<0.001** | | **<0.001** | | **0.002** | |
| ***Single-Year*** | | | | | | | | | | | | | |
| **Variable** | **Outcome** | **Female** | | | **Male** | | | | ***p.*** | | | | |
|  |  | **C-Score:**  **Median (IQR)** | **Modified H:**  **Median (IQR)** | **Self-citations:**  **Median (IQR)** | **C-Score:**  **Median (IQR)** | **Modified H:**  **Median (IQR)** | **Self-citations:**  **Median (IQR)** | **C-Score** | | **Modified H** | | **Self-citations** | |
| **Year** | SEL 2017 | 2.87 (2.74–3.13) | 5.01 (4.63–5.94) | 0.08 (0.04–0.11) | 2.89 (2.72–3.09) | 5.04 (4.50–6.07) | 0.08 (0.05–0.14) | 0.649 | | 0.752 | | 0.139 | |
|  | SEL 2019 | 2.66 (2.55–2.85) | 5.02 (4.54–6.11) | 0.09 (0.05–0.15) | 2.65 (2.50–2.89) | 5.06 (4.53–6.41) | 0.08 (0.04–0.14) | 0.457 | | 0.672 | | 0.331 | |
|  | SEL 2020 | 2.69 (2.54–2.90) | 5.87 (4.83–6.92) | 0.09 (0.05–0.15) | 2.67 (2.51–2.92) | 5.82 (4.87–6.96) | 0.09 (0.04–0.14) | 0.628 | | 0.950 | | 0.129 | |
|  | SEL 2021 | 2.53 (2.40–2.74) | 4.81 (3.86–5.71) | 0.09 (0.04–0.15) | 2.52 (2.37–2.78) | 4.75 (3.90–5.79) | 0.08 (0.04–0.13) | 0.634 | | 0.886 | | 0.059 | |
|  | SEL 2022 | 2.46 (2.33–2.71) | 4.65 (3.91–5.55) | 0.09 (0.05–0.14) | 2.51 (2.37–2.76) | 4.82 (3.92–5.84) | 0.08 (0.04–0.13) | **0.011** | | 0.102 | | **0.016** | |
|  | SEL 2023 | 2.44 (2.30–2.66) | 4.48 (3.77–5.40) | 0.08 (0.05–0.13) | 2.48 (2.34–2.72) | 4.78 (3.90–5.78) | 0.08 (0.04–0.12) | **<0.001** | | **0.002** | | 0.134 | |
| **World Bank** | High | 2.59 (2.41–2.82) | 4.88 (3.97–5.93) | 0.08 (0.05–0.14) | 2.60 (2.43–2.86) | 4.93 (4.01–5.99) | 0.08 (0.04–0.13) | **0.001** | | **<0.001** | | **0.001** | |
|  | Upper-middle | 2.46 (2.34–2.70) | 4.96 (3.93–6.00) | 0.11 (0.07–0.17) | 2.46 (2.35–2.64) | 4.88 (3.97–5.84) | 0.10 (0.06–0.16) | 0.547 | | 0.142 | | 0.269 | |
|  | Lower-middle | 2.47 (2.32–2.68) | 3.83 (3.13–5.96) | 0.04 (0.02–0.10) | 2.46 (2.33–2.70) | 3.92 (3.56–4.67) | 0.09 (0.03–0.14) | 0.844 | | 0.954 | | **0.040** | |
|  | Low | *NA* | *NA* | *NA* | 2.86 (2.79–2.86) | 5.03 (4.99–5.57) | 0.05 (0.04–0.09) | *NA* | | *NA* | | *NA* | |
| **Official Language** | English | 2.62 (2.44–2.83) | 4.89 (3.97–5.88) | 0.07 (0.04–0.11) | 2.65 (2.46–2.91) | 4.94 (4.06–6.00) | 0.07 (0.03–0.11) | 0.960 | | 0.855 | | 0.530 | |
|  | German | 2.69 (2.44–2.94) | 5.30 (4.37–6.67) | 0.10 (0.06–0.13) | 2.61 (2.43–2.90) | 5.37 (4.38–6.77) | 0.11 (0.07–0.15) | 0.654 | | 0.701 | | 0.169 | |
|  | Italian | 2.43 (2.29–2.61) | 4.45 (3.84–5.00) | 0.18 (0.12–0.25) | 2.54 (2.37–2.78) | 4.91 (3.95–5.91) | 0.14 (0.09–0.20) | 0.424 | | 0.587 | | 0.963 | |
|  | Portuguese | 2.50 (2.38–2.74) | 5.16 (4.05–6.70) | 0.15 (0.09–0.20) | 2.46 (2.35–2.63) | 4.92 (4.18–5.91) | 0.14 (0.09–0.18) | 0.252 | | 0.949 | | 0.771 | |
|  | Dutch | 2.70 (2.45–2.84) | 5.81 (4.60–6.70) | 0.09 (0.06–0.13) | 2.66 (2.46–2.90) | 5.89 (4.88–7.43) | 0.08 (0.05–0.12) | 0.152 | | 0.569 | | 0.051 | |
|  | Mandarin Chinese | 2.50 (2.31–2.69) | 5.00 (4.05–5.93) | 0.10 (0.06–0.14) | 2.46 (2.34–2.68) | 4.85 (3.92–5.90) | 0.09 (0.05–0.13) | 0.578 | | 0.093 | | 0.699 | |
|  | Swedish | 2.71 (2.52–3.11) | 4.92 (4.33–7.27) | 0.05 (0.02–0.11) | 2.68 (2.51–2.93) | 5.33 (4.63–6.81) | 0.05 (0.02–0.10) | 0.361 | | 0.851 | | 0.657 | |
|  | Japanese | 2.51 (2.40–2.67) | 4.54 (3.73–5.25) | 0.10 (0.06–0.13) | 2.46 (2.34–2.59) | 4.26 (3.84–4.97) | 0.10 (0.06–0.15) | 0.703 | | 0.730 | | 0.357 | |
|  | Arabic | 2.46 (2.32–2.77) | 4.11 (3.81–5.80) | 0.04 (0.02–0.11) | 2.48 (2.36–2.71) | 4.57 (3.72–5.37) | 0.09 (0.04–0.16) | 0.525 | | 0.905 | | 0.881 | |
|  | Spanish | 2.62 (2.38–2.69) | 4.97 (4.51–5.93) | 0.15 (0.08–0.18) | 2.48 (2.37–2.64) | 4.80 (3.93–5.84) | 0.12 (0.08–0.16) | 0.688 | | 0.337 | | 0.183 | |
|  | Danish | 2.75 (2.47–2.90) | 5.41 (4.71–6.64) | 0.05 (0.03–0.10) | 2.66 (2.48–2.86) | 4.90 (4.24–5.80) | 0.06 (0.03–0.12) | 0.965 | | 0.975 | | 0.892 | |
|  | Korean | 2.44 (2.27–2.52) | 4.05 (3.74–4.61) | 0.06 (0.04–0.11) | 2.45 (2.33–2.67) | 4.78 (3.92–5.59) | 0.08 (0.04–0.11) | **0.046** | | 0.058 | | 0.666 | |
|  | Chinese | 2.53 (2.44–2.76) | 5.59 (4.78–7.95) | 0.09 (0.08–0.12) | 2.65 (2.44–2.83) | 5.80 (4.82–6.92) | 0.11 (0.06–0.16) | 0.216 | | 0.229 | | 0.720 | |
|  | Finnish | 2.69 (2.49–3.23) | 6.40 (4.76–7.91) | 0.11 (0.05–0.17) | 2.58 (2.40–2.93) | 4.87 (3.99–6.24) | 0.12 (0.05–0.18) | 0.947 | | 0.479 | | 0.296 | |
|  | Hebrew | 2.45 (2.39–2.53) | 3.98 (3.82–4.72) | 0.05 (0.03–0.05) | 2.49 (2.41–2.68) | 4.26 (3.81–4.94) | 0.07 (0.02–0.11) | 0.142 | | 0.249 | | 0.231 | |
|  | *Other* | 2.42 (2.29–2.67) | 3.95 (3.22–4.99) | 0.09 (0.04–0.14) | 2.52 (2.37–2.72) | 4.63 (3.85–5.22) | 0.07 (0.03–0.14) | 0.448 | | 0.688 | | 0.781 | |
| **Total** | | 2.58 (2.40–2.81) | 4.88 (3.95–5.93) | 0.09 (0.05–0.14) | 2.59 (2.42–2.85) | 4.92 (4.00–5.97) | 0.08 (0.04–0.13) | **0.001** | | | **0.002** | **0.002** | |

Composite score (C-score) and modified *H*-index were with self-citations excluded. Mann-Whitney (*U*) test was used with a significance level *p.* ≤ 0.05.

**Table S6.** Scholar-level Analysis: Academic Age Stratification of Dental Scholars in the *Career-Long* Stanford-Elsevier Lists (SEL) of Top 2% Scientists Worldwide (2017–2023)

| **Country** | **SEL 2017** | **SEL 2018** | **SEL 2019** | **SEL 2020** | **SEL 2021** | **SEL 2022** | **SEL 2023** | **Total ▲** |
| --- | --- | --- | --- | --- | --- | --- | --- | --- |
| YEM | *NA* | *NA* | *NA* | *NA* | *NA* | 9 (9–9) | 10 (10–10) | 9.5 (9.25–9.75) |
| EGY | *NA* | *NA* | 14 (14–14) | 13.5 (12.25–14.75) | 13 (12.5–13.5) | 15.5 (12.5–20.75) | 16 (14–19) | 14 (12.25–17.5) |
| MLT | 14 (14–14) | *NA* | *NA* | *NA* | *NA* | *NA* | *NA* | 14 (14–14) |
| SAU | *NA* | *NA* | 28 (28–28) | 19 (13.5–24.5) | 20 (14.5–25.5) | 10 (9.5–21) | 16 (11–27) | 16 (10–29) |
| HRV | *NA* | *NA* | *NA* | 16 (16–16) | 16 (16–16) | 17 (17–17) | 17 (17–17) | 16.5 (16–17) |
| IRN | *NA* | *NA* | 17 (16–21.5) | 21 (17.5–24.75) | 22 (18.5–25.75) | 17 (16.5–23) | 18.5 (16.5–22.5) | 18.5 (17–25.75) |
| ISL | *NA* | *NA* | 16 (16–16) | 17 (17–17) | *NA* | 19 (19–19) | 33.5 (26.25–40.75) | 19 (17–19) |
| SRB | *NA* | *NA* | *NA* | *NA* | 18 (18–18) | 19 (19–19) | 20 (20–20) | 19 (18.5–19.5) |
| BGR | *NA* | *NA* | *NA* | *NA* | *NA* | 19 (19–19) | 20 (20–20) | 19.5 (19.25–19.75) |
| IND | 35 (35–35) | *NA* | *NA* | 23.5 (20.25–26.75) | 19 (18–27.5) | 22 (16–23) | 20 (17–23) | 20 (17–27) |
| NGA | *NA* | *NA* | *NA* | 38 (38–38) | 21 (20–29.5) | 22 (21–30) | 23 (22–30.5) | 22.5 (21–38) |
| KOR | 22 (22–22) | 25 (23–27) | 23 (18–30) | 24 (21.5–29) | 25 (22–30) | 25 (21.75–28.75) | 25 (21.5–27.5) | 24 (21–29.5) |
| LIE | *NA* | 23 (23–23) | 23 (23–23) | 25 (25–25) | 26 (26–26) | 27 (27–27) | 27 (27–27) | 25.5 (23.5–26.75) |
| BRA | 19 (17–21) | 46 (28.25–52.5) | 24.5 (20–32) | 24 (22–32) | 25 (22–31) | 26 (23–31) | 26.5 (23–32.5) | 26 (22–34) |
| SGP | *NA* | 25 (24.75–30) | 26 (25.5–26) | 26 (25–26.5) | 27 (26–27.5) | 27.5 (26.75–28.25) | 29 (27–30) | 26 (25–28.25) |
| TWN | 20.5 (19.25–21.75) | 21 (20.5–23) | 24 (20.5–31.5) | 25.5 (22.25–31.25) | 27 (24–31) | 28.5 (24.25–33.25) | 29 (26–33) | 26 (22–32) |
| JOR | *NA* | *NA* | 17 (17–17) | 24 (24–24) | 27 (27–27) | 28 (28–28) | 29 (27–35.5) | 27 (24.5–28.5) |
| PRI | 27 (27–27) | *NA* | *NA* | *NA* | *NA* | *NA* | *NA* | 27 (27–27) |
| PAK | *NA* | *NA* | *NA* | *NA* | 23 (23–23) | 29 (29–29) | 30 (30–30) | 29 (26–29.5) |
| LBN | *NA* | *NA* | *NA* | 28 (28–28) | 30 (30–30) | 30 (30–30) | 29 (29–29) | 29.5 (28.75–30) |
| POL | *NA* | *NA* | *NA* | 29 (29–29) | 30 (30–30) | 31 (31–31) | *NA* | 30 (29.5–30.5) |
| CHN | 20 (20–20) | 32 (32–32) | 32 (30–40) | 30 (26.5–35) | 31.5 (29–35.5) | 31 (26–34.5) | 31 (22–35) | 31 (26.5–35.25) |
| DEU | 36 (30–39.5) | 34 (28.5–39.5) | 30.5 (24.25–39) | 30.5 (24–38.75) | 31 (24.75–37) | 32 (25–38) | 32 (24.5–39) | 31 (25–39) |
| HKG | 28 (22–29.5) | 31 (24.5–31) | 30 (23.5–31) | 31.5 (28–34.5) | 31 (25–34.5) | 33 (27–37) | 32.5 (26.5–36) | 31 (25–35) |
| AUT | 45 (45–45) | 37 (33–41) | 28.5 (25.5–39.5) | 31 (26–44.5) | 32 (27–47) | 30.5 (26.75–47.25) | 34 (27–48) | 31.5 (26.75–46.25) |
| RUS | *NA* | *NA* | *NA* | *NA* | *NA* | 45 (31–49.5) | 18 (18–18) | 31.5 (17.75–47.25) |
| GEO | *NA* | *NA* | 32 (32–32) | *NA* | *NA* | *NA* | *NA* | 32 (32–32) |
| MYS | *NA* | *NA* | *NA* | *NA* | 32 (32–32) | 39 (35.5–42) | 32 (27.5–36) | 32 (32–39.5) |
| TUR | 41 (41–41) | 42 (42–42) | 43 (43–43) | 27 (23.5–31.25) | 28 (22.75–32.25) | 31.5 (24.5–33.25) | 33.5 (28.75–40.75) | 32 (25–37.5) |
| CHE | 33 (28.75–40.25) | 33 (29.75–38.25) | 32 (29–39) | 32.5 (29.25–39) | 33 (25.5–39) | 34 (27.75–40) | 34 (27–40) | 33 (28–39.5) |
| ITA | 31.5 (29–35) | 32.5 (29.75–34) | 31 (26–34) | 32 (26–35) | 33 (27–37) | 33.5 (27–37) | 33 (25–38) | 33 (26–37) |
| ARE | *NA* | 37 (33.5–39) | 31 (29.5–37) | 34 (30.5–38.25) | 34 (30.5–38.25) | 33 (29.5–37.5) | 34 (29.5–37.5) | 33.5 (30–39) |
| ESP | 40.5 (35.75–45.25) | 34 (31.5–42.5) | 34.5 (30–43.25) | 34 (31–37) | 32 (29–38) | 33 (30–38.5) | 34 (31–37.75) | 34 (30–38) |
| IRL | 38 (35.5–38.5) | 39 (39–39) | 38 (33–41) | 36 (24–41.25) | 34 (27–41) | 33 (22–39) | 33 (24–38.5) | 34.5 (23.5–39) |
| GBR | 34 (29–40.75) | 35 (30–43) | 35 (29.25–42) | 35 (29–42) | 36 (29–42) | 36 (30–44) | 36 (30–43) | 35 (30–42) |
| ZWE | *NA* | *NA* | *NA* | 34 (34–34) | 35 (35–35) | 36 (36–36) | 37 (37–37) | 35.5 (34.75–36.25) |
| AUS | 40 (34.5–43) | 39 (31–45.5) | 40 (31–48) | 35.5 (27.75–45.25) | 34.5 (27–45.25) | 31 (28–43) | 33 (29–45) | 36 (28–46) |
| BEL | 33 (28.5–36.5) | 36 (30.75–37.25) | 36 (31–39) | 35 (28–39) | 35 (29.25–39.75) | 36 (30–41) | 36 (30–42) | 36 (30–40) |
| FIN | 33.5 (28.75–42.75) | 33 (29–42) | 37 (29.5–45) | 36 (30.5–44.5) | 37 (30.75–45) | 36.5 (31–46) | 37 (31.5–45.5) | 36 (30.75–45) |
| NLD | 35 (33–41.75) | 36 (34–42) | 36 (33–42) | 34.5 (32–42) | 35 (32.25–42) | 36 (32–42) | 36 (32–42) | 36 (32–42) |
| PRT | *NA* | *NA* | 33 (33–33) | 34 (34–34) | 38.5 (36.75–40.25) | 39.5 (37.75–41.25) | 37 (37–37) | 36 (34.5–39.5) |
| QAT | *NA* | *NA* | 36 (36–36) | 33 (33–34.5) | 34 (34–35) | 35 (35–35.5) | 36 (36–39) | 36 (34–36) |
| KWT | 31 (30.5–37) | 37.5 (34.75–40.25) | *NA* | 41 (41–41) | *NA* | *NA* | *NA* | 36.5 (31.25–42.5) |
| GRC | 34 (34–34) | *NA* | 36 (35–37) | 36 (24–38) | 37 (29.75–39.5) | 37 (28–40) | 38 (30.25–42.5) | 37 (28.25–39.75) |
| JPN | 33.5 (29.5–37) | 37 (32–41) | 37 (33–41) | 36 (32–40.75) | 37 (32.5–42) | 38 (34–42.75) | 37 (34–41.5) | 37 (33–41) |
| NZL | 28 (25–36.5) | 42 (35.5–43.5) | 44 (33.5–46.5) | 35 (31.75–41.75) | 37 (28–42) | 36 (32–42.5) | 38 (37–41) | 37 (31.5–44) |
| ISR | 41 (35–43) | 40 (37.25–47) | 37.5 (32.5–43.25) | 37.5 (33.75–42.75) | 37 (33–42) | 38 (34–44) | 39 (34–45.25) | 38 (34–44) |
| CAN | 36 (33.25–40) | 39 (35–45) | 39 (33.25–45) | 38 (33–44.25) | 39 (33–45.25) | 40 (34–46) | 40 (32–47) | 39 (33–46) |
| CHL | *NA* | *NA* | 38 (38–38) | 39 (39–39) | 39 (39–39) | 39 (39–39) | 39 (39–39) | 39 (39–39) |
| CYP | *NA* | *NA* | *NA* | *NA* | 38 (38–38) | 39 (39–39) | 40 (40–40) | 39 (38.5–39.5) |
| NOR | 38 (36–45) | 40.5 (37.5–46.25) | 40 (34.5–46.5) | 38.5 (33.75–43.75) | 39 (35–43) | 39 (35.25–43.75) | 38 (35–44.75) | 39 (35–45.75) |
| USA | 38 (32–44) | 39 (33–45) | 39 (32–45) | 39 (32–45) | 39 (32–46) | 40 (32–46) | 40 (33–47) | 39 (32–46) |
| DNK | 39 (33.5–46) | 41.5 (35.25–47.75) | 39 (34–48) | 39.5 (33–47) | 40 (32.75–49.25) | 40 (34–49.5) | 40 (33–49) | 40 (33–48) |
| SWE | 40 (32.75–45.25) | 40.5 (34.5–45.25) | 40 (33–45) | 40 (32–45) | 40 (33–45) | 40 (33–45) | 40 (33–45) | 40 (33–45) |
| ROU | *NA* | *NA* | 39 (39–39) | 42 (42–42) | *NA* | *NA* | *NA* | 40.5 (39.75–41.25) |
| LAO | *NA* | *NA* | *NA* | *NA* | *NA* | 41 (41–41) | *NA* | 41 (41–41) |
| FRA | 46 (44–46) | 45 (43–46) | 45 (41.5–48) | 46 (33.5–47) | 37.5 (23.75–47) | 38.5 (26.25–48) | 42 (26–49) | 44 (27–48) |
| MEX | *NA* | *NA* | 35 (32–38) | 49 (48–50) | 47 (47–47) | 35 (29–41) | 37 (37–37) | 44 (35–47) |
| SVN | *NA* | *NA* | 42 (42–42) | 44 (44–44) | 44 (44–44) | 44 (44–44) | 44 (44–44) | 44 (44–44) |
| ARG | *NA* | *NA* | *NA* | *NA* | 45 (45–45) | 45 (45–45) | 46 (46–46) | 45 (45–45.5) |
| IDN | *NA* | *NA* | 46 (46–46) | *NA* | *NA* | *NA* | *NA* | 46 (46–46) |
| ZAF | 39 (34.5–43.5) | 41 (37.5–44.5) | 50 (44.5–52.25) | 50.5 (49.25–51.75) | 48 (37–52) | 42.5 (28.75–51.75) | 43.5 (30–51.75) | 48 (34–52.5) |
| OMN | *NA* | *NA* | *NA* | 52 (52–52) | 52 (52–52) | *NA* | *NA* | 52 (52–52) |
| HUN | *NA* | *NA* | *NA* | 53 (47.5–61) | 48.5 (46.25–50.75) | 53 (48.5–64.5) | 46 (44–49.5) | 53 (44–53) |
| THA | *NA* | 52 (52–52) | 52 (52–52) | 55 (55–55) | 55 (55–55) | 55 (55–55) | 55 (55–55) | 55 (52.75–55) |
| *Missed* | 36 (28–42) | 35 (25.5–44.5) | 38 (33–45.75) | 34.5 (29.5–44.25) | 41 (35.5–52) | 40.5 (36–51.25) | 45 (36–54) | 37.5 (30–45) |
| **Total** | 36 (30–43) | 38 (32–45) | 37 (31–44) | 36 (30–43) | 37 (30–44) | 37 (30–45) | 37 (30–44.25) | 37 (30–44) |

Median (Interquartile Range)

**Table S7.** Scholar-level Analysis: Academic Age Stratification of Dental Scholars in the *Single-Year* Stanford-Elsevier Lists (SEL) of Top 2% Scientists Worldwide (2017–2023)

| **Country** | **SEL 2019** | **SEL 2020** | **SEL 2021** | **SEL 2022** | **SEL 2023** | **Total ▲** |
| --- | --- | --- | --- | --- | --- | --- |
| ARM | *NA* | *NA* | *NA* | *NA* | 6 (6–6) | 6 (6–6) |
| PER | *NA* | *NA* | *NA* | *NA* | 7 (7–7) | 7 (7–7) |
| VNM | *NA* | 7 (7–7) | 8 (8–8) | 9 (9–9) | 10 (10–10) | 8.5 (7.75–9.25) |
| YEM | 6 (6–6) | 9 (8–10) | 8 (8–8) | 9 (9–9) | 10 (10–10) | 8.5 (7.25–9.75) |
| KHM | *NA* | *NA* | *NA* | *NA* | 10 (10–10) | 10 (10–10) |
| BHR | *NA* | 9 (9–9) | 11 (11–11) | 12 (12–12) | 13 (13–13) | 11.5 (10.5–12.25) |
| EGY | 10 (9.5–11) | 11 (10.5–12.5) | 12 (10.5–13) | 13 (11.25–14.5) | 14 (12–16.5) | 12 (11–14) |
| SAU | 7 (5.5–18) | 8.5 (7–15.5) | 11.5 (8–15.5) | 12.5 (9–15.75) | 14 (10–18) | 12 (8–16) |
| HRV | *NA* | 16 (16–16) | 16 (16–16) | 12.5 (11–14) | 13 (11–15) | 14.5 (11.25–16.25) |
| IND | 17 (17–18) | 11.5 (10.25–17) | 13 (11–14) | 15.5 (12–21.25) | 15 (13–19.25) | 15 (12–18) |
| IRN | 15 (13–17) | 16 (15–18.75) | 17 (15–20) | 17 (12.25–19) | 14.5 (10.25–18.75) | 16 (13–19) |
| KWT | *NA* | *NA* | 24 (24–24) | 15 (15–15) | 16 (16–16) | 16 (15.5–20) |
| ARE | 30 (21–37) | 31 (26.25–33) | 23 (14.5–31.5) | 16 (15–26.75) | 16 (16–17.5) | 17 (15–30) |
| PRT | 19 (19–19) | 16.5 (13.25–22.75) | 19 (15–35) | 16.5 (11.25–20) | 16 (10.5–19) | 17 (13–20) |
| CHL | 9 (9–9) | 23 (14–25) | 20.5 (15.25–26) | 18 (15–27.5) | 18 (13–22) | 18 (13–26) |
| CZE | *NA* | *NA* | *NA* | 18 (18–18) | 16.5 (15.25–17.75) | 18 (16–18.5) |
| ISL | 16 (16–16) | 17 (17–17) | *NA* | 19 (19–19) | 19 (19–19) | 18 (16.75–19) |
| RUS | 14 (14–14) | 15 (14.5–25) | 16 (16–16) | 28 (25–45) | 18 (12–23) | 18 (15–28) |
| SRB | 16 (16–16) | *NA* | 18 (18–18) | 19 (19–19) | 14.5 (11.75–17.25) | 18 (16–19) |
| BGR | 25 (25–25) | 17 (17–17) | 18 (18–18) | 19 (19–19) | 20 (20–20) | 19 (18–20) |
| POL | 28 (28–28) | 22 (17–22) | 23 (17.5–26.5) | 18 (16–24) | 17 (14–19) | 19 (15–24.5) |
| CHN | 21 (14.5–28.5) | 18.5 (14.25–27.5) | 19 (15–28) | 19.5 (15–25.75) | 20.5 (15–25.75) | 20 (15–26) |
| TUR | 24 (13–24) | 19 (10.5–25.5) | 20 (11.25–26.25) | 19 (13–26) | 20 (13–24) | 20 (12.5–25.5) |
| NGA | 17 (17–17) | 20 (20–20) | 21 (21–21) | 21 (20.5–21.5) | 23 (23–23) | 20.5 (20–21.75) |
| BRA | 20 (17–24.75) | 20 (17.5–25) | 21 (18–27.75) | 22 (18–27) | 22 (19–28) | 21 (18–27) |
| JOR | 26.5 (21.75–31.25) | 22.5 (21.75–23.25) | 22.5 (20.25–24.75) | 23 (23–23) | 14.5 (13.5–21.75) | 21 (16–25.5) |
| KOR | 23 (15.5–26) | 20.5 (15–24) | 22 (16–25) | 20.5 (16–25) | 20 (16–24.25) | 21 (16–25) |
| LTU | 18 (18–18) | 19 (19–19) | 21.5 (20.75–22.25) | 23 (22–24) | 23 (22–24) | 21 (19.75–23.5) |
| SGP | 25 (13–26) | 21 (15.5–25) | 22 (16.5–26) | 19 (17–24.5) | 20.5 (14.75–27) | 21 (14.75–26) |
| BGD | *NA* | *NA* | 22 (22–22) | *NA* | *NA* | 22 (22–22) |
| MYS | 8 (8–8) | 15 (12–18) | 23.5 (18.25–27.5) | 29 (23–32) | 19 (12–25) | 22 (11.75–28.25) |
| IRL | 33 (26–37) | 21 (18.75–24.75) | 22 (19.75–25.5) | 24 (20.5–30.5) | 24 (20–33.75) | 23 (20–33) |
| PAK | *NA* | 23 (23–23) | 23 (23–23) | *NA* | 15 (15–15) | 23 (19–23) |
| QAT | 31 (31–31) | 33 (28.5–33.75) | 17 (14–34) | 18 (15–35) | 19 (16–31.5) | 23 (15.25–34.75) |
| DEU | 23 (15.75–28.5) | 22 (16–29.25) | 24 (17–33) | 25.5 (17–33.25) | 24 (18–32.5) | 24 (17–32) |
| TWN | 22 (17.5–26.5) | 19.5 (11.5–28.25) | 26 (19–30) | 28 (20.5–34.5) | 26 (21.5–31) | 24.5 (19–30) |
| FRA | 28 (23.25–40.25) | 29.5 (22.75–46) | 27 (17–32) | 22.5 (14.75–32) | 21 (14.75–34.5) | 25 (16–37.5) |
| IDN | 46 (46–46) | 5 (5–5) | *NA* | *NA* | *NA* | 25.5 (15.25–35.75) |
| AUT | 26.5 (23.75–32) | 24 (23–28.5) | 26 (22–35.75) | 26 (20–27) | 27 (21.25–32.25) | 26 (22.25–31.75) |
| ESP | 30 (22.5–33.75) | 26 (17.25–35.5) | 26.5 (18–35.25) | 25 (19–33) | 26 (17–34) | 26 (18–34) |
| ITA | 26 (17–32) | 26 (17–33) | 26 (16.25–34.75) | 24 (16–34) | 25 (17–35) | 26 (17–34) |
| LIE | 23 (23–23) | 25 (25–25) | 26 (26–26) | 27 (27–27) | 27 (27–27) | 26 (25–27) |
| ROU | *NA* | 33.5 (29.25–37.75) | 26 (26–26) | 21 (18–24) | 22 (19–25) | 26 (20.5–27.5) |
| HKG | 24 (21–31) | 29 (23–32.75) | 28.5 (23.25–33) | 28 (22–33) | 27 (20.25–32) | 27 (21–32) |
| CHE | 27 (17–34) | 28 (18–35) | 26.5 (18–35.5) | 30 (19.5–36) | 27 (19–36) | 27.5 (18–36) |
| AUS | 27 (23–41.75) | 27 (22–36.75) | 28 (21.5–37.5) | 28 (20–34) | 30 (22.75–38) | 28 (21–38) |
| SVN | 42 (42–42) | *NA* | 14 (14–14) | *NA* | *NA* | 28 (21–35) |
| MKD | *NA* | *NA* | *NA* | 28 (28–28) | 29 (29–29) | 28.5 (28.25–28.75) |
| MEX | 29 (29–29) | *NA* | *NA* | *NA* | *NA* | 29 (29–29) |
| BEL | 30.5 (18.75–35.25) | 29.5 (21–35) | 29 (21–34) | 30 (20.5–36) | 31 (21–37.5) | 30 (21–36) |
| LBN | *NA* | *NA* | *NA* | 30 (30–30) | 28.5 (22.25–34.75) | 30 (23–35.5) |
| NZL | 35 (31–44) | 27 (24–35) | 27 (25.25–38) | 28 (26–34.5) | 34 (27.5–39) | 30 (26–40) |
| GRC | 29 (26–32) | 31 (25–34) | 32.5 (25–39.5) | 32 (27–38.5) | 30.5 (24.25–38) | 31 (24–38) |
| CAN | 34 (23–41) | 32.5 (19.5–40.5) | 32.5 (19.75–39.75) | 31 (22–40) | 31.5 (21.75–41) | 32 (21–41) |
| GBR | 32 (24–38) | 32 (25–38) | 33 (26–39) | 33 (25–40) | 33 (27–40) | 33 (25–39) |
| JPN | 32 (27.5–37) | 33 (29–36.5) | 32 (24.75–37) | 32 (28–38) | 34 (26–38) | 33 (28–37) |
| NLD | 33 (28–38) | 34 (28.25–38) | 33 (21.5–39) | 34 (27–40) | 34 (24.5–41) | 34 (26.25–40) |
| FIN | 38 (30–44) | 33 (27.5–44.5) | 36 (28.5–41.5) | 33 (27.5–43.5) | 34 (28.5–40.5) | 35 (28–44) |
| USA | 35 (26–43) | 34 (25–42.5) | 34 (23.5–43) | 35 (23–44) | 35 (23–44) | 35 (24–43) |
| GEO | 36 (34–38) | *NA* | *NA* | *NA* | *NA* | 36 (34–38) |
| ISR | 40 (29.75–45.75) | 34.5 (21.75–38.5) | 35 (22–40) | 37 (24.75–40.5) | 37 (24.25–43.5) | 36 (23–42) |
| SWE | 35 (25.75–41) | 37 (29–42.5) | 36 (24.5–43) | 37 (29–44) | 37 (28.5–44) | 37 (27–43) |
| ZAF | 43 (38.5–47.5) | 27.5 (24.25–30.75) | 36 (25–37) | 37 (36.25–39.75) | 38 (36.5–41.25) | 37 (34–39) |
| NOR | 39 (33–46) | 38 (32–41) | 40 (35–45) | 36 (30.5–43.5) | 38 (35–45) | 38.5 (33.75–44.25) |
| DNK | 39 (32–48.25) | 37 (25.5–45) | 37 (27–48) | 40 (33–49) | 39.5 (29.25–47.25) | 39 (29–48) |
| THA | 52 (52–52) | 40 (27.5–47.5) | 16 (5–36) | 43 (27–49) | 40.5 (39.25–41.75) | 39 (15.25–43) |
| LAO | *NA* | *NA* | *NA* | 41 (41–41) | *NA* | 41 (41–41) |
| HUN | *NA* | 42 (42–42) | 44 (44–44) | *NA* | 40 (40–40) | 42 (41–43) |
| JAM | *NA* | 45 (45–45) | *NA* | 47 (47–47) | *NA* | 46 (45.5–46.5) |
| *Missed* | 33 (24–45) | 30 (17–41) | 35.5 (28.75–42) | 38 (31.75–46) | 39 (29–49.5) | 35 (24–45) |
| **Total** | 31 (21–39) | 30 (20–38) | 29 (19–39) | 29 (19–39) | 28 (19–38.5) | 29 (20–39) |

Median (Interquartile Range)

**Table S8.** Individual-level Analysis: Correlation between Academic Age and Scholarly Output Metrics of Dental Scholars in the Stanford-Elsevier Lists (SEL) of Top Scientists Worldwide (2017–2023)

| **Scientometrics** | | ***Career-Long*** | | | | | | | | | | | | |
| --- | --- | --- | --- | --- | --- | --- | --- | --- | --- | --- | --- | --- | --- | --- |
|  |  | **SEL 2017** | **SEL 2018** | | **SEL 2019** | | **SEL 2020** | | **SEL 2021** | **SEL 2022** | | **SEL 2023** | | **Total** |
| Self-citations Excluded | Composite Score | 0.196 ** | 0.190 ** | | 0.237 ** | | 0.278 ** | | 0.288 ** | 0.285 ** | | 0.288 ** | | 0.257 ** |
|  | Composite Score Rank | -0.196 ** | -0.190 ** | | -0.237 ** | | -0.278 ** | | -0.288 ** | -0.285 ** | | -0.288 ** | | -0.250 ** |
|  | Total Citations | 0.087 * | 0.07 | | 0.076 ** | | 0.079 ** | | 0.089 ** | 0.081 ** | | 0.065 ** | | 0.081 ** |
|  | *H*-index | 0.092 ** | 0.069 | | 0.067 * | | 0.084 ** | | 0.102 ** | 0.098 ** | | 0.089 ** | | 0.092 ** |
|  | Modified *H*-index | 0.264 ** | 0.260 ** | | 0.260 ** | | 0.289 ** | | 0.303 ** | 0.296 ** | | 0.290 ** | | 0.286 ** |
|  | Single Authored Papers (N) | 0.363 ** | 0.367 ** | | 0.390 ** | | 0.415 ** | | 0.414 ** | 0.411 ** | | 0.423 ** | | 0.404 ** |
|  | Single Authored Papers (C) | 0.194 ** | 0.195 ** | | 0.281 ** | | 0.301 ** | | 0.313 ** | 0.300 ** | | 0.313 ** | | 0.288 ** |
|  | Single + First Authored Papers (N) | 0.295 ** | 0.295 ** | | 0.260 ** | | 0.287 ** | | 0.277 ** | 0.281 ** | | 0.280 ** | | 0.280 ** |
|  | Single + First Authored Papers (C) | -0.017 | -0.043 | | -0.013 | | -0.009 | | 0.002 | 0.009 | | 0.016 | | 0.004 |
|  | Single + First + Last Authored Papers (N) | 0.310 ** | 0.333 ** | | 0.304 ** | | 0.323 ** | | 0.298 ** | 0.285 ** | | 0.271 ** | | 0.297 ** |
|  | Single + First + Last Authored Papers (C) | 0.127 ** | 0.125 ** | | 0.136 ** | | 0.154 ** | | 0.162 ** | 0.156 ** | | 0.148 ** | | 0.152 ** |
| Self-citations Included | Composite Score | 0.166 ** | 0.149 ** | | 0.193 ** | | 0.239 ** | | 0.250 ** | 0.248 ** | | 0.249 ** | | 0.223 ** |
|  | Composite Score Rank | -0.166 ** | -0.149 ** | | -0.193 ** | | -0.239 ** | | -0.250 ** | -0.248 ** | | -0.249 ** | | -0.219 ** |
|  | Total Citations | 0.065 | 0.045 | | 0.047 | | 0.052 * | | 0.061 ** | 0.054 * | | 0.039 | | 0.054 ** |
|  | *H*-index | 0.063 | 0.038 | | 0.037 | | 0.054 * | | 0.070 ** | 0.066 ** | | 0.058 ** | | 0.061 ** |
|  | Modified *H*-index | 0.236 ** | 0.223 ** | | 0.227 ** | | 0.255 ** | | 0.269 ** | 0.262 ** | | 0.252 ** | | 0.253 ** |
|  | Single Authored Papers (N) | 0.363 ** | 0.367 ** | | 0.390 ** | | 0.415 ** | | 0.414 ** | 0.411 ** | | 0.423 ** | | 0.404 ** |
|  | Single Authored Papers (C) | 0.187 ** | 0.186 ** | | 0.272 ** | | 0.292 ** | | 0.304 ** | 0.291 ** | | 0.305 ** | | 0.280 ** |
|  | Single + First Authored Papers (N) | 0.295 ** | 0.295 ** | | 0.260 ** | | 0.287 ** | | 0.277 ** | 0.281 ** | | 0.280 ** | | 0.280 ** |
|  | Single + First Authored Papers (C) | -0.042 | -0.079* | | -0.049 | | -0.041 | | -0.030 | -0.020 | | -0.013 | | -0.027 ** |
|  | Single + First + Last Authored Papers (N) | 0.310 ** | 0.333 ** | | 0.304 ** | | 0.323 ** | | 0.298 ** | 0.285 ** | | 0.271 ** | | 0.297 ** |
|  | Single + First + Last Authored Papers (C) | 0.103 ** | 0.096 ** | | 0.102 ** | | 0.124 ** | | 0.133 ** | 0.128 ** | | 0.120 ** | | 0.123 ** |
|  | % Self-citations | -0.177 ** | -0.210 ** | | -0.255 ** | | -0.236 ** | | -0.240 ** | -0.230 ** | | -0.232 ** | | -0.232 ** |
| **Scientometrics** | | ***Single-Year*** | | | | | | | | | | | | |
|  |  | **SEL 2019** | | **SEL 2020** | | **SEL 2021** | | **SEL 2022** | | | **SEL 2023** | | **Total** | |
| Self-citations Excluded | Composite Score | 0.222 ** | | 0.239 ** | | 0.219 ** | | 0.239 ** | | | 0.241 ** | | 0.229 ** | |
|  | Composite Score Rank | -0.222 ** | | -0.239 ** | | -0.219 ** | | -0.239 ** | | | -0.241 ** | | -0.234 ** | |
|  | Total Citations | 0.006 | | -0.001 | | 0.003 | | 0.011 | | | 0.009 | | 0.008 | |
|  | *H*-index | -0.077 ** | | -0.058* | | -0.068 ** | | -0.081 ** | | | -0.077 ** | | -0.067 ** | |
|  | Modified *H*-index | 0.156 ** | | 0.198 ** | | 0.206 ** | | 0.202 ** | | | 0.193 ** | | 0.193 ** | |
|  | Single Authored Papers (N) | 0.579 ** | | 0.581 ** | | 0.584 ** | | 0.581 ** | | | 0.573 ** | | 0.580 ** | |
|  | Single Authored Papers (C) | 0.395 ** | | 0.392 ** | | 0.398 ** | | 0.397 ** | | | 0.410 ** | | 0.399 ** | |
|  | Single + First Authored Papers (N) | 0.448 ** | | 0.458 ** | | 0.456 ** | | 0.455 ** | | | 0.437 ** | | 0.452 ** | |
|  | Single + First Authored Papers (C) | -0.093 ** | | -0.107 ** | | -0.107 ** | | -0.095 ** | | | -0.092 ** | | -0.090 ** | |
|  | Single + First + Last Authored Papers (N) | 0.514 ** | | 0.543 ** | | 0.529 ** | | 0.539 ** | | | 0.523 ** | | 0.531 ** | |
|  | Single + First + Last Authored Papers (C) | 0.096 ** | | 0.094 ** | | 0.092 ** | | 0.118 ** | | | 0.118 ** | | 0.107 ** | |
| Self-citations Included | Composite Score | 0.139 ** | | 0.162 ** | | 0.148 ** | | 0.166 ** | | | 0.179 ** | | 0.163 ** | |
|  | Composite Score Rank | -0.139 ** | | -0.162 ** | | -0.148 ** | | -0.166 ** | | | -0.179 ** | | -0.166 ** | |
|  | Total Citations | -0.052 * | | -0.050 * | | -0.038 | | -0.031 | | | -0.031 | | -0.037 ** | |
|  | *H*-index | -0.149 ** | | -0.123 ** | | -0.121 ** | | -0.133 ** | | | -0.128 ** | | -0.123 ** | |
|  | Modified *H*-index | 0.066* | | 0.125 ** | | 0.129 ** | | 0.129 ** | | | 0.130 ** | | 0.122 ** | |
|  | Single Authored Papers (N) | 0.579 ** | | 0.581 ** | | 0.584 ** | | 0.581 ** | | | 0.573 ** | | 0.580 ** | |
|  | Single Authored Papers (C) | 0.382 ** | | 0.380 ** | | 0.389 ** | | 0.388 ** | | | 0.401 ** | | 0.389 ** | |
|  | Single + First Authored Papers (N) | 0.448 ** | | 0.458 ** | | 0.456 ** | | 0.455 ** | | | 0.437 ** | | 0.452 ** | |
|  | Single + First Authored Papers (C) | -0.145 ** | | -0.160 ** | | -0.155 ** | | -0.141 ** | | | -0.135 ** | | -0.136 ** | |
|  | Single + First + Last Authored Papers (N) | 0.514 ** | | 0.543 ** | | 0.529 ** | | 0.539 ** | | | 0.523 ** | | 0.531 ** | |
|  | Single + First + Last Authored Papers (C) | 0.040 | | 0.039 | | 0.044 * | | 0.072 ** | | | 0.073 ** | | 0.059 ** | |
|  | % Self-citations | -0.403 ** | | -0.397 ** | | -0.364 ** | | -0.333 ** | | | -0.335 ** | | -0.361 ** | |

Spearman’s rank correlation (ρ) was carried out with a significance level *p.* ≤ 0.05 level (*) and *p.* ≤ 0.01 level (**).

**Table S9.** Individual-level Analysis: Logistic Regression Models for Female Gender (Group Membership) among Dental Scholars in the Stanford-Elsevier Lists (SEL) of Top Scientists Worldwide (2017–2023)

| **Predictor** | ***Career-Long*** | | | | ***Single-Year*** | | | |  |
| --- | --- | --- | --- | --- | --- | --- | --- | --- | --- |
|  | **OR (CI 95%)** | ***p.*** | **aOR (CI 95%)** | ***p.*** | **OR (CI 95%)** | ***p.*** | **aOR (CI 95%)** | ***p.*** | |
| **SEL Update** (2018 *vs*. 2017) | 1.08 (0.783 – 1.491) | 0.638 | 1.205 (0.858–1.693) | 0.281 |  | | | | |
| **SEL Update** (2019 *vs*. 2017) | 1.267 (0.959 – 1.675) | 0.096 | 1.300 (0.968–1.747) | 0.081 | 1.108 (0.854–1.438) | 0.441 | 1.146 (0.862–1.525) | 0.349 | |
| **SEL Update** (2020 *vs*. 2017) | 1.465 (1.124 – 1.91) | **0.005** | 1.520 (1.141–2.025) | **0.004** | 1.145 (0.891–1.472) | 0.288 | 1.204 (0.906–1.601) | 0.201 | |
| **SEL Update** (2021 *vs*. 2017) | 1.602 (1.236 – 2.076) | **<0.001** | 1.669 (1.257–2.216) | **<0.001** | 1.105 (0.862–1.417) | 0.429 | 1.086 (0.822–1.436) | 0.561 | |
| **SEL Update** (2022 *vs*. 2017) | 1.415 (1.09 – 1.836) | **0.009** | 1.530 (1.147–2.039) | **0.004** | 1.188 (0.929–1.519) | 0.169 | 1.201 (0.908–1.588) | 0.199 | |
| **SEL Update** (2023 *vs*. 2017) | 1.633 (1.264 – 2.11) | **<0.001** | 1.825 (1.371–2.429) | **<0.001** | 1.387 (1.089–1.767) | **0.008** | 1.393 (1.053–1.842) | **0.020** | |
| **Academic Age** (*per* Year) | 0.967 (0.962–0.973) | **<0.001** | 0.968 (0.961–0.974) | **<0.001** | 0.977 (0.973–0.981) | **<0.001** |  | | |
| **Gross National Income per Capita** (*per* USD) | 1.000 (1.000–1.000) | 0.196 | 1.000 (1.000–1.000) | 0.842 | 1.000 (1.000–1.000) | **0.013** | 1.000 (1.000–1.000) | **0.027** | |
| **GDP Expenditure on R & D** (*per* %) | 1.064 (0.985–1.149) | 0.114 | 0.994 (0.884–1.117) | 0.920 | 0.998 (0.941–1.058) | 0.935 | 1.11 (1.009–1.222) | **0.033** | |
| **GDP Expenditure on Health** (*per* %) | 0.989 (0.974–1.005) | 0.182 | 1.000 (0.955–1.048) | 0.993 | 0.978 (0.965–0.991) | **0.001** | 0.95 (0.918–0.984) | **0.004** | |
| **GDP Expenditure on Education** (*per* %) | 1.238 (1.168–1.312) | **<0.001** | 1.538 (1.407–1.682) | **<0.001** | 1.102 (1.040–1.167) | **0.001** | 1.214 (1.122–1.314) | **<0.001** | |
| **Human Development Index** (*per* point) | 3.743 (0.782–17.908) | 0.098 | 0.073 (0.000–12.857) | 0.321 | 0.525 (0.222–1.246) | 0.144 | 0.004 (0.000–0.147) | **0.003** | |
| **Life Expectancy** (*per* Year) | 1.016 (0.997–1.035) | 0.092 | 1.023 (0.969–1.08) | 0.405 | 1.004 (0.989–1.020) | 0.563 | 1.021 (0.978–1.065) | 0.348 | |
| **Deciduous Caries** (*per* DALY/100K) | 0.859 (0.754–0.978) | **0.022** | 0.643 (0.499–0.827) | **<0.001** | 0.927 (0.838–1.024) | 0.137 | 0.715 (0.581–0.880) | **0.002** | |
| **Permanent Caries** (*per* DALY/100K) | 1.006 (0.999–1.012) | 0.071 | 0.976 (0.965–0.987) | **<0.001** | 1.005 (1.000–1.011) | 0.072 | 0.99 (0.981–0.999) | **0.025** | |
| **Periodontal Disease** (*per* DALY/100K) | 1.000 (0.998–1.002) | 0.846 | 0.994 (0.991–0.997) | **<0.001** | 1.001 (0.999–1.003) | 0.278 | 0.997 (0.995–0.999) | **0.015** | |
| **Oral Cancer** (*per* DALY/100K) | 1.001 (0.999–1.004) | 0.294 | 1.005 (0.998–1.011) | 0.161 | 1.001 (0.999–1.003) | 0.309 | 1.002 (0.998–1.006) | 0.294 | |
| **Edentulism** (*per* DALY/100K) | 1.001 (0.999–1.002) | 0.497 | 0.998 (0.996–1.000) | **0.043** | 1.001 (1.000–1.002) | 0.172 | 1.001 (1.000–1.003) | 0.176 | |
| **Composite Score** (*per* point) | 0.629 (0.520–0.761) | **<0.001** | 1.744 (1.191–2.555) | **0.004** | 0.727 (0.617–0.856) | **<0.001** | 0.92 (0.667–1.267) | 0.608 | |
| **Modified *H*-index** (*per* point) | 0.968 (0.959–0.978) | **<0.001** | 0.96 (0.942–0.978) | **<0.001** | 0.954 (0.925–0.984) | **0.003** | 0.986 (0.930–1.045) | 0.631 | |
| **Citations Count** (*per* point) | 1.000 (1.000–1.000) | 0.101 | 1.000 (1.000–1.000) | 0.394 | 1.000 (1.000–1.000) | **0.003** | 1.000 (1.000–1.000) | **0.029** | |
| **% Self-citations** (*per* point) | 4.293 (1.805–10.209) | **<0.001** | 2.993 (1.100–8.139) | **0.032** | 2.479 (1.360–4.517) | **0.003** | 2.625 (1.341–5.140) | **0.005** | |

**Table S10.** Individual-level Analysis: Regression Models of Scholarly Outputs of Dental Scholars in the Stanford-Elsevier Lists (SEL) of Top Scientists Worldwide (2017–2023)

| **Model** | **Predictor** | ***Career-Long*** | | | | | | | | | | | | | | | |
| --- | --- | --- | --- | --- | --- | --- | --- | --- | --- | --- | --- | --- | --- | --- | --- | --- | --- |
|  |  | **Composite Score (C)** | | | **Modified *H*-index** | | | | **Citations Count** | | | | **% Self-citations** | | | |  |
|  |  | **Adj. β (95% CI)** | ***p.*** | **Adj. β (95% CI)** | | ***p.*** | **Adj. β (95% CI)** | | | ***p.*** | **Adj. β (95% CI)** | | | ***p.*** |  |  |  |
| **I** | **Model Fit** | R^2^ = 7% | | | R^2^ = 8.5% | | | | R^2^ = 0.7% | | | | R^2^ = 5.7% | | | |  |
|  | **Gender** (Male vs. Female) | 0.01 (0 – 0.03) | 0.115 | 0.55 (0.21 – 0.88) | | **0.001** | 124.73 (-166.08 – 415.54) | | | 0.401 | 0 (-0.003 – 0.003) | | | 0.913 |  |  |  |
|  | **Academic Age** (*per* Year) | 0.01 (0.01 – 0.01) | **<0.001** | 0.17 (0.16 – 0.18) | | **<0.001** | 39.59 (29.73 – 49.45) | | | **<0.001** | -0.001 (-0.001 – -0.001) | | | **<0.001** |  |  |  |
| **II** | **Model Fit** | R^2^ = 7.3% | | | R^2^ = 9.3% | | | | R^2^ = 1% | | | | R^2^ = 7.1% | | | |  |
|  | **Gender** (Male vs. Female) | 0.01 (0 – 0.03) | 0.093 | 0.58 (0.25 – 0.91) | | **<0.001** | 144.08 (-146.32 – 434.48) | | | 0.331 | 0 (-0.003 – 0.003) | | | 0.909 |  |  |  |
|  | **Academic Age** (*per* Year) | 0.01 (0.01 – 0.01) | **<0.001** | 0.17 (0.16 – 0.19) | | **<0.001** | 41.17 (31.26 – 51.07) | | | **<0.001** | -0.001 (-0.001 – -0.001) | | | **<0.001** |  |  |  |
|  | **Life Expectancy** (*per* Year) | 0 (0 – 0) | 0.526 | 0.07 (0.02 – 0.12) | | **0.004** | 45.74 (2.38 – 89.11) | | | **0.039** | 0.002 (0.001 – 0.002) | | | **<0.001** |  |  |  |
|  | **Deciduous Caries** (*per* DALY/100K) | -0.04 (-0.05 – -0.02) | **<0.001** | -0.63 (-0.95 – -0.3) | | **<0.001** | -322.46 (-606.92 – -38) | | | **0.026** | 0 (-0.003 – 0.003) | | | 0.844 |  |  |  |
|  | **Permanent Caries** (*per* DALY/100K) | 0 (0 – 0) | 0.612 | 0.03 (0.02 – 0.05) | | **<0.001** | 8.55 (-4.89 – 21.99) | | | 0.212 | 0 (0 – 0) | | | 0.578 |  |  |  |
|  | **Edentulism** (*per* DALY/100K) | 0 (0 – 0) | 0.372 | 0 (0 – 0.01) | | **0.029** | 4.49 (1.51 – 7.46) | | | **0.003** | 0 (0 – 0) | | | **<0.001** |  |  |  |
| **III** | **Model Fit** | R^2^ = 8.5% | | | R^2^ = 9.5% | | | | R^2^ = 1.2% | | | | R^2^ = 11.7% | | | |  |
|  | **Gender** (Male vs. Female) | 0.02 (0 – 0.03) | **0.027** | 0.61 (0.28 – 0.95) | | **<0.001** | 128.13 (-163.37 – 419.63) | | | 0.389 | -0.001 (-0.005 – 0.002) | | | 0.359 |  |  |  |
|  | **Academic Age** (*per* Year) | 0.01 (0.01 – 0.01) | **<0.001** | 0.17 (0.16 – 0.19) | | **<0.001** | 42.91 (32.84 – 52.98) | | | **<0.001** | -0.001 (-0.001 – -0.001) | | | **<0.001** |  |  |  |
|  | **Life Expectancy** (*per* Year) | 0.01 (0 – 0.01) | **<0.001** | 0.09 (0.02 – 0.15) | | **0.009** | 85.39 (29.41 – 141.37) | | | **0.003** | 0.001 (0.001 – 0.002) | | | **<0.001** |  |  |  |
|  | **Deciduous Caries** (*per* DALY/100K) | -0.02 (-0.04 – -0.01) | **0.013** | -0.71 (-1.06 – -0.36) | | **<0.001** | -293.55 (-600.72 – 13.62) | | | 0.061 | -0.004 (-0.008 – -0.001) | | | **0.012** |  |  |  |
|  | **Permanent Caries** (*per* DALY/100K) | 0 (0 – 0) | **0.004** | 0.02 (0 – 0.04) | | 0.054 | 18.29 (1.91 – 34.67) | | | **0.029** | 0 (0 – 0) | | | 0.123 |  |  |  |
|  | **Edentulism** (*per* DALY/100K) | 0 (0 – 0) | **<0.001** | 0 (0 – 0.01) | | 0.342 | 4.36 (1.17 – 7.55) | | | **0.007** | 0 (0 – 0) | | | **<0.001** |  |  |  |
|  | **Gross National Income per Capita** (*per* USD) | 0 (0 – 0) | 0.207 | 0 (0 – 0) | | 0.819 | -0.02 (-0.04 – -0.01) | | | **0.001** | 0 (0 – 0) | | | **<0.001** |  |  |  |
|  | **GDP Expenditure on R & D** (*per* %) | -0.02 (-0.03 – -0.01) | **<0.001** | -0.37 (-0.59 – -0.14) | | **0.001** | -23.35 (-217.71 – 171.01) | | | 0.814 | 0.002 (0 – 0.004) | | | **0.028** |  |  |  |
|  | **GDP Expenditure on Health** (*per* %) | 0.01 (0.01 – 0.02) | **<0.001** | 0.03 (-0.05 – 0.1) | | 0.502 | 80.58 (15 – 146.16) | | | **0.016** | -0.001 (-0.002 – 0) | | | **0.008** |  |  |  |
|  | **GDP Expenditure on Education** (*per* %) | 0.02 (0.01 – 0.03) | **<0.001** | 0.25 (0.1 – 0.4) | | **0.002** | -91.16 (-224.47 – 42.14) | | | 0.180 | -0.008 (-0.009 – -0.006) | | | **<0.001** |  |  |  |
| **Model** | **Predictor** | ***Single-Year*** | | | | | | | | | | | | | | | |
|  |  | **Composite Score (C)** | | | **Modified *H*-index** | | | **Citations Count** | | | | **% Self-citations** | | | |  |  |
|  |  | **Adj. β (95% CI)** | ***p.*** | **Adj. β (95% CI)** | | ***p.*** | **Adj. β (95% CI)** | | | ***p.*** | **Adj. β (95% CI)** | | | ***p.*** |  |  |  |
| **I** | **Model Fit** | R^2^ = 5.4% | | | R^2^ = 4.1% | | | | R^2^ = 0.3% | | | | R^2^ = 11.8% | | | |  |
|  | **Gender** (Male vs. Female) | 0.01 (-0.01 – 0.03) | 0.170 | 0.04 (-0.06 – 0.13) | | 0.448 | 90.07 (39.2 – 140.95) | | | **<0.001** | -0.001 (-0.005 – 0.003) | | | 0.638 |  |  |  |
|  | **Academic Age** (*per* Year) | 0.01 (0.01 – 0.01) | **<0.001** | 0.03 (0.02 – 0.03) | | **<0.001** | -3.26 (-4.79 – -1.72) | | | **<0.001** | -0.002 (-0.002 – -0.002) | | | **<0.001** |  |  |  |
| **II** | **Model Fit** | R^2^ = 5.8% | | | R^2^ = 5.6% | | | | R^2^ = 0.6% | | | | R^2^ = 15% | | | |  |
|  | **Gender** (Male vs. Female) | 0.01 (0 – 0.03) | 0.129 | 0.05 (-0.04 – 0.14) | | 0.314 | 86.69 (35.84 – 137.54) | | | **<0.001** | 0 (-0.004 – 0.004) | | | 0.961 |  |  |  |
|  | **Academic Age** (*per* Year) | 0.01 (0.01 – 0.01) | **<0.001** | 0.03 (0.02 – 0.03) | | **<0.001** | -2.56 (-4.14 – -0.98) | | | **0.002** | -0.002 (-0.002 – -0.002) | | | **<0.001** |  |  |  |
|  | **Life Expectancy** (*per* Year) | 0 (0 – 0) | 0.994 | 0.03 (0.01 – 0.04) | | **<0.001** | -2.37 (-10.08 – 5.35) | | | 0.547 | 0.002 (0.001 – 0.002) | | | **<0.001** |  |  |  |
|  | **Deciduous Caries** (*per* DALY/100K) | -0.03 (-0.05 – -0.02) | **<0.001** | -0.06 (-0.15 – 0.04) | | 0.232 | 77.18 (25.12 – 129.23) | | | **0.004** | 0 (-0.004 – 0.004) | | | 0.971 |  |  |  |
|  | **Permanent Caries** (*per* DALY/100K) | 0 (0 – 0) | **0.006** | 0.02 (0.01 – 0.02) | | **<0.001** | 2.6 (0.25 – 4.95) | | | **0.030** | 0.001 (0 – 0.001) | | | **<0.001** |  |  |  |
|  | **Edentulism** (*per* DALY/100K) | 0 (0 – 0) | 0.926 | 0 (0 – 0) | | **<0.001** | -0.04 (-0.48 – 0.4) | | | 0.866 | 0 (0 – 0) | | | **<0.001** |  |  |  |
| **III** | **Model Fit** | R^2^ = 7.7% | | | R^2^ = 6.3% | | | | R^2^ = 0.7% | | | | R^2^ = 18.1% | | | |  |
|  | **Gender** (Male vs. Female) | 0.02 (0 – 0.03) | 0.088 | 0.06 (-0.03 – 0.15) | | 0.200 | 86.07 (35.15 – 136.98) | | | **<0.001** | -0.001 (-0.005 – 0.003) | | | 0.775 |  |  |  |
|  | **Academic Age** (*per* Year) | 0.01 (0 – 0.01) | **<0.001** | 0.03 (0.02 – 0.03) | | **<0.001** | -2.13 (-3.76 – -0.5) | | | **0.011** | -0.002 (-0.002 – -0.002) | | | **<0.001** |  |  |  |
|  | **Life Expectancy** (*per* Year) | 0 (0 – 0.01) | 0.180 | 0.02 (0.01 – 0.04) | | **0.009** | 1.19 (-8.28 – 10.66) | | | 0.805 | 0.002 (0.001 – 0.003) | | | **<0.001** |  |  |  |
|  | **Deciduous Caries** (*per* DALY/100K) | -0.01 (-0.03 – 0.01) | 0.211 | -0.06 (-0.17 – 0.04) | | 0.221 | 63.91 (6.59 – 121.23) | | | **0.029** | -0.007 (-0.011 – -0.002) | | | **0.002** |  |  |  |
|  | **Permanent Caries** (*per* DALY/100K) | 0 (0 – 0) | **<0.001** | 0.01 (0.01 – 0.02) | | **<0.001** | 3.17 (0.19 – 6.15) | | | **0.037** | 0 (0 – 0.001) | | | **0.008** |  |  |  |
|  | **Edentulism** (*per* DALY/100K) | 0 (0 – 0) | **0.002** | 0 (0 – 0) | | 0.061 | -0.07 (-0.6 – 0.46) | | | 0.808 | 0 (0 – 0) | | | **<0.001** |  |  |  |
|  | **Gross National Income per Capita** (*per* USD) | 0 (0 – 0) | **<0.001** | 0 (0 – 0) | | **0.022** | 0 (-0.01 – 0) | | | **0.003** | 0 (0 – 0) | | | **<0.001** |  |  |  |
|  | **GDP Expenditure on R & D** (*per* %) | -0.01 (-0.02 – 0.01) | 0.423 | -0.02 (-0.08 – 0.05) | | 0.606 | 10.29 (-23.6 – 44.18) | | | 0.552 | -0.008 (-0.011 – -0.006) | | | **<0.001** |  |  |  |
|  | **GDP Expenditure on Health** (*per* %) | 0.01 (0 – 0.01) | **0.002** | -0.01 (-0.03 – 0.02) | | 0.575 | 7.32 (-4.08 – 18.71) | | | 0.208 | 0 (-0.001 – 0.001) | | | 0.519 |  |  |  |
|  | **GDP Expenditure on Education** (*per* %) | 0.03 (0.02 – 0.04) | **<0.001** | 0.15 (0.1 – 0.19) | | **<0.001** | -14.15 (-41.24 – 12.93) | | | 0.306 | -0.006 (-0.008 – -0.004) | | | **<0.001** |  |  |  |
